# Characteristic and complementary chiral recognition ability of four recently developed immobilized chiral stationary phases based on amylose and cellulose phenyl carbamates and benzoates

**DOI:** 10.1002/chir.23446

**Published:** 2022-04-12

**Authors:** Takafumi Onishi, Takunori Ueda, Kenichi Yoshida, Kosuke Uosaki, Hiroyuki Ando, Ryota Hamasaki, Atsushi Ohnishi

**Affiliations:** ^1^ DAICEL Corporation, CPI Company, Analytical Tools BU Research and Development Center Arai Factory Myoko Niigata Japan

**Keywords:** comparison between coated type and immobilized type, complementary properties and solvent resistance, enantiomer resolution by HPLC, immobilized polysaccharide‐based chiral stationary phases

## Abstract

To date, various immobilized chiral stationary phases (CSPs) have been developed. The immobilized CSPs have opened up possibilities not only maintaining the high chiral recognition abilities as well as corresponding coated ones but also affording high durability to various mobile phase. This report directed to investigate enantioseparation of recently launched four immobilized CSPs with cellulose and amylose backbones under normal phase liquid chromatography conditions. Their chiral recognition abilities were compared with previously developed six immobilized CSPs. Particularly, we focused on the complementarity for chiral recognitions. Among them, amylose tris(3‐chloro‐5‐methylphenylcarbamate) CSP, namely, CHIRALPAK IG, showed notable chiral recognition abilities to various racemates. As expected, the investigated immobilized CSPs represented remarkable durability to wide range of mobile phases, whereas the corresponding coated CSPs could not be run due to the irreversible degradation. Taking advantage of unrestricted solvent compatibility, chiral separation selectivities were improved for some racemates.

## Graphical abstract

The chiral recognition ability and durability toward to polar organic solvents regarding four recently developed immobilized chiral stationary phases based on amylose tris(3‐chloro‐5‐methylphenylcarbamate), cellulose tris(3‐chloro‐5‐methylphenylcarbamate), amylose tris[(*S*)‐phenylethylcarbamate], and cellulose tris(4‐methylbenzoate) was investigated and summarized.

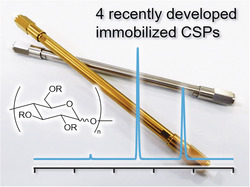

## 1 INTRODUCTION

Since the discovery that substituted polysaccharides were effective in chromatographic enantioseparation,^1^, ^2^ a number of polysaccharide, mainly cellulose and amylose, based CSPs have been developed.^3^, ^4^, ^5^, ^6^, ^7^, ^8^, ^9^, ^10^, ^11^, ^12^, ^13^, ^14^, ^15^, ^16^, ^17^, ^18^, ^19^, ^20^ Numerous separations applications of small molecule enantiomers utilizing polysaccharide‐based CSPs in both high‐performance liquid chromatography (HPLC) and supercritical fluid chromatography (SFC) mode have been reported.^8^, ^14^, ^20^ Nowadays, nearly 90% of chiral compounds can be successfully separated by using polysaccharide‐based CSPs.^6^, ^10^

**FIGURE 1:**
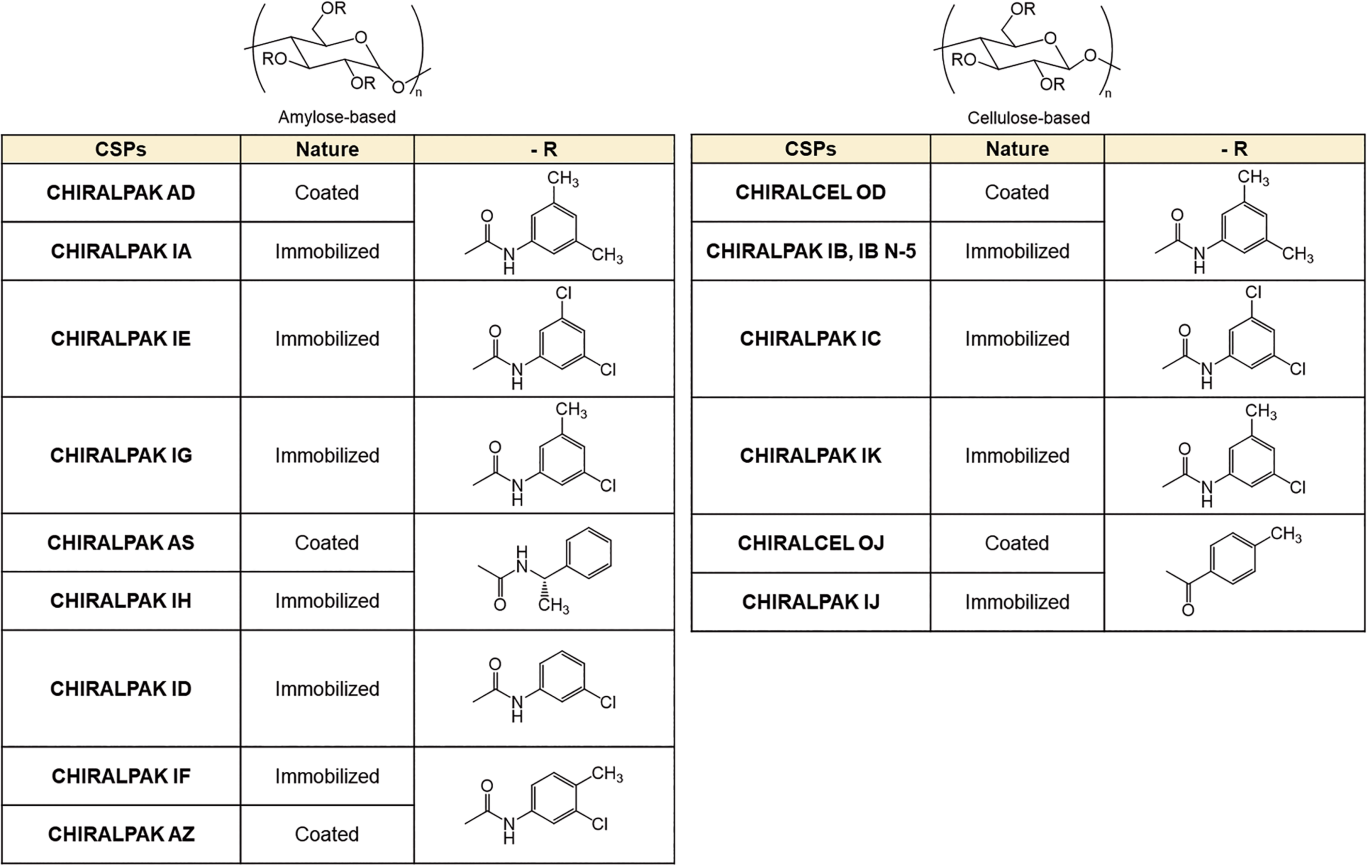
Structures of the coated polysaccharide‐derived selectors and the immobilized polysaccharide‐derived selectors

During the course of study, the mid‐1980s brought about specific reports on the excellent chiral recognition ability of coated cellulose ester derivatives.^1^, ^2^, ^21^ Among them, coated cellulose tris(3,5‐dimethylphenylcarbamate) (CDMC) and coated amylose tris(3,5‐dimethylphenylcarbamate) (ADMC) demonstrated a consistent trend of superior chiral recognition abilities to other carbamate‐based chiral selectors. These two CSPs have consistently shown an excellent chiral recognition ability to a wide range of different chiral compounds. In addition to these two coated CSPs, cellulose tris(4‐methylbezoate) (CMB) and amylose tris[(*S*)‐1‐phenylethylcarbamate] (ASPC) have emerged as having unique chiral recognition ability.^3^ Because of this, these four selectors were commercialized in the 1990s as the “Golden Four” columns from DAICEL Chemical Industries with product names CHIRALCEL OD (CDMC), CHIRALPAK AD (ADMC), CHIRALCEL OJ (CMB), and CHIRALPAK AS (ASPC) (Figure 1).^22^

Because these are all coated type CSPs, they can be easily damaged when exposed to solvents that can partially or totally dissolved polysaccharide derivatives. Consequently, polysaccharide derivatives can be removed from the silica gel support material, or can swell undesirably. Therefore, the strict restrictions are imposed on the mobile phase for these coated‐type CSPs.

To overcome this disadvantage, various immobilization methodologies were attempted. In the 2000s, the immobilized CSPs were commercially available worldwide; CHIRALPAK IA, the immobilized analog of the above‐mentioned ADMC on silica gel, and CHIRALPAK IB (and later CHIRALPAK IB N‐5), the immobilized analog of the above‐mentioned CDMC on silica gel.^23^ As expected, their durability increased to a much wider range of various solvents, as well as maintaining an excellent chiral recognition ability almost equivalent to corresponding coated ones. Taking advantage of unrestricted solvent compatibility, the broad applications and improved selectivity of a number of chiral compounds have been reported. Moreover, improved CSP robustness opened up advanced possibilities for the preparative applications.^24^

Previously, our group reported the complemental chromatographic performance of six immobilized cellulose and amylose CSPs with different structural features (CHIRALPAK IA, IB, IC, ID, IE, and IF).^25^ A series of 123 enantiomers were analyzed on the 6 columns to evaluate their chiral recognition abilities. The combination of CHIRALPAK IA + IB + IC resulted in 89% of baseline separations. When CHIRALPAK ID was added, the baseline resolution increased to 92%, and the addition of CHIRALPAK IE and IF raised the baseline resolutions to 94%. Thus, the complementarity among chiral column series would be great help for method development and optimization of chiral separation conditions.

Until 2021, additional four immobilized CSPs were launched worldwide from DAICEL Corporation. In this study, we investigated the chromatographic enantioseparation of recently developed four CSPs, which were compared with previously developed six immobilized CSPs. In particular, we focused on their characteristic and complementary. The effect of amylose or cellulose with the same carbamate on complementarity and selectivity was also discussed. The improved durability to various polar organic solvent was presented. The results will shed light on the further advancement and possibilities for method development, optimization of chiral separation conditions, and preparative applications.

## 2 EXPERIMENTAL (MATERIALS AND METHODS)

### 2.1 Chemicals (materials)

The analytical columns, CHIRALPAK® AS‐H, IA, IB, IB N‐5, IC, ID, IE, IF, IG, IH, IJ, IK, and CHIRALCEL® OJ‐H sized 4.6 mm I.D. × 250 mm L, were used as brand‐new columns from the Daicel Corporation (Tokyo, Japan). Figure 1 shows their chemical structures of chiral selectors in them. All amylose and cellulose derivatives are chemically immobilized on 5 μm silica particle. Eluents for liquid chromatography were prepared from HPLC‐grade solvents. *n*‐Hexane (*n*‐Hex), 2‐propanol (IPA), ethyl acetate (EtOAc), dimethylformamide (DMF), and tetrahydrofuran (THF) were purchased from Fujifilm Wako Pure Chemical (Osaka, Japan). Diethylamine (DEA) was purchased from Tokyo Chemical Industry Co., Ltd. (Tokyo, Japan). Ethanol (EtOH), trifluoroacetic acid (TFA), and dichloromethane (DCM) were purchased from Nacalai Tesque (Kyoto, Japan). 1,3,5‐tri‐*tert*‐butylbenzene (TTB), the void volume marker, was purchased from Sigma‐Aldrich Japan (Tokyo, Japan). Tested racemic compounds were purchased from Fujifilm Wako Pure Chemical (Osaka, Japan) and some tested racemic compounds were kindly supplied from several researchers in academia (see Acknowledgement). Structures of the tested compounds are shown in Figure S1.

### 2.2 Instruments and chromatographic conditions (general methods)

The chromatographic instrument used in this study was a Shimadzu Prominence series or Nexera series apparatus equipped with a pump, a vacuum degasser, an autosampler, a column oven, a UV detector, and LabSolutions software. Mobile phase proportions were optimized for each analysis described in Table 1, so that each racemate was eluted within a reasonable time frame. For improvement of peak shape, a basic additive (ca. 0.1 vol%) was added to eluents for basic compounds, and an acidic additive (ca. 0.1 vol%) was added to eluents for acidic compounds. The proportion of each eluent component or eluent additive was always prepared by volume. The chromatographic runs were performed at a flow rate of 1.0 ml/min and at a column temperature of 25°C, if not otherwise indicated. The column void time was measured by injecting TTB as a non‐retained marker. The resolution (*Rs*) between two enantiomers was determined by the method of mid‐height of the peaks, that is,

$$\mathrm{Rs}=\frac{\left(2.35/2\right)\left(t_{r\left(2\right)}-t_{r\left(1\right)}\right)}{\left(W_{50\left(1\right)}-W_{50\left(2\right)}\right)},$$

where tr_(1)_ and tr_(2)_ are the retention times of the first and second eluted enantiomers, respectively; W_50(1)_ and W_50(2)_ are the corresponding widths at the mid‐height of the peaks.

**TABLE 1:** Chromatographic enantioseparation results of 67 compounds with CHIRALPAK IA, IB, IC, ID, IE, IF, IG and IK

Analyte	Number	CSPs^*a*^							
		IA	IB	IC	ID	IE	IF	IG	IK
**2‐Arylpropionic acids**									
Ketoprofen	A‐1	A	D	A	A	A	A	B	A
Naproxen	A‐2	C	A	A	C	A	B	A	A
Tiaprofenic acid	A‐3	A	A	C	B	A	A	B	C
Fenoprofen	A‐4	A	C	A	D	C	B	A	C
Flurbiprofen	A‐5	A	C	C	A	A	A	A	C
2‐Phenylpropionic acid	A‐6	B	A	D	C	C	C	A	C
Ibuprofen	A‐7	C	B	C	C	C	D	C	B
Pranoprofen	A‐8	A	D	C	A	A	A	A	B
**2‐Aryloxypropionic acids**									
2‐Phenoxypropionic acid	A‐9	A	A	B	A	A	A	A	D
2‐(*o*‐Chlorophenoxy)propionic acid	A‐10	A	A	A	C	D	C	B	C
2‐(4‐Hydroxyphenoxy)propionic acid	A‐11	A	A	A	A	A	B	A	A
**Amino acids derivatives**									
N‐Cbz‐Leucine	A‐12	A	A	D	D	C	C	A	A
N‐Cbz‐Valine	A‐13	A	A	A	C	B	A	A	B
N‐Cbz‐Methionine	A‐14	A	A	B	A	B	A	A	C
N‐Cbz‐Norleuicine	A‐15	A	A	C	A	C	B	C	C
N‐Cbz‐Norvaline	A‐16	A	A	C	C	C	A	C	C
**Aryloxypropanolamines**									
Propranolol	B‐1	A	A	A	A	C	C	A	A
Propafenone	B‐2	A	C	C	A	A	A	A	C
Oxprenolol	B‐3	A	A	A	A	A	A	A	A
Alprenolol	B‐4	A	A	A	A	B	A	A	C
Pindolol	B‐5	A	A	A	C	C	A	A	A
Atenolol	B‐6	D	A	C	C	D	D	C	C
**Dihydropyridines**									
Nitrendipine	B‐7	C	D	D	A	C	C	A	C
Manidipine	B‐8	A	C	C	B	C	D	B	D
Benidipine	B‐9	B	B	B	B	C	D	A	D
Nilvadipine	B‐10	A	C	A	D	C	C	A	A
Nicardipine	B‐11	C	C	B	C	D	D	C	C
Amlodipine	B‐12	C	D	C	A	A	C	A	C
Nisoldipine	B‐13	D	D	B	B	C	D	D	A
Nimodipine	B‐14	C	C	A	A	D	C	D	A
**Benzamides**									
Cisapride	B‐15	A	C	C	A	B	A	A	A
Sulpiride	B‐16	A	D	C	B	C	B	A	D
Troxipide	B‐17	D	A	A	A	A	A	A	A
Indapamide	B‐18	B	A	B	C	B	B	A	A
**Phenothiazines**									
Profenamine	B‐19	C	C	A	B	A	B	C	D
Thioridazine	B‐20	C	D	C	B	C	C	A	D
Dimetotiazine	B‐21	A	D	C	A	A	B	A	C
Alimemazine	B‐22	D	C	B	C	D	D	C	C
**Hydantoins**									
5‐Methyl‐5‐phenylhydantoin	N‐1	A	C	C	A	A	A	A	A
Ethotoin	N‐2	A	A	A	A	C	A	C	A
**Lactones & lactams**									
γ‐Phenyl‐γ‐butyrolactone	N‐3	A	C	C	A	C	C	A	A
Pantoyl lactone	N‐4	A	C	A	A	C	A	A	A
4‐Benzoyloxy‐2‐azetidinone	N‐5	A	A	A	A	C	A	B	A
**Piperazine derivatives**									
Ranolazine	B‐23	A	A	A	A	A	A	A	A
Cetirizine	Z‐1	C	D	C	D	A	D	A	B
Hydroxyzine	B‐24	A	C	C	A	B	A	A	C
Meclizine	B‐25	A	D	D	C	C	D	A	D
**Thiazide**									
Methychlothiazide	B‐26	C	D	A	A	C	A	A	A
Cyclopenthiazide	B‐27	A	B	A	A	A	A	A	A
Ethiazide	B‐28	B	B	A	A	A	A	A	A
Benzylhydrochlorothiazide	B‐29	A	C	A	A	A	A	A	A
Mefruside	B‐30	A	D	D	A	A	A	A	C
Polythiazide	B‐31	A	D	A	A	B	A	A	A
**Others**									
Chlorprenaline	B‐32	D	D	A	B	C	C	A	A
Tulobuterol	B‐33	B	D	A	B	C	A	A	A
Labetalol	B‐34	C	D	C	C	C	C	C	C
Ritodrine	B‐35	C	D	B	C	D	D	A	B
*trans*‐Stilbene oxide	N‐12	A	A	A	A	B	A	A	A
1‐(9‐Anthryl)‐2,2,2‐trifluoroethanol	N‐13	C	A	A	C	D	C	B	A
Trögers base	N‐14	A	B	A	A	A	A	A	A
Benzoin	N‐15	A	A	A	A	D	C	A	C
Flavanone	N‐16	C	A	A	A	A	A	A	A
1‐Naphthylethanol	N‐17	D	A	A	B	D	D	C	A
Cobalt tris (acetylacetonate)	N‐18	D	D	A	A	C	D	D	A
Phenyl vinyl sulfoxide	N‐19	D	A	C	A	D	D	C	C
1,1′‐Bi‐2‐naphthol	N‐20	C	B	A	C	A	C	C	A
2‐Phenylcyclohexanone	N‐21	C	A	A	A	C	C	D	A

^a^ Each *Rs* was classified into four groups. “A” means baseline separation (*Rs* is 2 or more), “B” means nearly baseline separation (*Rs* is 1.5 or more and less than 2), “C” means partial separation (*Rs* is more than 0 and less than 1.5) and “D” means no separation (*Rs* is 0). The detail of analytical conditions and chromatographic results are shown in Table S1‐A to Table S1‐K.

The number of theoretical plates (N) was determined by the method of half peak height of the peak, that is,

$$N=5.54{\left(\frac{t_{r}}{W_{50}}\right)}^{2}.$$

## 3 RESULTS AND DISCUSSION

### 3.1 Immobilized type CSPs based on tris(3‐chloro‐5‐methylphenylcarbamate) derivatives of amylose and cellulose

CSPs based on amylose and cellulose phenylcarbamate derivatives having chloro and methyl substitutions on the benzene ring were reported by Okamoto et al. in 1990s.^26^, ^27^, ^28^ Subsequently, two versions of coated type CSPs, amylose tris(3‐chloro‐5‐methylphenylcarbamate), (ACMC) and cellulose tris(3‐chloro‐5‐methylphenylcarbamate), (CCMC) were reported.^29^, ^30^ As described in Section 1, 3,5‐dimethylphenylcarbamate substituted cellulose (CDMC) and amylose (ADMC) exhibited extremely high chiral recognition abilities. The two substituents on benzene ring of CDMC and ADMC were the same, 3, 5‐dimethyl, whereas those of ACMC and CCMC were different, 3‐chloro, 5‐methyl.

The reported coated type CSPs based on ACMC and CCMC also showed excellent chiral recognition ability like ADMC and CDMC.^29^, ^30^ In 2016, immobilized type of ACMC was commercialized as CHIRALPAK IG, and just recently (in 2021), immobilization type of CCMC was commercialized as CHIRALPAK IK.

### 3.2 Comparison of 8 kinds of immobilized type CSPs including immobilized type CSPs based on tris(3‐chloro‐5‐methylphenylcarbamate) derivatives of amylose and cellulose

To evaluate the chiral recognition abilities for recently developed columns (CHIRALPAK IG and IK), a series of 67 racemates of a broad structural variety were analyzed on *n*‐Hex/alcohol based normal phase liquid chromatography. Their chiral recognition abilities were compared with the six immobilized CSPs launched previously (CHIRALPAK IA, IB, IC, ID, IE, and IF). Note that we focused on *Rs* and α among many chromatographic parameters.

The racemates investigated here consist of 16 acidic analytes, 35 basic analytes, and 16 neutral and zwitterionic compounds. Acidic analytes include 2‐arylpropionic acids (8), 2‐aryloxypropionic acids (3), and N‐carbobenzoxy (Cbz) amino acids (5). Basic analytes include aryloxypropanolamines (6), dihydropyridines (8), benzamides (4), phenothiazines (4), piperazine derivatives (3), thiazides (6), and others (4). Neutral and zwitterionic racemates comprise hydantoins (2), lactones and lactams (3), and others (11).

**FIGURE 2:**
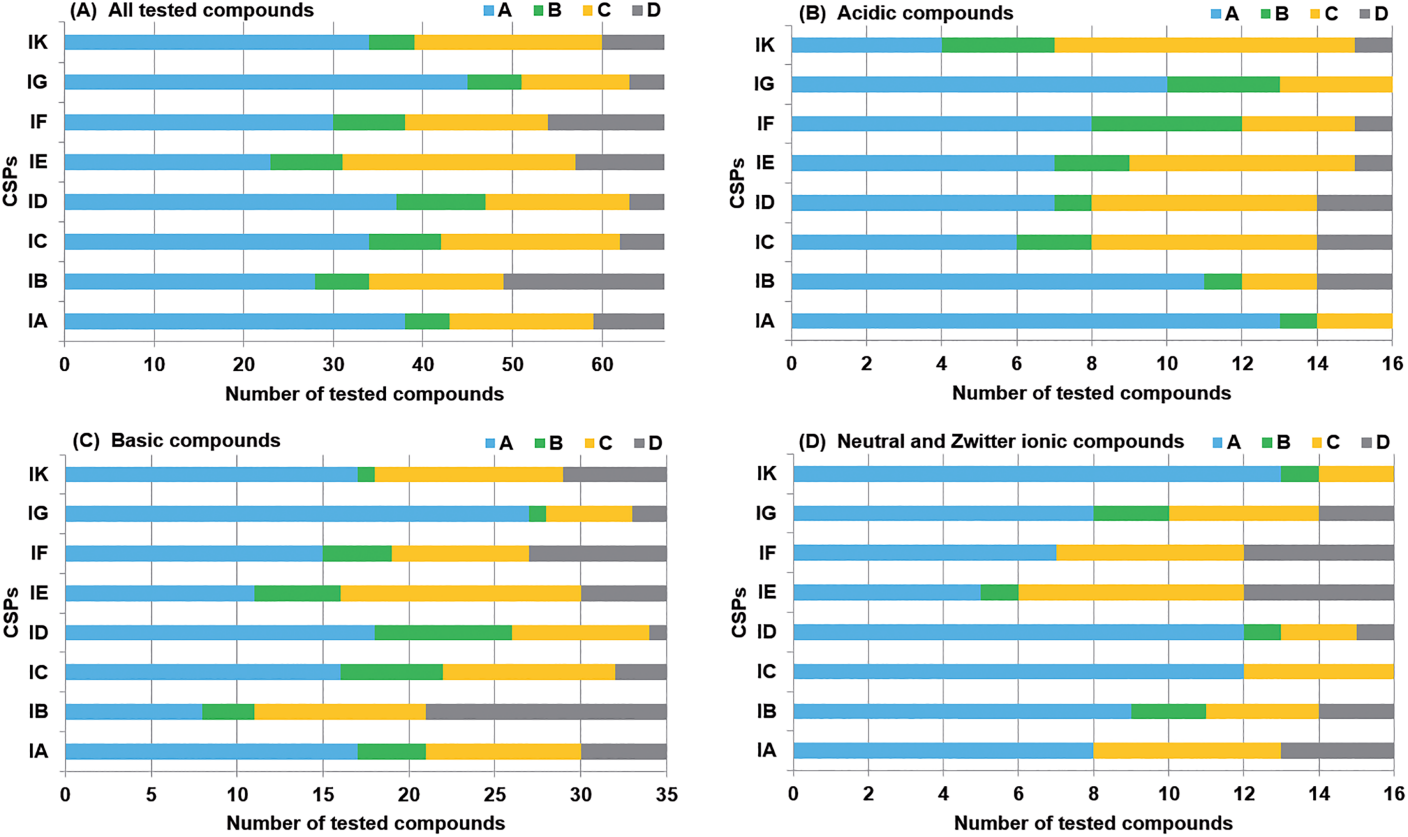
Summary of *Rs* in chromatographic enantioseparation for 67 compounds with eight immobilized chiral columns. All tested results (A), the results of acidic compounds (B), the results of basic compounds (C), and the results of neutral and zwitter ionic compounds (D). X axis is shown number of compounds, Y axis is shown kinds of CSPs. A–D represents the extent of *Rs*, which is same as Table 1

We performed the enantioseparation of 67 racemates with eight immobilized CSPs. The detail of chromatographic conditions and results are summarized in Table S1‐A to Table S1‐K. The evaluated chromatograms were analyzed from the perspective of *Rs*. Table 1 summarizes the chromatographic results, where “A” means baseline separation (*Rs* is 2 or more), “B” means nearly baseline separation (*Rs* is 1.5 or more and less than 2), “C” means partial separation (*Rs* is more than 0 and less than 1.5), and “D” means no separation (*Rs* is 0). Figure S2 shows representative chromatograms of CHIRALPAK IG and IK. The obtained data were classified based on nature of racemates (acidic, basic, and neutral/zwitterionic), which are shown in bar graph (Figure 2).

As can be seen from Figure 2A, CHIRALPAK IG showed the best separation performance. It got “A” or “B” results for 51 compounds in the 67 compounds, it showed chiral recognition to 63 compounds if “C” results included. When looking at each nature of compounds, CHIRALPAK IG showed higher separation success rate to acidic compounds (“A” or “B” results to 13 compounds in 16 compounds in Figure 2B) and basic compounds (“A” or “B” results to 28 compounds in 35 compounds in Figure 2C) compared with other CSPs. Moreover, it showed the medium separation success rate to neutral and zwitter ionic compounds among 8 CSPs (“A” or “B” results to 10 compounds in 16 compounds from in Figure 2D). It can be said that CHIRALPAK IG has a versatile and the highest chiral recognition ability for various compounds. Then, CHIRALPAK ID showed the second‐best separation performance. It got “A” or “B” results for 47 compounds in the 67 compounds, and it showed chiral recognition ability to 63 compounds if “C” results are included (Figure 2A). When looking at each nature of compounds, CHIRALPAK ID showed higher separation success rate to basic compounds (“A” or “B” results to 26 compounds in 35 compounds in Figure 2C) and neutral and zwitter ionic compounds (“A” or “B” results to 13 compounds in 16 compounds in Figure 2D) compared with other CSPs. On the other hand, it showed slightly lower separation success rate to acidic compounds (“A” or “B” results to eight compounds in 16 compounds in Figure 2B). In the third place, CHIRALPAK IA got “A” or “B” results for 43 compounds in the 67 compounds, it showed chiral recognition ability to 59 compounds if “C” results included (in Figure 2A). When looking at each nature of compounds, CHIRALPAK IA showed the highest separation success rate to acidic compounds (“A” or “B” results to 14 compounds in 16 compounds in Figure 2B), and high separation success rate to basic compounds (“A” or “B” results to 21 compounds in 35 compounds in Figure 2C). Moreover, it showed the medium separation success rate to neutral and zwitter ionic compounds among eight CSPs (“A” or “B” results to eight compounds in 16 compounds in Figure 2D).

Thus, amylose‐based CSPs (CHIRALPAK IG, ID, and IA) occupied the top 3 of this test, and it can be said that amylose‐based CSPs have versatile chiral recognition ability for various compounds.

On the other hand, for cellulose‐based CSPs, CHIRALPAK IB showed “A” or “B” results for 25 compounds in the 67 compounds (Figure 2A). When looking at acidic compounds, CHIRALPAK IB got “A” or “B” results for 12 compounds in the 16 compounds (Figure 2B), which was the third‐best in all CSPs, and the separation success rate was the highest compared with other cellulose‐based CSPs. Moreover, CHIRALPAK IK was the fifth position in eight CSPs (“A” or “B” results for 39 compounds in the 67 compounds in Figure 2A), but it showed the highest separation success rate to neutral and zwitter ionic compounds (“A” or “B” results for 14 compounds in the 16 compounds in Figure 2D).

When looking at Table 1 for each compound group, separation of thiazides has 48 patterns with a combination of compounds and CSPs (six compounds multiplied by eight CSPs is 48 patterns). In that 48 patterns, 40 cases were “A” or “B” results, the separation success rate was 80%. This indicates that thiazides are readily to separate on this eight CSPs. On the other hand, in the 64 patterns of dihydropyridines, only 23 cases were “A” or “B” results; the separation success rate was just 36%. Therefore, it can be said that dihydropyridines are difficult to separate on this eight CSPs.

Furthermore, atenolol (B‐6), nicardipine (B‐11), and alimemazine (B‐22) could be separated on only one column among eight CSPs, all of which were basic compounds. All of these compounds were separated on cellulose based CSPs; CHIRALPAK IB, IC, and IC, respectively.

In summary, amylose‐based CSPs (CHIRALPAK IG, ID, and IA) occupied the top 3 of this test, and it can be said that amylose‐based CSPs have versatile chiral recognition ability for various compounds. In contrast, cellulose‐based CSPs (CHIRALPAK IB, IC, and IK) were fifth, sixth, and eighth positions, respectively, and the separation success rate were slightly low among eight CSPs. However, cellulose‐based CSPs have specific chiral recognition ability, especially for the basic analytes that were difficult to separate on amylose‐based CSPs. Moreover, the separation success rate (baseline separation and nearly baseline separation) of 67 compounds tested this time by the use of all eight kinds of immobilized type CSPs achieved 98.5%. Hence, it has been proved that various immobilized selectors would contribute to the improvement of complementarity and the improvement of separation success rate.

These differences would be attributed to the backbone characteristics of cellulose and amylose. Although it is difficult to determine their exact structures, the helical structure of CDMC^31^ and ADMC^32^ were proposed based on two‐dimensional NMR results and calculations. We suppose that variation of the substituents on phenyl carbamate moiety combined with cellulose or amylose backbone will change the helical structure, which result in the CSP dependent specific chiral recognition.

### 3.3 Comparison of amylose‐based and cellulose‐based CSPs with 3,5‐dimethyl, 3,5‐dichloro and 3‐chloro‐5‐methyl substituted type

**FIGURE 3:**
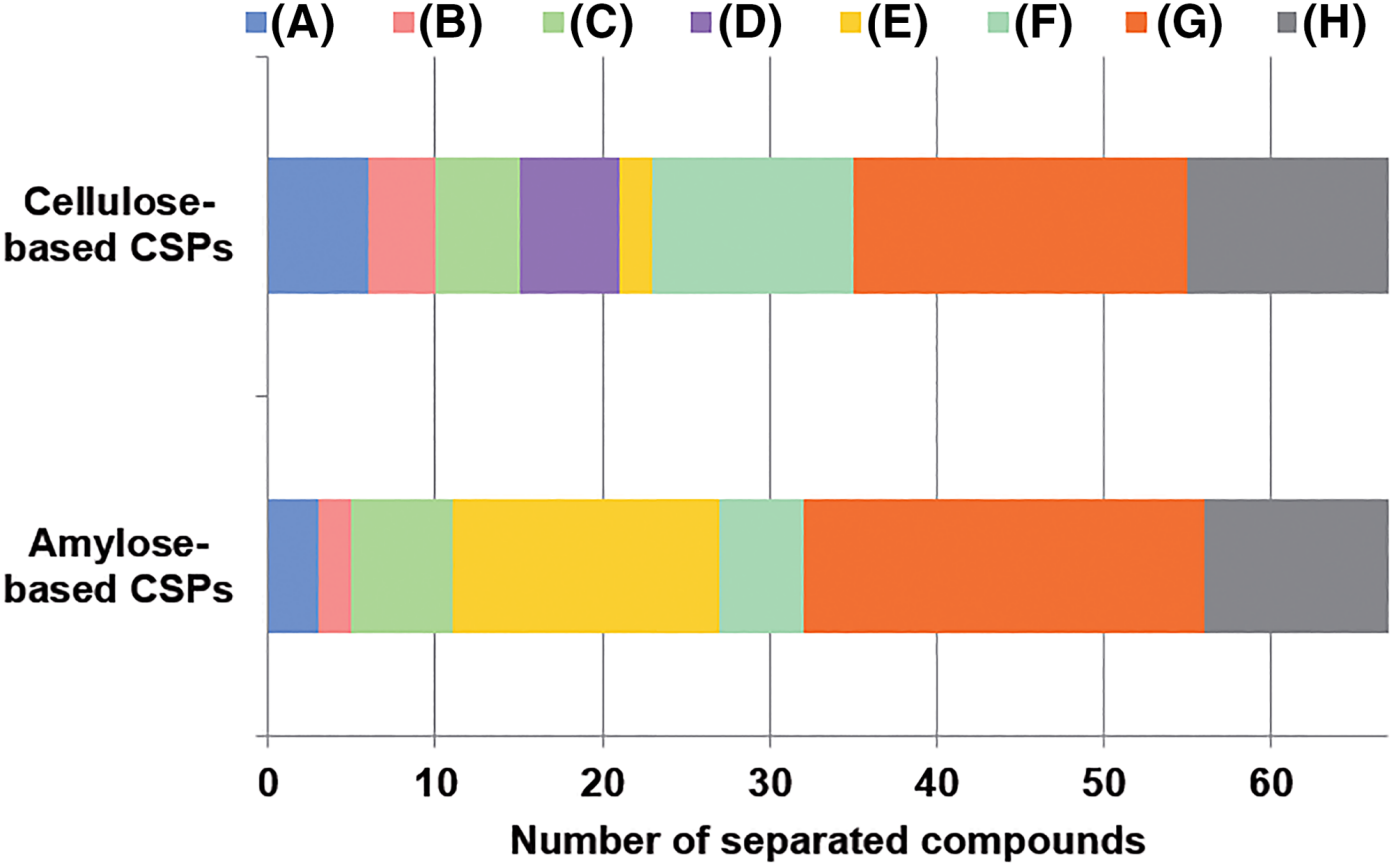
Summary of number of compounds that three kinds of CSPs based on 3,5‐dimethylphenylcarbamate (3,5‐DiMe), 3,5‐dichlorophenylcarbamate (3,5‐DiCl) and 3‐cloro‐5‐methylphenylcarbamate (3‐Cl‐5‐Me) can be separated nearly baseline separation or more. The results (A) to (H) are showed in below, (A) only separated on CSP based on 3,5‐DiMe, (B) only separated on CSP based on 3,5‐DiCl, (C) only separated on CSP based on 3‐Cl‐5‐Me, (D) separated on CSPs based on 3,5‐DiMe and 3,5‐DiCl, (E) separated on CSPs based on 3,5‐DiMe and 3‐Cl‐5‐Me, (F) separated on CSPs based on 3,5‐DiCl and 3‐Cl‐5‐Me, (G) separated on CSPs based on all 3 kinds of CSPs, (H) not separated on all three kinds of CSPs

In the previous section, we discussed the complementarity of CHIRALPAK IG and IK columns together with six immobilized columns. This section is directed to understand the influence on the resolution when amylose and cellulose possess the same phenyl carbamates. Figure 3 summarized the results according to a combination of polysaccharide types (amylose‐based CSPs and cellulose‐based CSPs) and substituent types of phenylcarbamate (3,5‐dimethyl type (3,5‐DiMe), 3,5‐dichloro type (3,5‐DiCl), and 3‐chloro‐5‐Methyl type (3‐Cl‐5‐Me)). From Figure 3, amylose‐based CSPs and cellulose‐based CSPs could be separated 56 and 55 compounds, respectively, which were very similar.

From analysis results of the chiral recognition ability of eight CSPs described in the previous section, it predicted that the group of amylose‐based CSPs shows better result than the group of cellulose‐based CSPs. However, when looking at a combination of CSPs of the same substituents, the results were very similar. Its result is presumed to be led by each different characteristic which is described in the previous section.

**FIGURE 4:**
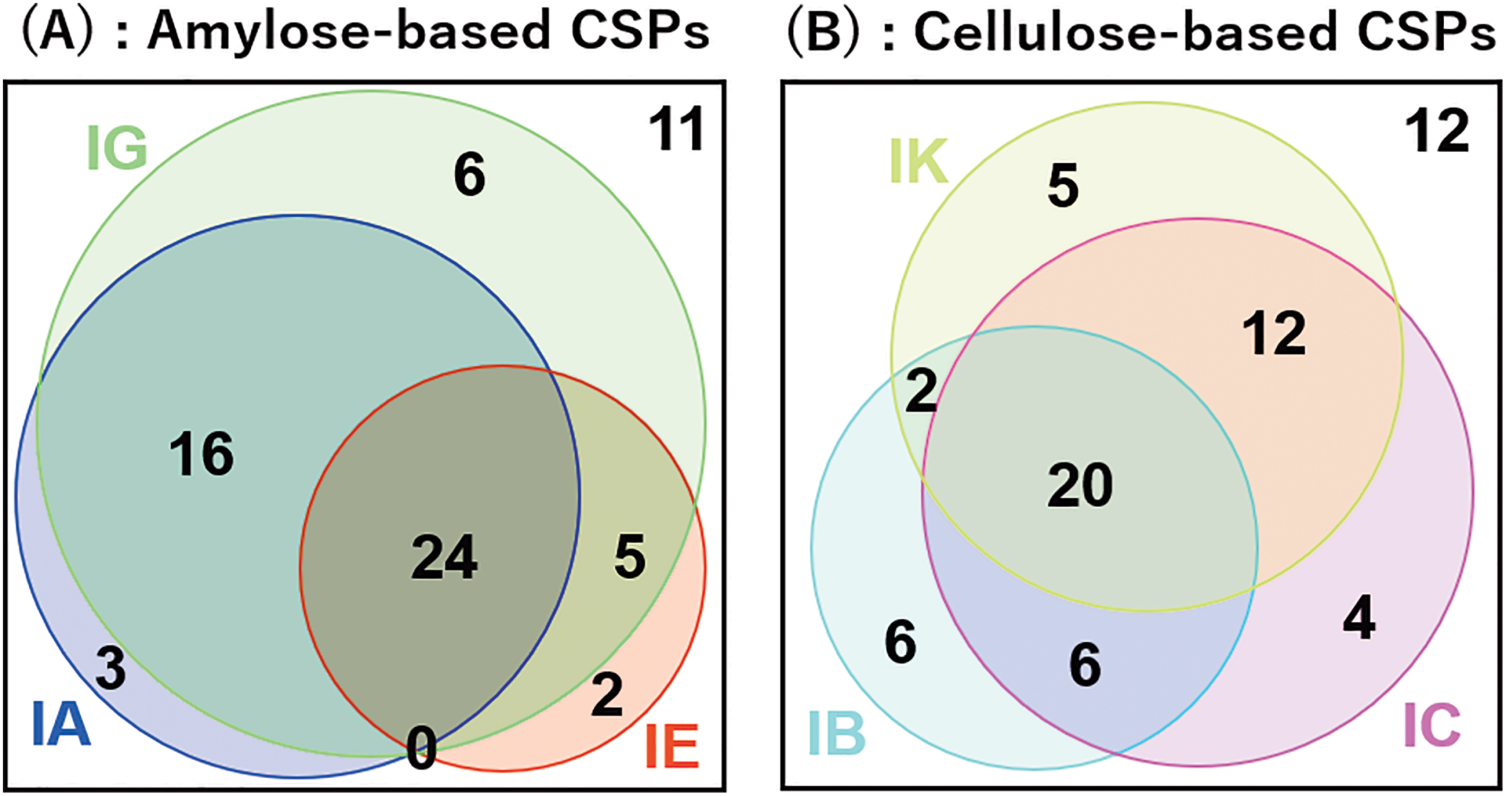
Venn diagrams of separation factor by using amylose‐based CSPs (A) and cellulose‐based CSPs (B). The number shows baseline separation and nearly baseline separation analytes (“A” or “B” in Table 1)

Figure 4A shows the relationship between chiral separation ability of three kinds of amylose‐based CSPs as a Venn diagram. As described in the previous section, CHIRALPAK IA and IG showed excellent chiral recognition ability to various kinds of compounds. Therefore, the area covered by CHIRALPAK IA and IG was large, and in particular, CHIRALPAK IG covered the largest area. On the other hand, the area covered by CHIRALPAK IE was smaller than CHIRALPAK IG and IA, but there were two compounds that could be only separated by IE. Figure 4B shows the relationship between chiral separation ability of three kinds of cellulose‐based CSPs as a Venn diagram. Compared with the amylose‐based CSPs, there were 15 compounds that could be separated on only one CSP in three kinds of cellulose‐based CSPs. This number was more than that of amylose‐based CSPs (11 compounds), therefore it can be said that the relationship of cellulose‐based each CSPs are complimentary compared with amylose‐based CSPs. When looking at each nature of compounds (Figure 2B–D), CHIRALPAK IB has an excellent chiral separation recognition to acidic compounds, CHIRALPAK IC has an excellent chiral separation recognition to basic compounds and CHIRALPAK IK has an excellent chiral separation recognition to neutral and zwitter ionic compounds. The fact that each cellulose‐based CSP has specializes in different type of compounds also demonstrate that these CSPs have complementary property. As other differed point, 3,5‐DiMe and 3‐Cl‐5‐Me type were covered large area in amylose‐based CSPs, whereas 3,5‐DiCl type was covered large area in cellulose‐based CSPs. These differences would be also attributed to the backbone characteristics of cellulose and amylose, as mentioned in Section 3.2.

### 3.4 Comparison of 3,5‐dimethyl, 3,5‐dichloro and 3‐chloro‐5‐methyl substituted type

**FIGURE 5:**
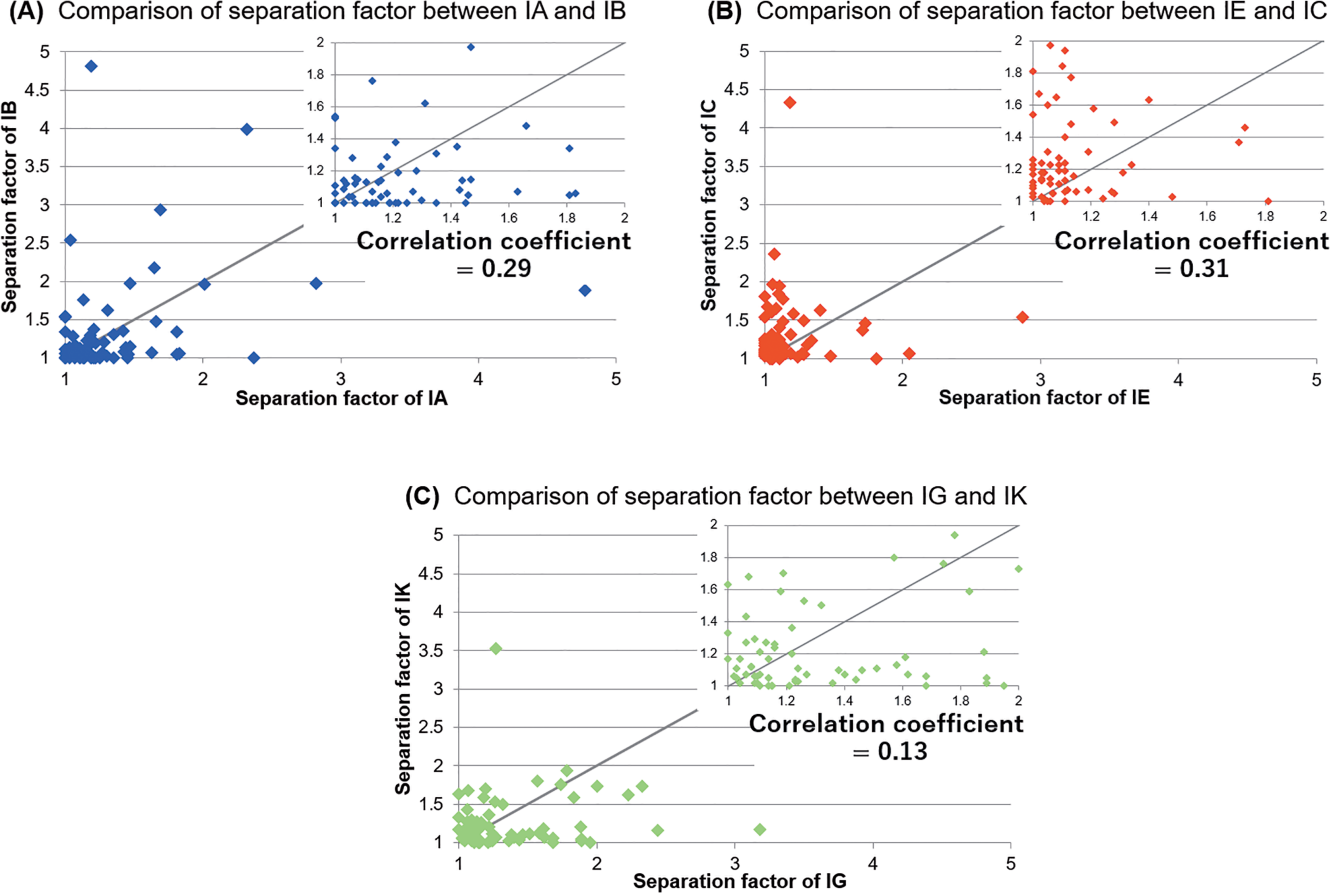
Plots of separation factor between amylose and cellulose based CSP with the same substituent. Comparison of 3,5‐DiMe (IA and IB) (A), 3,5‐DiCl (IE and IC) (B), and 3‐Cl‐5‐Me (IG and IK) (C). Inset shows expanded plots. The interrupted line is the first bisector showing equivalent separation factor

CHIRALCEL OD and CHIRALPAK AD, coated type CSPs of 3,5‐DiMe‐based cellulose and amylose, respectively, are often reported to have complementary chiral recognition abilities.^3^ We then investigated the relationship of separation factor between immobilized type CHIRALPAK AD and CHIRALCEL OD. Figure 5A shows the relationship of separation factor of CHIRALPAK IA and IB. The calculated correlation coefficient was 0.29, which suggests that there is only very weak correlation between chiral recognition abilities of CHIRALPAK IA and IB. (In generally, it is no correlation when correlation coefficient is between 0 and 0.2, it is very weak correlation when correlation coefficient is between 0.2 and 0.4.). For 2‐arylpropionic acids and benzamides in Table 1, the analytes that can be separated by CHIRALPAK IA column were not separated by CHIRALPAK IB column and vice versa. This result can be said that CHIRALPAK IA and IB have complimentary property same as the relationship of CHIRALPAK AD and CHIRALCELOD.

Calculation formula of correlation coefficient is below:

$$r=\frac{\sum \left(x-\bar{x}\right)\times \left(y-\bar{y}\right)}{\sqrt{\sum {\left(x-\bar{x}\right)}^{2}\times {\left(y-\bar{y}\right)}^{2}}}.$$

In the same way, Figure 5B shows the relationship of separation factor of CHIRALPAK IE and IC with the substituent of 3,5‐DiCl type. The calculated correlation coefficient was 0.31, which suggests that there is very weak correlation between chiral recognition abilities of CHIRALPAK IE and IC. In addition, when looking at the separation results of hydantoins and dihydropyridines in Table 1, it can be said that CHIRALPAK IE and IC have complimentary.

Relationship of separation factor of CHIRALPAK IG and IK with the substituent of 3‐Cl‐5‐Me type is shown in Figure 5C. The calculated correlation coefficient was 0.13, which suggests that there is nothing of correlation between chiral recognition abilities of CHIRALPAK IG and IK. When looking at the separation results of dihydropyridines in Table 1, it can be said that CHIRALPAK IG and IK have complimentary.

**FIGURE 6:**
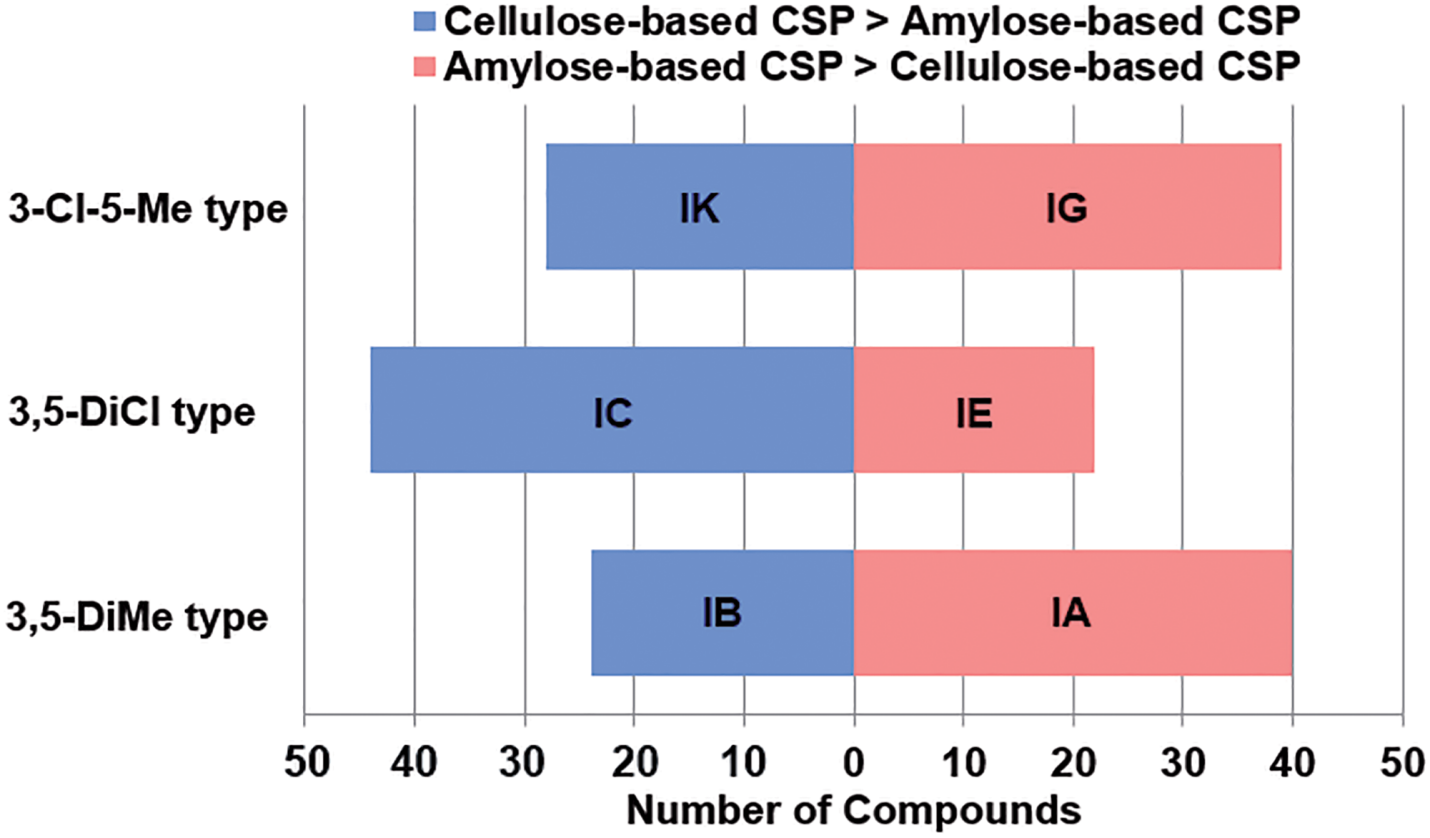
Comparison of separation factor between amylose‐based CSP and cellulose‐based CSP. The length of red bar is shown number of compounds that the separation factor of amylose‐based CSP is better than cellulose‐based CSP. The length of blue bar is shown the opposite. The comparison of 3‐Cl‐5‐Me type, 3,5‐DiCl type, and 3,5‐DiMe type are shown in order from the top of graph

Figure 6 shows comparison of separation factor between amylose‐based CSPs and cellulose‐based CSPs for each substituent. Blue bar shows the number of compounds which separation factor of cellulose‐based CSP is better than amylose‐based CSP, red bar shows number of compounds which separation factor of amylose‐based CSP is better than cellulose‐based CSP. As can be seen from Figure 6, 3‐Cl‐5‐Me and 3,5‐DiMe type amylose‐based CSP was better separation factor than that of cellulose‐based CSPs. On the other hand, for 3,5‐DiCl type, cellulose‐based CSP designates better separation factor than amylose‐based CSP. This difference in each substituent is also presumed to be due to backbone's distinction between amylose and cellulose, which is mentioned in Section 3.2.

### 3.5 Immobilized type ASPC CSP, CHIRALPAK IH and CMB CSP, CHIRALPAK IJ

The stationary phases with amylose tris[(*S*)‐1‐phenylethylcarbamate] (ASPC) and cellulose tris(4‐methylbenzoate) (CMB) as chiral selectors were reported in 1990 and 1987 by Okamoto^33^, ^34^ and were commercialized as CHIRALPAK AS and CHIRALCEL OJ, respectively. ASPC has an (*S*)‐enantiomerically pure asymmetric center on the phenyl carbamate moiety, therefore it showed unique and excellent separation characteristics for lactam, lactone, and sulfoxide compounds, especially for β‐lactams.^35^, ^36^ CMB is a cellulosic ester derivative, and its CSP has unique separation characteristics like ASPC CSP.^37^

CHIRALPAK AS and CHIRALCEL OJ are coated type CSPs. As described in Section 1, they can be easily damaged when exposed to solvents that can partially or totally dissolved polysaccharide derivatives. Consequently, polysaccharide derivatives can be removed from the silica gel support material or can swell undesirably. Therefore, the strict restrictions are imposed on the mobile phase for these coated‐type CSPs.

Numerous reports describe that this disadvantage of coated type CSPs have been resolved by the immobilization of the polysaccharide derivatives onto the silica gel support.^9^, ^13^ As the immobilized type CSP of ASPC and CMB, CHIRALPAK IH and IJ are commercialized in 2018 and 2020, respectively.

### 3.6 Comparison between coated type CSPs and immobilized type CSPs

**FIGURE 7:**
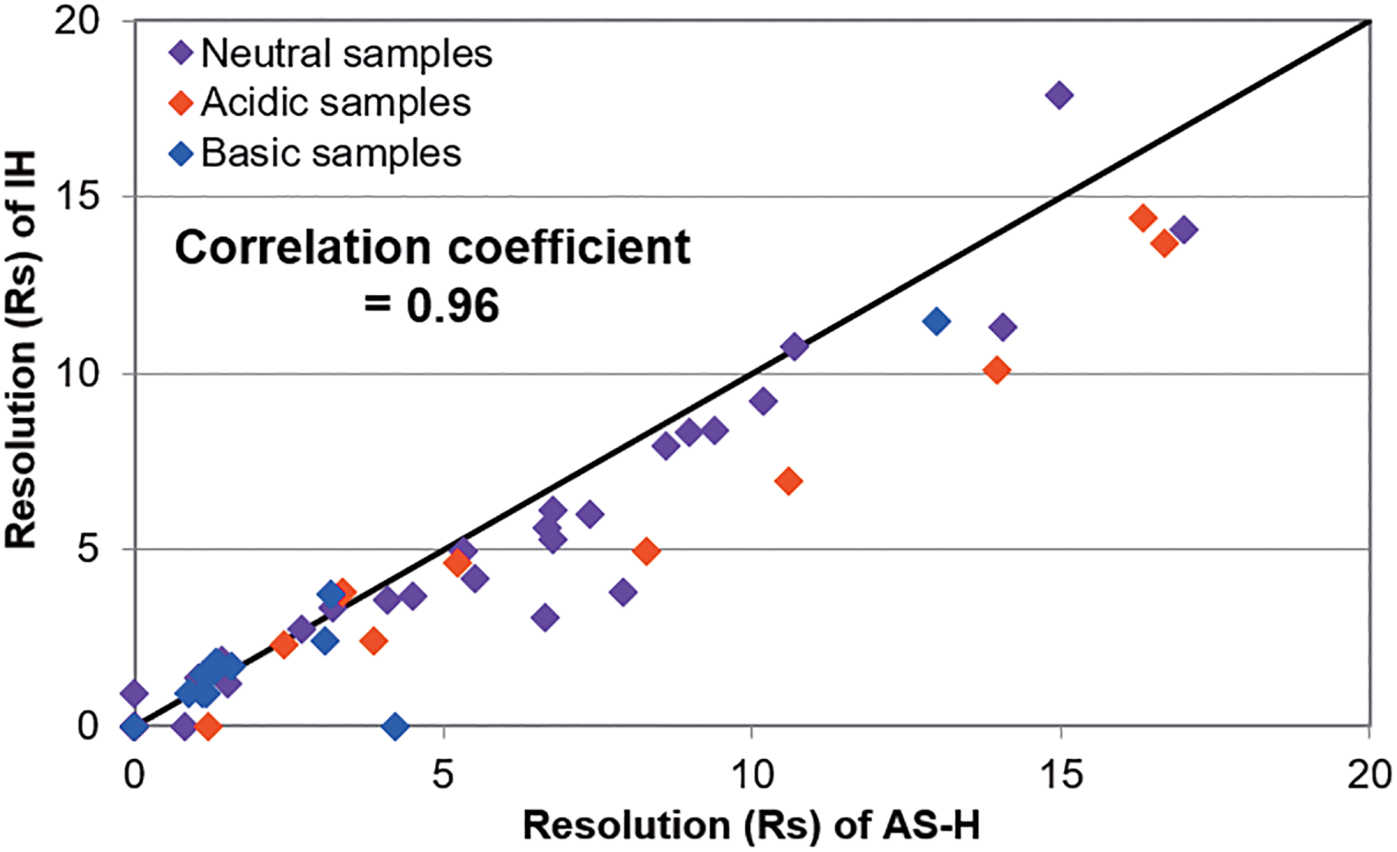
Plots of *Rs* between CHIRALPAK AS‐H and IH. The tested compounds, detail of analytical conditions and chromatographic results are in Table S2‐A and S2‐B

The difference in chiral recognition ability between immobilized ASPC CSP, CHIRALPAK IH and coated ASPC CSP, CHIRALPAK AS‐H were examined closely under normal phase conditions using 55 compounds. The resolution (*Rs*) of CHIRALPAK IH and AS‐H were plotted in Figure 7. The tested compounds, detail analytical conditions and chromatographic results were summarized in Table S2‐A and S2‐B.

As can be seen from Figure 7, the correlation coefficient between *Rs* of CHIRALPAK AS‐H and IH was 0.96; it can be said very strong correlation. This suggests that immobilized type CHIRALPAK IH could be an alternative for CHIRALPAK AS‐H if needed.

**FIGURE 8:**
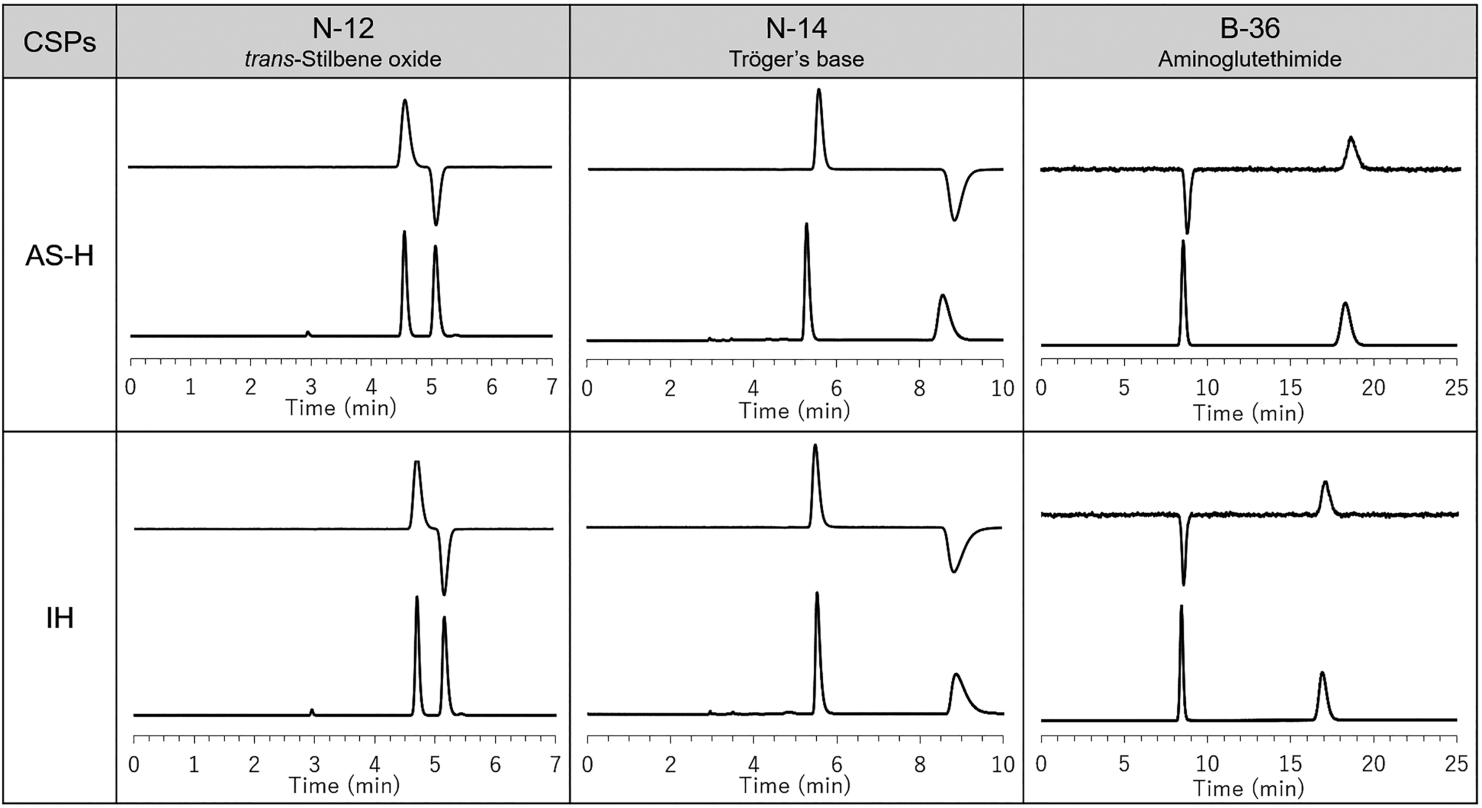
HPLC chromatograms of N‐12 (*trans*‐stilbene oxide), N‐14 (trögers base), and B‐36 (aminoglutethimide) on CHIRALPAK AS‐H and IH column using CD (upper) and UV (lower) detectors. Analytical condition of N‐12 and N‐14; eluent: *n*‐Hex/IPA 90/10 (v/v), flow rate: 1.0 ml/min, temperature: 25°C and detectors: UV 254 nm and CD 254 nm. Analytical condition of B‐36; eluent: *n*‐Hex/IPA/DEA 50/50/0.1 (v/v/v), flow rate: 1.0 ml/min, temperature: 25°C and detectors: UV 254 nm and CD 230 nm

Recently, reversal of enantiomer elution order was observed between the coated and immobilized CSPs of ACMC selectors.^38^ We then investigated the elution order on CHIRALPAK IH and AS‐H by some representative analytes. Typical chromatograms were shown in Figure 8 by using UV and CD detectors. Although it is a limited compound, the elution orders of CHIRALPAK IH and AS‐H were the same. Therefore, it is presumed that the mechanism of chiral separation between CHIRALPAK IH and AS‐H is basically the same.

**FIGURE 9:**
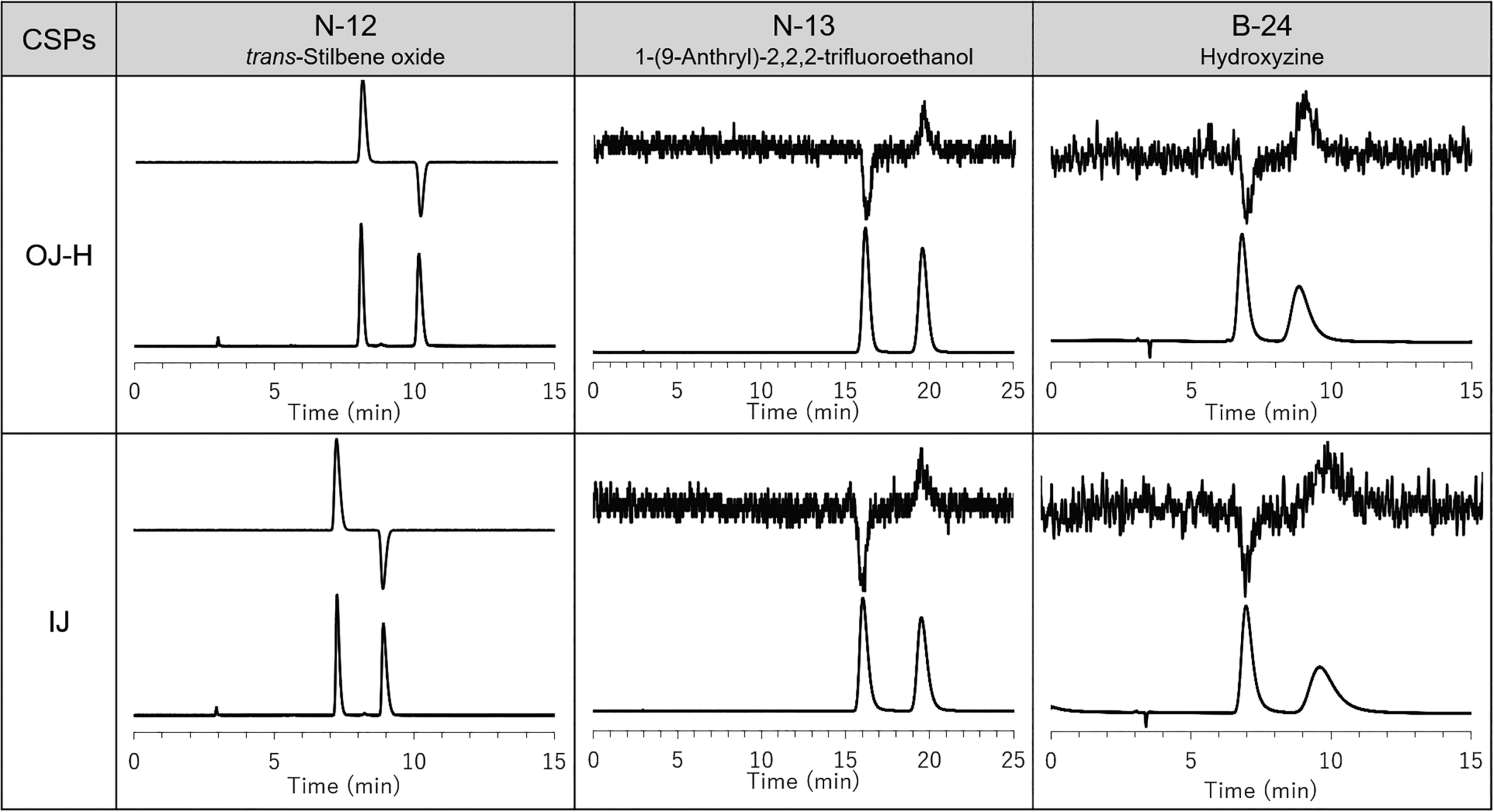
HPLC chromatograms of N‐12 (*trans*‐stilbene oxide), N‐13 (1‐(9‐anthryl)‐2,2,2‐trifluoroethanol), and B‐24 (hydroxyzine) on CHIRALCEL OJ‐H and CHIRALPAK IJ using CD (upper) and UV (lower) detectors. Analytical condition of N‐12 and N‐13; eluent: *n*‐Hex/IPA 90/10 (v/v), flow rate: 1.0 ml/min, temperature: 25°C, and detectors: UV 254 nm and CD 254 nm. Analytical condition of B‐24; eluent: *n*‐Hex/IPA/DEA 90/10/0.1 (v/v/v), flow rate: 1.0 ml/min, temperature: 25°C and detectors: UV 230 nm and CD 375 nm

In the same way, enantiomer elution order was investigated between immobilized CMB CSP, namely, CHIRALPAK IJ, and coated CMB CSP, namely, CHIRALCEL OJ‐H (Figure 9). As revealed from UV and CD detector signals, the separation tendency between CHIRALPAK IJ and CHIRALCEL OJ‐H were very similar and their elution orders were the same. Therefore, it is presumed that the mechanism of chiral separation between CHIRALPAK IJ and CHIRALCEL OJ‐H is basically the same.

### 3.7 Durability toward highly polar organic solvents

**FIGURE 10:**
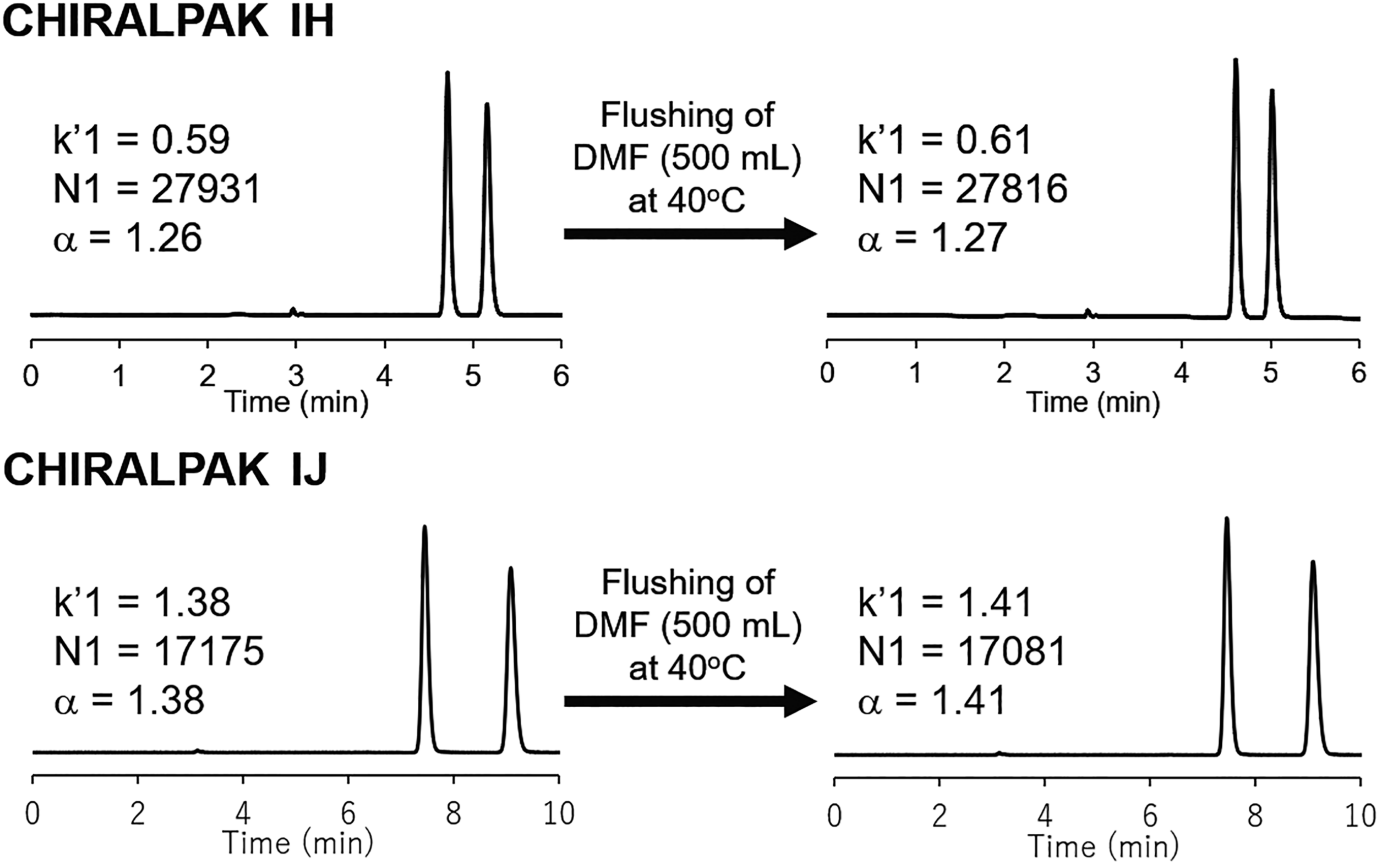
HPLC chromatogram of N‐12 (*trans*‐stilbene oxide) before and after DMF flushing (500 ml). The evaluation condition; column size: 4.6 mm I.D. × 250 mm L, eluent: *n*‐Hex/IPA 90/10 (v/v), detector: UV 230 nm, flow rate: 1.0 ml/min, temperature: 25°C

ASPC as a chiral selector for CHIRALPAK AS‐H and IH CSPs is easily solubilized in the highly polar organic solvents such as DMF and THF. CMB as a chiral selector for CHIRALCEL OJ‐H and CHIRALPAK IJ CSPs is also easily solubilized in DMF and DCM. For this reason, it is strictly forbidden that the coated type CSP CHIRALPAK AS‐H and CHIRALCEL OJ‐H contact with these solvents. On the other hand, immobilized type CHIRALPAK IH and IJ CSPs are expected to have durability against these highly polar organic solvents. In order to investigate the durability of CHIRALPAK IH and IJ columns, a large amount of DMF was flushed at 40°C. Figure 10 shows HPLC chromatogram of *trans*‐stilbene oxide (sample number: N‐12) before and after DMF flushing. Both CHIRALPAK IH and IJ, the chromatographic performance, namely, retention factor, theoretical plates number, and separation factor, did not change. These results indicated the durability of both CHIRALPAK IH and IJ significantly improved toward these polar organic solvents which were previously restricted for corresponding coated type columns.

### 3.8 New chiral recognition profiles using expanded range of organic solvents

**FIGURE 11:**
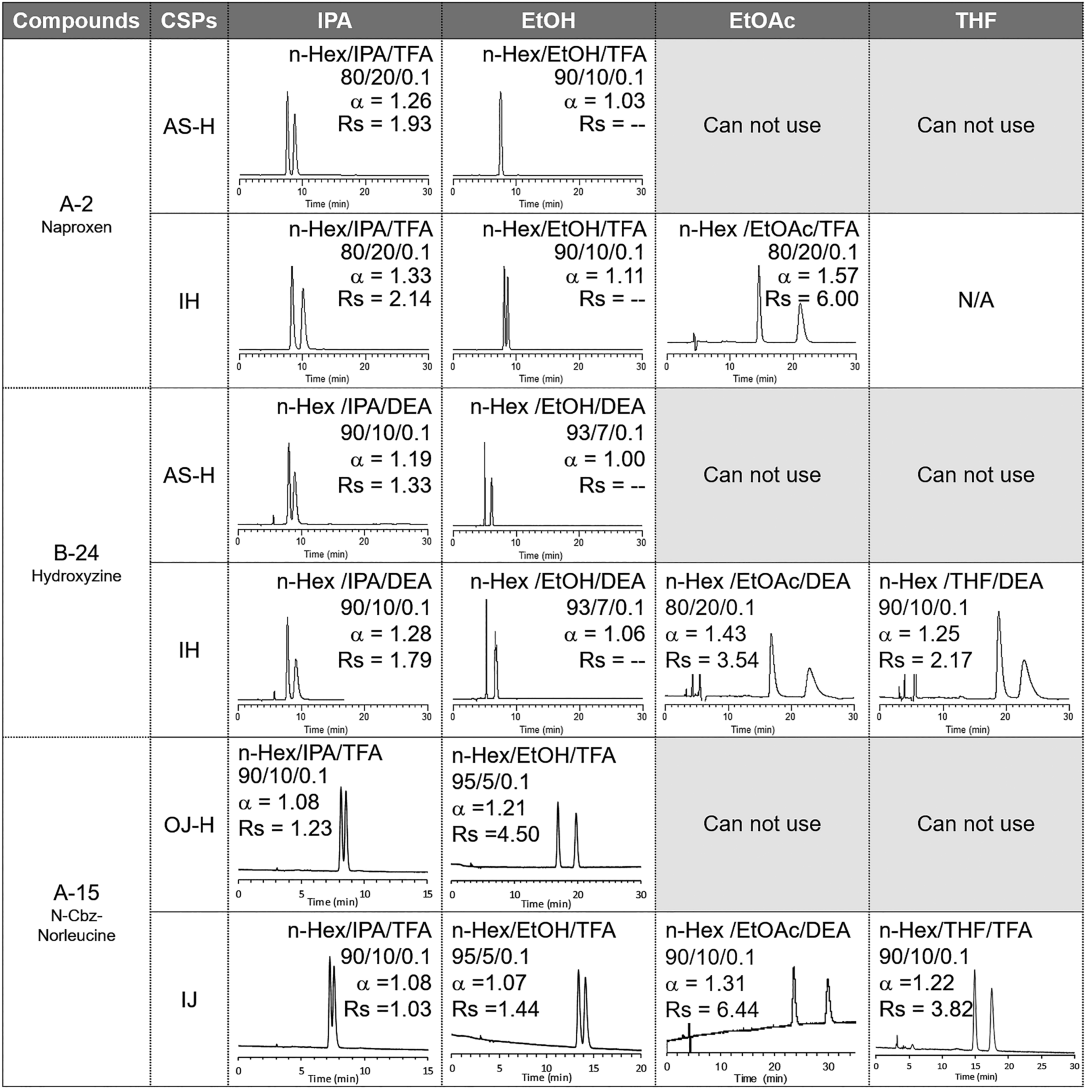
HPLC chromatograms of A‐2 (naproxen), B‐24 (hydroxyzine), and A‐15 (N‐Cbz‐norleucine) on coated type columns (CHIRALPAK AS‐H or CHIRALCEL OJ‐H) and corresponding immobilized columns (CHIRALPAK IH or CHIRALPAK IJ) under *n*‐Hex/alcohol (IPA or EtOH) based eluent

Immobilized type CSPs are well known to show new chiral recognition abilities by passing the organic solvents that could not be used for coated type CSPs due to irreversible degradation.^39^, ^40^, ^41^ As described in previous section, we can use various organic solvents for CHIRALPAK IH and IJ, such as DMF, EtOAc, and THF. As in the case for previously reported immobilized CSPs, CHIRALPAK IH and IJ showed several improved chiral separations by using aforementioned solvents (Figure 11).

For naproxen (sample number: A‐2), nearly baseline separation was achieved on CHIRALPAK AS‐H column under normal phase LC condition. When the enantioseparation by CHIRALPAK IH was performed, EtOAc could be used instead of alcohol, resulting in *Rs* value improvement about three times. Even in the case of hydroxyzine (sample number: B‐24) with CHIRALPAK IH, *Rs* was improved about two times by using EtOAc instead of IPA. Same tendency was observed for CHIRALPAK IJ. In the case of N‐Cbz‐norleucine (sample number: A‐15), *Rs* was improved about 1.5 times by using EtOAc instead of IPA.

### 3.9 Character of chiral recognition ability of IH and IJ CSPs

**FIGURE 12:**
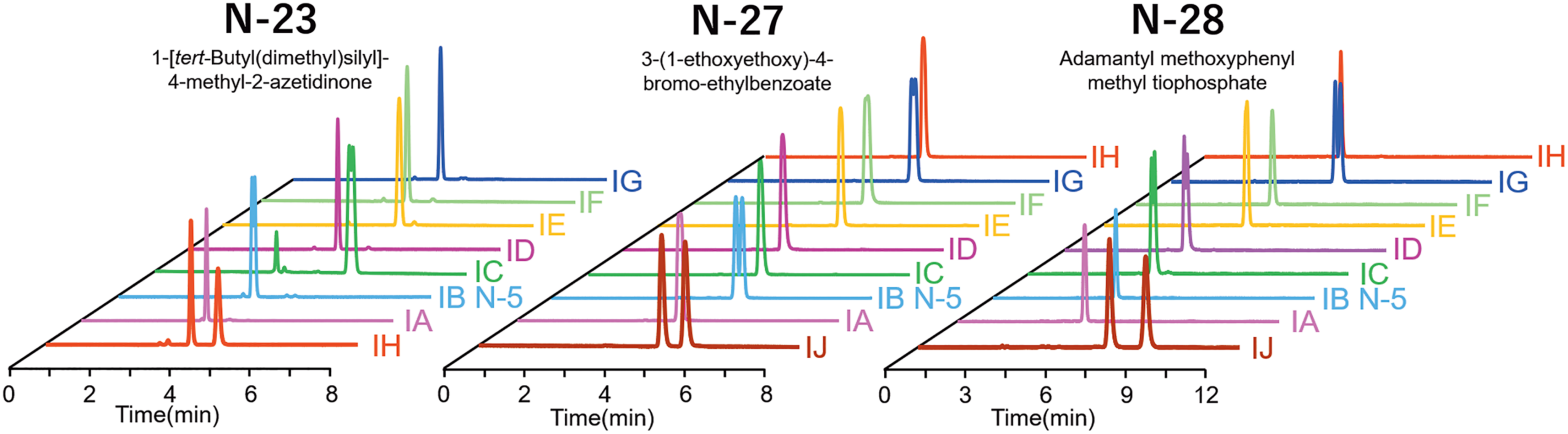
Characteristic separation results of CHIRALPAK IH and IJ. Common analytical condition: Column size is 4.6 mm I.D. × 250 mm L, flow rate is 1.0 ml/min, temperature is 25°C. additional analytical conditions for N‐23; eluent: *n*‐Hex/IPA 60/40 (v/v), detector: UV 210 nm. Additional analytical conditions for N‐27; eluent: *n*‐ex/EtOH 95/5 (v/v), detector: UV 210 nm. Additional analytical conditions for N‐28; eluent: *n*‐Hex/EtOH 90/10 (v/v), detector: UV 220 nm

As mentioned in Section 3.5, coated type CHIRALPAK AS‐H CSP showed characteristic chiral recognition abilities especially for lactams and lactones. Similar to the coated selector, corresponding immobilized column, CHIRALPAK IH showed nearly complete baseline separation success rate to β‐lactone or β‐lactam compounds. This highly chiral recognition abilities to lactams and lactones was distinctive for CHIRALPAK IH and was not found in other CSPs. Table 2 shows the separation results of 8 β‐lactone or β‐lactam compounds on eight kinds of immobilized CSPs and CHIRALPAK AS‐H. The detail analytical conditions and chromatographic results were summarized in Table S3. As shown Table 2, only CHIRALPAK IH and AS‐H were successful in separating all eight β‐lactone or β‐lactam compounds. Figure 12 shows the exemplary HPLC chromatogram of sample number: N23 on eight kinds of CSPs.

**TABLE 2:** The results of separation of 8 kinds of β‐lactone or β‐lactam compounds on 9 kinds of CSPs

	IA	IB N‐5	IC	ID	IE	IF	IG	IH	AS‐H
N‐3	+	−	−	+	−	+	+	+	+
γ‐Phenyl‐γ‐butyrolactone									
N‐4	+	+	+	+	−	+	+	+	+
Pantoyl lactone									
N‐5	+	+	+	+	−	+	−	+	+
4‐Benzoyloxy‐2‐azetidinone									
N‐22	−	−	+	−	−	−	−	+	+
4‐Ethenyl‐2‐azetidinone									
N‐23	−	−	−	−	−	−	−	+	+
1‐[*tert*‐Butyl (dimethyl)silyl]‐4‐methyl‐2‐azetidinone									
N‐24	−	−	−	−	−	−	−	+	+
1‐Benzyl‐3‐ethyl‐2‐azetidinone									
N‐25	−	+	+	−	+	+	+	+	+
3‐Dimethyl‐4‐phenyl‐2‐azetidinone									
N‐26	+	−	+	+	+	+	+	+	+
3‐Dimethyl‐4‐(2‐furyl)‐2‐azetidinone									

*Note*: “+” means *Rs* is 2 or more and “‐” means *Rs* is less than 2. The detail of analysis conditions and chromatographic results are shown in Table S3.

CHIRALPAK IJ also exhibited characteristic chiral recognition ability for the compounds which other CSPs could not resolve. Figure 12 also shows chromatograms of two compounds in which only IJ succeeded in baseline separation, whereas other eight immobilized type CSPs (CHIRALPAK IA to IH) could not be completely separated.

## 4 CONCLUSIONS

In this report, the recently developed new immobilized ACMC, CCMC, ASPC, and CMB CSPs, CHIRALPAK IG, IK, IH, and IJ, were investigated regarding several different project scopes. As results of these investigation, it was found that CHIRALPAK IG has outstanding versatility, CHIRALPAK IK has complementary chiral recognition ability to CHIRALPAK IG, and CHIRALPAK IH and IJ have chiral recognition abilities similar to their coated type CSPs, but with the added benefit of an expanded solvent resistance and durability. It was found that all four columns have complementary separation properties, which could separate chiral compounds that could not be separated by any other immobilized chiral CSPs.

## Supporting information


**FIGURE S1** Structures of the selected test racemic compounds
**Figure S2** Representative separation chromatograms with CHIRALPAK IG and IK. The analytical conditions except eluent are shown in Table S1‐G and S1‐K
**Table S1‐A** The details of analytical conditions and chromatographic results of 67 compounds analysis with CHIRALPAK IA
**Table S1‐B** The details of analytical conditions and chromatographic results of 67 compounds analysis with CHIRALPAK IB
**Table S1‐C** The details of analytical conditions and chromatographic results of 67 compounds analysis with CHIRALPAK IC
**Table S1‐D** The details of analytical conditions and chromatographic results of 67 compounds analysis with CHIRALPAK ID
**Table S1‐E** The details of analytical conditions and chromatographic results of 67 compounds analysis with CHIRALPAK IE
**Table S1‐F** The details of analytical conditions and chromatographic results of 67 compounds analysis with CHIRALPAK IF
**Table S1‐G** The details of analytical conditions and chromatographic results of 67 compounds analysis with CHIRALPAK IG
**Table S1‐K** The details of analytical conditions and chromatographic results of 67 compounds analysis with CHIRALPAK IK
**Table S2‐A** The details of analytical conditions and chromatographic results of 50 compounds analysis with CHIRALPAK AS‐H
**Table S2‐B** The details of analytical conditions and chromatographic results of 50 compounds analysis with CHIRALPAK IH
**Table S3** The results of separation of 8 kinds of β‐lactone or β‐lactam compounds on 9 kinds of CSPs.

## Data Availability

The authors confirm that the data supporting the findings of this study are available within the article or its supporting information.
