# Supplementary material for: Characteristic and complementary chiral recognition ability of four recently developed immobilized chiral stationary phases based on amylose and cellulose phenyl carbamates and benzoates
Source: Chirality. 2022 Apr 12;34(7):925–40. doi: 10.1002/chir.23446 (PMC9321961; doi:10.1002/chir.23446)
Supplement: Supplementary file 1 — FIGURE S1 Structures of the selected test racemic compounds Figure S2 Representative separation chromatograms with CHIRALPAK IG and IK. The analytical conditions except eluent are shown in Table S1‐G and S1‐K Table S1‐A The details of analytical conditions and chromatographic results of 67 compounds analysis with CHIRALPAK IA Table S1‐B The details of analytical conditions and chromatographic results of 67 compounds analysis with CHIRALPAK IB Table S1‐C The details of analytical conditions and chromatographic results of 67 compounds analysis with CHIRALPAK IC Table S1‐D The details of analytical conditions and chromatographic results of 67 compounds analysis with CHIRALPAK ID Table S1‐E The details of analytical conditions and chromatographic results of 67 compounds analysis with CHIRALPAK IE Table S1‐F The details of analytical conditions and chromatographic results of 67 compounds analysis with CHIRALPAK IF Table S1‐G The details of analytical conditions and chromatographic results of 67 compounds analysis with CHIRALPAK IG Table S1‐K The details of analytical conditions and chromatographic results of 67 compounds analysis with CHIRALPAK IK Table S2‐A The details of analytical conditions and chromatographic results of 50 compounds analysis with CHIRALPAK AS‐H Table S2‐B The details of analytical conditions and chromatographic results of 50 compounds analysis with CHIRALPAK IH Table S3 The results of separation of 8 kinds of β‐lactone or β‐lactam compounds on 9 kinds of CSPs. [file CHIR-34-925-s001.docx]

**Supplementary information**

**Characteristic and complementary chiral recognition ability of 4 recently developed immobilized chiral stationary phases based on amylose and cellulose phenyl carbamates and benzoates**

Takafumi Onishi, Takunori Ueda, Kenichi Yoshida, Kosuke Uosaki, Hiroyuki Ando, Ryota Hamasaki and Atsushi Ohnishi.

DAICEL Corporation, CPI Company, Analytical Tools BU Research and Development Center,

Arai Factory, 1-1, Shinko-cho, Myoko, Niigata, 944-8550, Japan

Tel +81-255-72-9024

Fax +81-255-72-7892

* Corresponding author

E-mail address: tk_onishi@jp.daicel.com

**FIGURE S1** Structures of the selected test racemic compounds


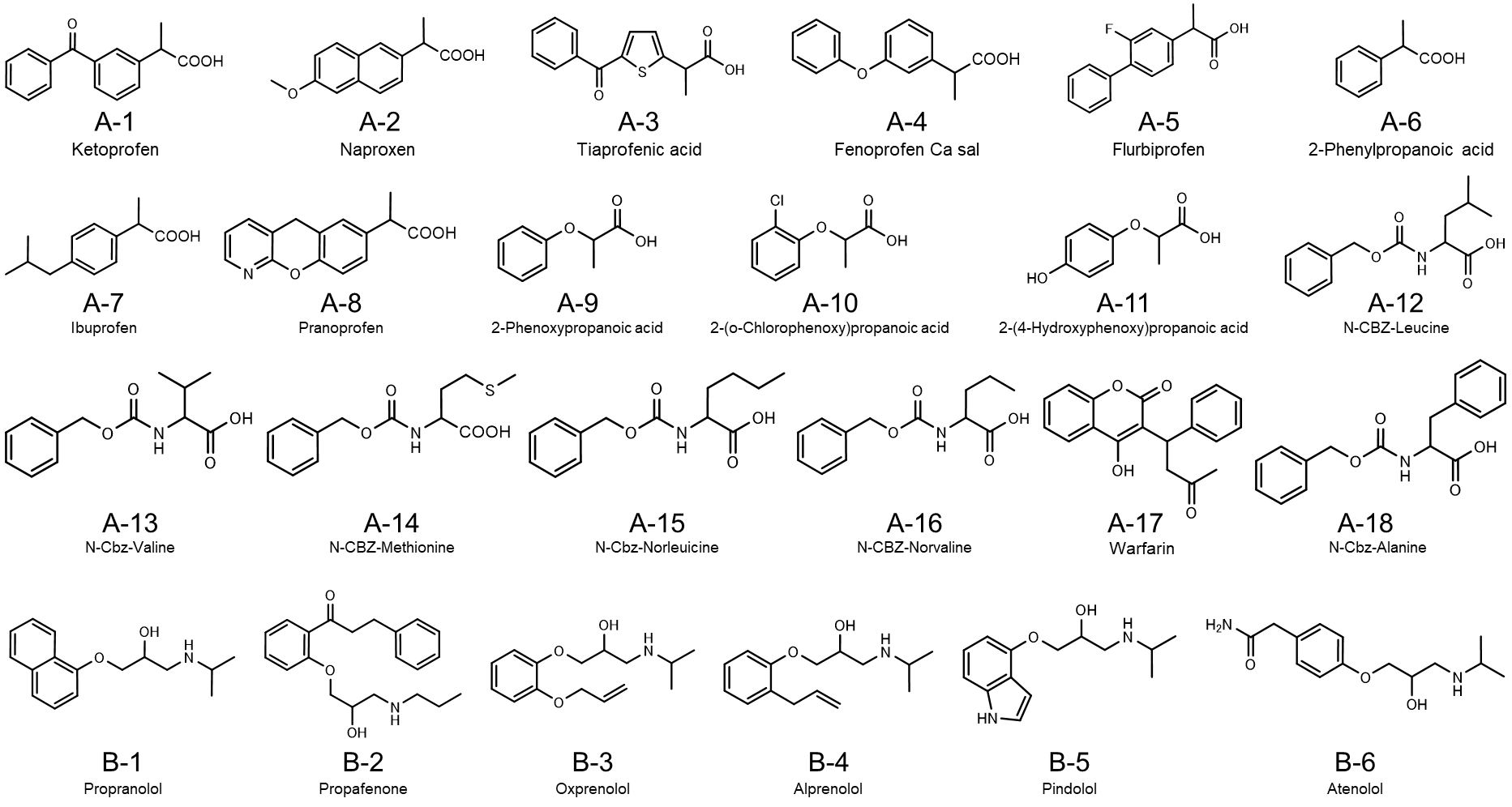


**FIGURE S1** (Continued)
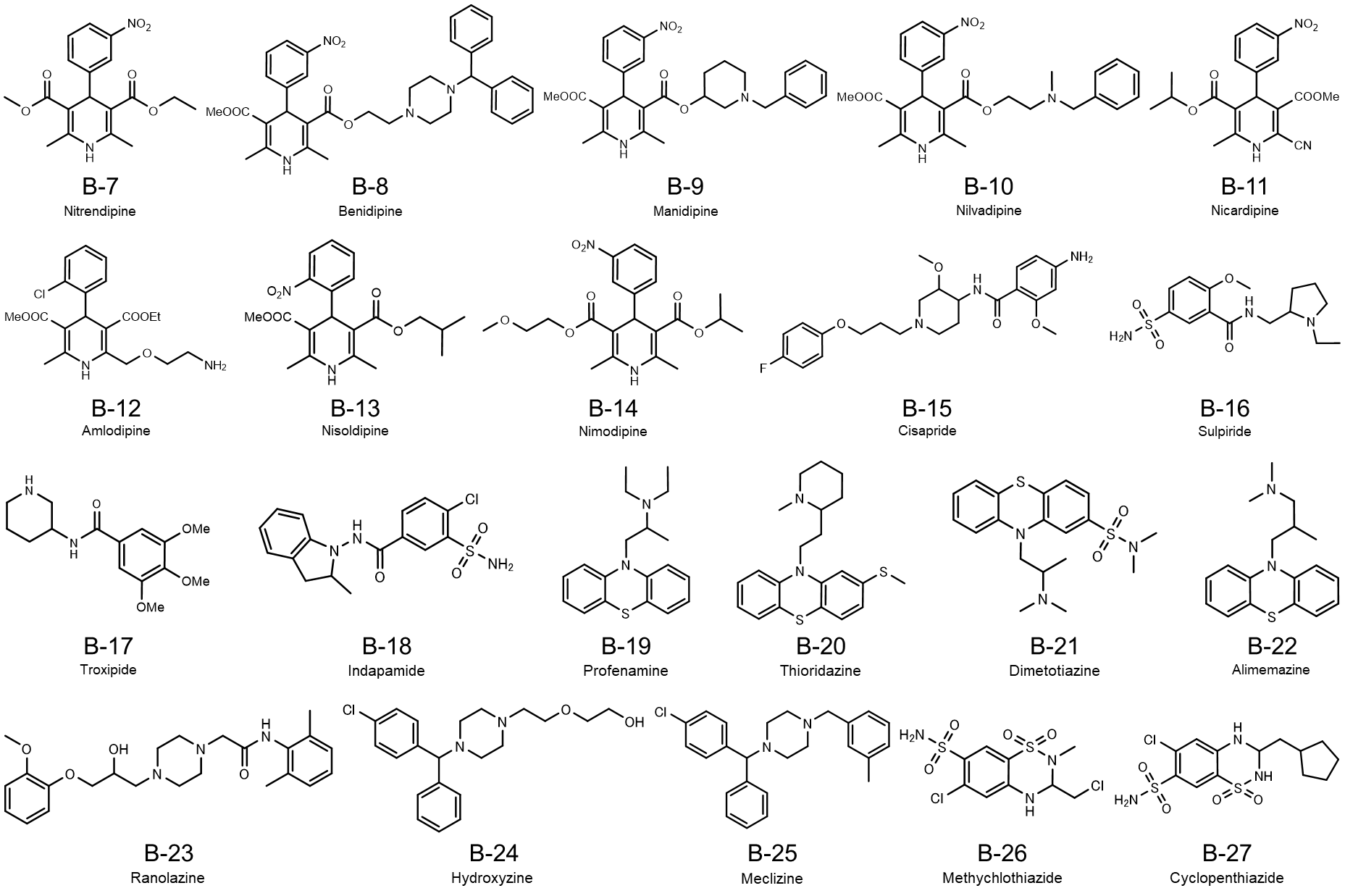


**FIGURE S1** (Continued)


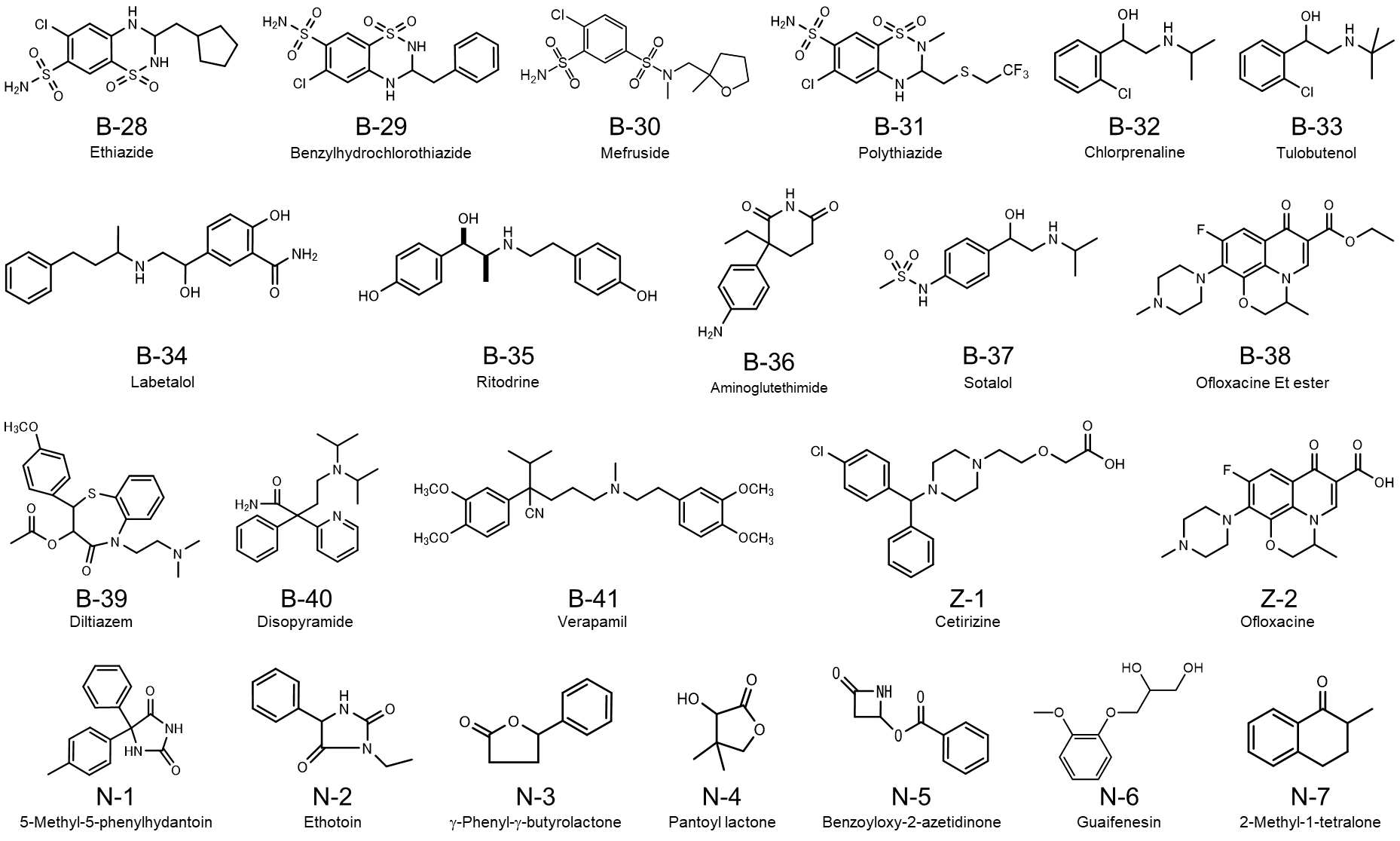


**FIGURE S1** (Continued)


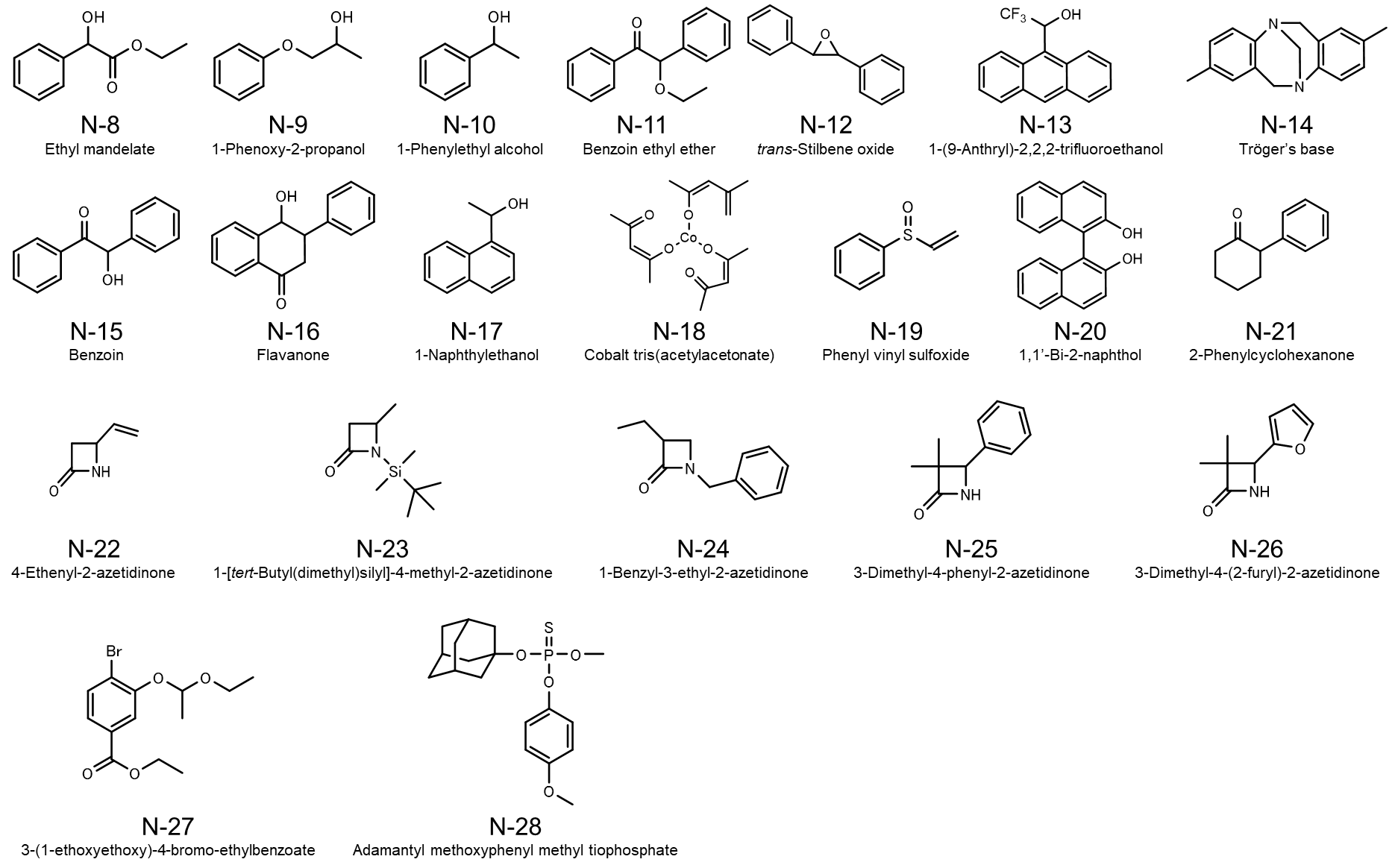


**TABLE S1-A** The details of analytical conditions and chromatographic results of 67 compounds analysis with CHIRALPAK IA

| Sample No. | Sample | Eluent*^a^* | Flow rate*^b^* | Detect*^b^* | Temp.*^b^* | t1 | t2 | k1 | k2 | *α* | *Rs* | Separation*^c^* |
| --- | --- | --- | --- | --- | --- | --- | --- | --- | --- | --- | --- | --- |
| A-1 | Ketoprofen | H/E/T=93/7/0.1 | 1.0 | 254 | 25 | 11.933 | 13.650 | 2.98 | 3.55 | 1.19 | 3.61 | A |
| A-2 | Naproxen | H/I/T=90/10/0.1 | 1.0 | 254 | 25 | 9.130 | 9.590 | 2.04 | 2.20 | 1.08 | 1.52 | C |
| A-3 | Tiaprofenic acid | H/I/T=90/10/0.1 | 1.0 | 254 | 25 | 12.600 | 14.130 | 3.20 | 3.71 | 1.16 | 3.35 | A |
| A-4 | Fenoprofen | H/I/T=90/10/0.1 | 1.0 | 254 | 25 | 6.130 | 6.960 | 1.04 | 1.32 | 1.27 | 3.94 | A |
| A-5 | Flurbiprofen | H/E/T=98/2/0.1 | 1.0 | 254 | 25 | 13.800 | 18.467 | 3.60 | 5.16 | 1.43 | 8.69 | A |
| A-6 | 2-Phenylpropanoic acid | H/I/T=98/2/0.1 | 1.0 | 230 | 25 | 14.230 | 15.030 | 3.74 | 4.01 | 1.07 | 1.83 | B |
| A-7 | Ibuprofen | H/I/T=98/2/0.1 | 1.0 | 230 | 25 | 12.250 | 12.620 | 3.08 | 3.21 | 1.04 | 0.91 | C |
| A-8 | Pranoprofen | H/E/T=80/20/0.1 | 1.0 | 230 | 25 | 10.400 | 12.058 | 2.47 | 3.02 | 1.22 | 3.46 | A |
| A-9 | 2-Phenoxypropanoic acid | H/I/T=90/10/0.1 | 1.0 | 230 | 25 | 4.780 | 5.620 | 0.59 | 0.87 | 1.47 | 4.12 | A |
| A-10 | 2-(*o*-Chlorophenoxy)propanoic acid | H/I/T=90/10/0.1 | 1.0 | 230 | 25 | 4.620 | 5.180 | 0.54 | 0.73 | 1.35 | 3.43 | A |
| A-11 | 2-(4-Hydroxyphenoxy)propanoic acid | H/E/T=93/7/0.1 | 1.0 | 254 | 25 | 12.133 | 13.492 | 3.04 | 3.50 | 1.15 | 2.71 | A |
| A-12 | N-Cbz-Leucine | H/I/T=50/50/0.1 | 1.0 | 230 | 25 | 4.082 | 4.957 | 0.36 | 0.65 | 1.81 | 5.02 | A |
| A-13 | N-Cbz-Valine | H/I/T=50/50/0.1 | 1.0 | 230 | 25 | 4.233 | 8.880 | 0.41 | 1.96 | 4.77 | 16.47 | A |
| A-14 | N-Cbz-Methionine | H/I/T=80/20/0.1 | 1.0 | 230 | 25 | 8.630 | 11.010 | 1.88 | 2.67 | 1.42 | 5.69 | A |
| A-15 | N-Cbz-Norleuicine | H/I/T=80/20/0.1 | 1.0 | 230 | 25 | 6.660 | 9.090 | 1.22 | 2.03 | 1.66 | 7.19 | A |
| A-16 | N-Cbz-Norvaline | H/I/T=80/20/0.1 | 1.0 | 230 | 25 | 6.770 | 10.570 | 1.26 | 2.52 | 2.01 | 9.75 | A |
| B-1 | Propranolol | H/E/D=93/7/0.1 | 1.0 | 230 | 25 | 6.858 | 8.067 | 1.29 | 1.69 | 1.31 | 4.11 | A |
| B-2 | Propafenone | H/E/D=90/10/0.1 | 1.0 | 230 | 25 | 12.792 | 20.742 | 3.26 | 5.91 | 1.81 | 10.94 | A |
| B-3 | Oxprenolol | H/E/D=93/7/0.1 | 1.0 | 230 | 25 | 4.833 | 7.250 | 0.61 | 1.42 | 2.32 | 8.96 | A |
| B-4 | Alprenolol | H/I/D=90/10/0.1 | 1.0 | 230 | 25 | 4.860 | 6.060 | 0.62 | 1.02 | 1.65 | 4.45 | A |
| B-5 | Pindolol | H/E/D=90/10/0.1 | 1.0 | 230 | 25 | 13.958 | 16.083 | 3.65 | 4.36 | 1.19 | 3.37 | A |
| B-6 | Atenolol | H/E/D=80/20/0.1 | 1.0 | 230 | 25 | 11.892 | - | 2.96 | - | 1.00 | - | D |
| B-7 | Nitrendipine | H/E/D=93/7/0.1 | 1.0 | 254 | 40 | 8.183 | 8.342 | 1.73 | 1.78 | 1.03 | - | C |
| B-8 | Manidipine | H/I/D=90/10/0.1 | 1.0 | 254 | 40 | 13.908 | 15.633 | 3.64 | 4.21 | 1.16 | 2.96 | A |
| B-9 | Benidipine | H/I/D=90/10/0.1 | 1.0 | 230 | 40 | 7.883 | 8.433 | 1.63 | 1.81 | 1.11 | 1.70 | B |
| B-10 | Nilvadipine | H/I/D=90/10/0.1 | 1.0 | 254 | 40 | 7.500 | 8.083 | 1.50 | 1.69 | 1.13 | 2.00 | A |
| B-11 | Nicardipine | H/E/D=93/7/0.1 | 1.0 | 254 | 40 | 9.708 | 10.067 | 2.24 | 2.36 | 1.05 | 1.07 | C |
| B-12 | Amlodipine | H/E/MEA=93/7/0.1 | 1.0 | 254 | 40 | 8.808 | 9.592 | 1.94 | 2.20 | 1.13 | 1.62 | C |
| B-13 | Nisoldipine | H/I/D=90/10/0.1 | 1.0 | 254 | 40 | 7.242 | - | 1.41 | - | 1.00 | - | D |
| B-14 | Nimodipine | H/I/D=90/10/0.1 | 1.0 | 254 | 40 | 7.958 | 8.242 | 1.65 | 1.75 | 1.06 | 0.77 | C |
| B-15 | Cisapride | H/I/D=80/20/0.1 | 1.0 | 254 | 25 | 13.108 | 14.958 | 3.37 | 3.99 | 1.18 | 2.35 | A |
| B-16 | Sulpiride | H/E/D=80/20/0.1 | 1.0 | 254 | 25 | 21.450 | 24.083 | 6.15 | 7.03 | 1.14 | 2.23 | A |
| B-17 | Troxipide | H/I/D=60/40/0.1 | 1.0 | 230 | 25 | 6.800 | - | 1.27 | - | 1.00 | 0.00 | D |
| B-18 | Indapamide | H/E/D=70/30/0.1 | 1.0 | 230 | 25 | 8.400 | 9.125 | 1.80 | 2.04 | 1.13 | 1.66 | B |
| B-19 | Profenamine | H/I/D=100/0.1/0.1 | 1.0 | 254 | 25 | 5.880 | 5.980 | 0.96 | 0.99 | 1.03 | 0.44 | C |

**TABLE S1-A** (Continued)

| Sample No. | Sample | Eluent*^a^* | Flow rate*^b^* | Detect*^b^* | Temp.*^b^* | t1 | t2 | k1 | k2 | *α* | *Rs* | Separation*^c^* |
| --- | --- | --- | --- | --- | --- | --- | --- | --- | --- | --- | --- | --- |
| B-20 | Thioridazine | H/E/D=100/1/0.1 | 1.0 | 254 | 25 | 10.125 | 10.600 | 2.38 | 2.53 | 1.07 | 1.12 | C |
| B-21 | Dimetotiazine | H/I/D=80/20/0.1 | 1.0 | 254 | 25 | 6.650 | 7.420 | 1.22 | 1.47 | 1.21 | 2.46 | A |
| B-22 | Alimemazine | H/I/D=100/0.1/0.1 | 1.0 | 254 | 25 | 7.958 | - | 1.65 | - | 1.00 | - | D |
| N-1 | 5-Methyl-5-phenylhydantoin | H/E=70/30 | 1.0 | 220 | 25 | 7.383 | 11.042 | 1.46 | 2.68 | 1.83 | 6.97 | A |
| N-2 | Ethotoin | H/I=80/20 | 1.0 | 220 | 25 | 9.090 | 10.210 | 2.03 | 2.40 | 1.18 | 2.47 | A |
| N-3 | γ-Phenyl-γ-butyrolactone | H/E=90/10 | 1.0 | 220 | 25 | 12.992 | 15.983 | 3.33 | 4.33 | 1.30 | 6.44 | A |
| N-4 | Pantoyl lactone | H/I=90/10 | 1.0 | 220 | 25 | 8.708 | 11.308 | 1.90 | 2.77 | 1.46 | 6.24 | A |
| N-5 | 4-Benzoyloxy-2-azetidinone | H/E=80/20 | 1.0 | 220 | 25 | 14.500 | 17.733 | 3.83 | 4.91 | 1.28 | 4.35 | A |
| B-23 | Ranolazine | H/I/D=50/50/0.1 | 1.0 | 230 | 25 | 6.420 | 8.783 | 1.14 | 1.93 | 1.69 | 5.52 | A |
| Z-1 | Cetirizine | H/E/A/D=50/50/0.1/0.1 | 1.0 | 230 | 25 | 8.425 | 9.108 | 1.81 | 2.04 | 1.13 | 0.91 | C |
| B-24 | Hydroxyzine | H/E/D=90/10/0.1 | 1.0 | 230 | 25 | 11.603 | 17.033 | 2.87 | 4.68 | 1.63 | 9.56 | A |
| B-25 | Meclizine | H/I/D=95/5/0.1 | 1.0 | 254 | 25 | 4.675 | 6.975 | 0.56 | 1.33 | 2.37 | 10.53 | A |
| B-26 | Methychlothiazide | H/I/D=70/30/0.1 | 1.0 | 230 | 25 | 8.230 | 9.230 | 1.74 | 2.08 | 1.19 | 1.10 | C |
| B-27 | Cyclopenthiazide | H/E/D=80/20/0.1 | 1.0 | 230 | 25 | 15.250 | 17.975 | 4.08 | 4.99 | 1.22 | 2.99 | A |
| B-28 | Ethiazide | H/E/D=80/20/0.1 | 1.0 | 230 | 25 | 12.233 | 13.725 | 3.08 | 3.58 | 1.16 | 1.90 | B |
| B-29 | Benzylhydrochlorothiazide | H/E/D=80/20/0.1 | 1.0 | 230 | 25 | 17.900 | 24.483 | 4.97 | 7.16 | 1.44 | 4.47 | A |
| B-30 | Mefruside | MeOH | 1.0 | 230 | 40 | 4.961 | 6.661 | 10.83 | 14.63 | 1.35 | 4.57 | A |
| B-31 | Polythiazide | H/E/D=80/20/0.1 | 1.0 | 230 | 25 | 15.700 | 21.408 | 4.23 | 6.14 | 1.45 | 5.33 | A |
| B-32 | Chlorprenaline | H/I/D=98/2/0.1 | 1.0 | 254 | 25 | 10.958 | - | 2.65 | - | 1.00 | - | D |
| B-33 | Tulobutenol | H/E/D=400/1/0.4 | 1.0 | 254 | 25 | 14.958 | 16.225 | 3.99 | 4.41 | 1.11 | 1.99 | B |
| B-34 | Labetalol | H/I/D=70/30/0.1 | 1.0 | 230 | 25 | 5.330 | 5.920 | 0.78 | 0.97 | 1.25 | - | C |
| B-35 | Ritodrine | H/E/D=90/10/0.1 | 1.0 | 275 | 25 | 16.549 | 18.048 | 4.18 | 4.65 | 1.11 | 1.04 | C |
| N-12 | *trans*-Stilbene oxide | H/I=90/10 | 1.0 | 254 | 25 | 4.528 | 6.087 | 0.55 | 1.08 | 1.97 | 11.04 | A |
| N-13 | 1-(9-Anthryl)-2,2,2-trifluoroethanol | H/I=90/10 | 1.0 | 254 | 25 | 7.330 | 14.120 | 1.51 | 3.83 | 2.54 | 17.73 | C |
| N-14 | Tröger’s base | H/I=90/10 | 1.0 | 254 | 25 | 5.568 | 5.976 | 0.91 | 1.05 | 1.15 | 1.82 | A |
| N-15 | Benzoin | H/I=90/10 | 1.0 | 254 | 25 | 8.735 | 10.929 | 1.99 | 2.74 | 1.38 | 7.32 | A |
| N-16 | Flavanone | H/I=90/10 | 1.0 | 254 | 25 | 6.232 | 7.173 | 1.13 | 1.46 | 1.28 | 5.33 | C |
| N-17 | 1-Naphthylethanol | H/I=90/10 | 1.0 | 254 | 25 | 7.074 | 9.257 | 1.42 | 2.17 | 1.53 | 8.87 | D |
| N-18 | Cobalt tris(acetylacetonate) | H/I=90/10 | 1.0 | 254 | 25 | 12.744 | - | 3.36 | - | 1.00 | - | D |
| N-19 | Phenyl vinyl sulfoxide | H/I=90/10 | 1.0 | 254 | 25 | 10.542 | 11.355 | 2.61 | 2.88 | 1.11 | 2.09 | D |
| N-20 | 1,1’-Bi-2-naphthol | H/I=90/10 | 1.0 | 254 | 25 | 12.904 | 14.322 | 3.42 | 3.90 | 1.14 | 1.51 | C |
| N-21 | 2-Phenylcyclohexanone | H/I=90/10 | 1.0 | 254 | 25 | 5.834 | 6.186 | 1.00 | 1.12 | 1.12 | 2.26 | C |

*^a^* “H” means *n*-hexane, “E” means ethanol, “I” means 2-propanol, “T” means trifluoroacetic acid, “D” means diethylamine and “MEA” means 2-aminoethanol.

*^b^* Flow rate’s unit is “mL/min”, Detect’s unit is “nm UV” and Temp.’s unit is “℃”.

*^c^* “A” means baseline separation, “B” means nearly baseline separation, “C” means partial separation and “D” means no separation.

**TABLE S1-B** The details of analytical conditions and chromatographic results of 67 compounds analysis with CHIRALPAK IB

| Sample No. | Sample | Eluent*^a^* | Flow rate*^b^* | Detect*^b^* | Temp.*^b^* | t1 | t2 | k1 | k2 | *α* | *Rs* | Separation*^c^* |
| --- | --- | --- | --- | --- | --- | --- | --- | --- | --- | --- | --- | --- |
| A-1 | Ketoprofen | H/E/T=93/7/0.1 | 1.0 | 254 | 25 | 7.100 | - | 1.37 | - | 1.00 | - | D |
| A-2 | Naproxen | H/E/T=98/2/0.1 | 1.0 | 230 | 25 | 14.242 | 15.900 | 3.75 | 4.30 | 1.15 | 3.44 | A |
| A-3 | Tiaprofenic acid | H/I/T=90/10/0.1 | 1.0 | 254 | 25 | 8.140 | 8.870 | 1.71 | 1.96 | 1.14 | 2.15 | A |
| A-4 | Fenoprofen | H/I/T=98/2/0.1 | 1.0 | 254 | 25 | 11.610 | 12.230 | 2.87 | 3.08 | 1.07 | 1.55 | C |
| A-5 | Flurbiprofen | H/I/T=98/2/0.1 | 1.0 | 254 | 25 | 10.230 | 10.840 | 2.41 | 2.61 | 1.08 | 1.59 | C |
| A-6 | 2-Phenylpropanoic acid | H/I/T=98/2/0.1 | 1.0 | 230 | 25 | 8.670 | 9.550 | 1.89 | 2.18 | 1.16 | 3.12 | A |
| A-7 | Ibuprofen | H/I/T=98/2/0.1 | 1.0 | 230 | 25 | 6.720 | 7.150 | 1.24 | 1.38 | 1.12 | 1.95 | B |
| A-8 | Pranoprofen | H/I/T=90/10/0.1 | 1.0 | 230 | 25 | 20.260 | - | 5.75 | - | 1.00 | - | D |
| A-9 | 2-Phenoxypropanoic acid | H/E/T=98/2/0.1 | 1.0 | 230 | 25 | 9.283 | 15.383 | 2.09 | 4.13 | 1.97 | 15.91 | A |
| A-10 | 2-(*o*-Chlorophenoxy)propanoic acid | H/I/T=90/10/0.1 | 1.0 | 230 | 25 | 4.500 | 4.970 | 0.50 | 0.66 | 1.31 | 2.93 | A |
| A-11 | 2-(4-Hydroxyphenoxy)propanoic acid | H/E/T=93/7/0.1 | 1.0 | 254 | 25 | 12.333 | 13.500 | 3.11 | 3.50 | 1.13 | 2.22 | A |
| A-12 | N-Cbz-Leucine | H/E/T=90/10/0.1 | 1.0 | 230 | 25 | 5.925 | 6.908 | 0.98 | 1.30 | 1.34 | 3.89 | A |
| A-13 | N-Cbz-Valine | H/E/T=90/10/0.1 | 1.0 | 230 | 25 | 5.542 | 7.775 | 0.85 | 1.59 | 1.88 | 8.10 | A |
| A-14 | N-Cbz-Methionine | H/I/T=80/20/0.1 | 1.0 | 230 | 25 | 5.810 | 6.800 | 0.94 | 1.27 | 1.35 | 3.56 | A |
| A-15 | N-Cbz-Norleuicine | H/I/T=80/20/0.1 | 1.0 | 230 | 25 | 4.640 | 5.430 | 0.55 | 0.81 | 1.48 | 3.65 | A |
| A-16 | N-Cbz-Norvaline | H/I/T=80/20/0.1 | 1.0 | 230 | 25 | 4.740 | 6.410 | 0.58 | 1.14 | 1.96 | 7.02 | A |
| B-1 | Propranolol | H/I/EDA=80/20/0.1 | 1.0 | 230 | 25 | 5.542 | 7.108 | 0.85 | 1.37 | 1.62 | 5.03 | A |
| B-2 | Propafenone | H/I/EDA=90/10/0.1 | 1.0 | 230 | 25 | 8.683 | 8.967 | 1.89 | 1.99 | 1.05 | - | C |
| B-3 | Oxprenolol | H/I/EDA=80/20/0.1 | 1.0 | 230 | 25 | 4.883 | 10.517 | 0.63 | 2.51 | 3.99 | 17.33 | A |
| B-4 | Alprenolol | H/I/EDA=90/10/0.1 | 1.0 | 230 | 25 | 4.408 | 6.075 | 0.47 | 1.03 | 2.18 | 7.50 | A |
| B-5 | Pindolol | H/I/EDA=70/30/0.1 | 1.0 | 230 | 25 | 6.925 | 21.875 | 1.31 | 6.29 | 4.81 | 17.85 | A |
| B-6 | Atenolol | H/I/EDA=80/20/0.1 | 1.0 | 230 | 25 | 16.760 | 21.430 | 4.59 | 6.14 | 1.34 | 2.79 | A |
| B-7 | Nitrendipine | H/E/D=93/7/0.1 | 1.0 | 254 | 40 | 7.775 | - | 1.59 | - | 1.00 | - | D |
| B-8 | Manidipine | H/E/D=93/7/0.1 | 1.0 | 254 | 40 | 10.942 | 11.267 | 2.65 | 2.76 | 1.04 | - | C |
| B-9 | Benidipine | H/I/D=90/10/0.1 | 1.0 | 254 | 40 | 9.958 | 10.842 | 2.32 | 2.61 | 1.13 | 1.74 | B |
| B-10 | Nilvadipine | H/I/D=90/10/0.1 | 1.0 | 254 | 40 | 7.800 | 8.142 | 1.60 | 1.71 | 1.07 | 0.91 | C |
| B-11 | Nicardipine | H/I/D=90/10/0.1 | 1.0 | 254 | 40 | 10.075 | 10.383 | 2.36 | 2.46 | 1.04 | - | C |
| B-12 | Amlodipine | H/E/MEA=93/7/0.1 | 1.0 | 254 | 40 | 10.825 | - | 2.61 | - | 1.00 | - | D |
| B-13 | Nisoldipine | H/I/D=90/10/0.1 | 1.0 | 254 | 40 | 8.717 | - | 1.91 | - | 1.00 | - | D |
| B-14 | Nimodipine | H/I/D=90/10/0.1 | 1.0 | 254 | 40 | 8.825 | 9.058 | 1.94 | 2.02 | 1.04 | - | C |
| B-15 | Cisapride | H/E/D=80/20/0.1 | 1.0 | 254 | 25 | 9.575 | 10.000 | 2.19 | 2.33 | 1.06 | 0.88 | C |
| B-16 | Sulpiride | H/E/D=70/30/0.1 | 1.0 | 254 | 25 | 12.025 | - | 3.01 | - | 1.00 | - | D |
| B-17 | Troxipide | H/E/D=60/40/0.1 | 1.0 | 230 | 25 | 6.317 | 8.108 | 1.11 | 1.70 | 1.54 | 2.43 | A |
| B-18 | Indapamide | H/E/D=50/50/0.1 | 1.0 | 230 | 25 | 5.517 | 7.425 | 0.84 | 1.48 | 1.76 | 5.27 | A |
| B-19 | Profenamine | H/E/D=100/0.1/0.1 | 1.0 | 254 | 25 | 5.867 | 6.133 | 0.96 | 1.04 | 1.09 | 1.09 | C |

**TABLE S1-B** (Continued)

| Sample No. | Sample | Eluent*^a^* | Flow rate*^b^* | Detect*^b^* | Temp.*^b^* | t1 | t2 | k1 | k2 | *α* | *Rs* | Separation*^c^* |
| --- | --- | --- | --- | --- | --- | --- | --- | --- | --- | --- | --- | --- |
| B-20 | Thioridazine | H/E/D=100/1/0.1 | 1.0 | 254 | 25 | 7.600 | - | 1.53 | - | 1.00 | - | D |
| B-21 | Dimetotiazine | H/I/EDA=90/10/0.1 | 1.0 | 254 | 25 | 8.692 | - | 1.90 | - | 1.00 | - | D |
| B-22 | Alimemazine | H/I/D=300/1/0.3 | 1.0 | 254 | 25 | 5.967 | 6.142 | 0.99 | 1.05 | 1.06 | - | C |
| N-1 | 5-Methyl-5-phenylhydantoin | H/I=80/20 | 1.0 | 220 | 25 | 6.210 | 6.410 | 1.07 | 1.14 | 1.06 | 0.55 | C |
| N-2 | Ethotoin | H/E=90/10 | 1.0 | 220 | 25 | 14.900 | 18.367 | 3.97 | 5.12 | 1.29 | 6.07 | A |
| N-3 | γ-Phenyl-γ-butyrolactone | H/E=90/10 | 1.0 | 220 | 25 | 9.314 | 9.435 | 2.10 | 2.15 | 1.02 | - | C |
| N-4 | Pantoyl lactone | H/E=90/10 | 1.0 | 220 | 25 | 7.675 | 7.917 | 1.56 | 1.64 | 1.05 | 0.74 | C |
| N-5 | 4-Benzoyloxy-2-azetidinone | H/I=90/10 | 1.0 | 220 | 25 | 11.575 | 13.300 | 2.86 | 3.43 | 1.20 | 3.44 | A |
| B-23 | Ranolazine | H/I/D=50/50/0.1 | 1.0 | 230 | 25 | 12.122 | 29.863 | 3.04 | 8.95 | 2.94 | 13.09 | A |
| Z-1 | Cetirizine | H/E/A/D=50/50/0.1/0.1 | 1.0 | 230 | 25 | 13.100 | - | 3.37 | - | 1.00 | - | D |
| B-24 | Hydroxyzine | H/I/D=80/20/0.1 | 1.0 | 230 | 25 | 5.300 | 5.450 | 0.77 | 0.82 | 1.07 | - | C |
| B-25 | Meclizine | H/I/D=95/5/0.1 | 1.0 | 254 | 25 | 3.950 | - | 0.32 | - | 1.00 | - | D |
| B-26 | Methychlothiazide | H/I/D=70/30/0.1 | 1.0 | 230 | 25 | 16.783 | - | 4.59 | - | 1.00 | - | D |
| B-27 | Cyclopenthiazide | H/E/D=80/20/0.1 | 1.0 | 230 | 25 | 13.267 | 15.167 | 3.42 | 4.06 | 1.19 | 1.72 | B |
| B-28 | Ethiazide | H/E/D=80/20/0.1 | 1.0 | 230 | 25 | 15.175 | 18.008 | 4.06 | 5.00 | 1.23 | 1.96 | B |
| B-29 | Benzylhydrochlorothiazide | H/E/D=80/20/0.1 | 1.0 | 230 | 25 | 22.042 | 24.792 | 6.35 | 7.26 | 1.14 | 1.22 | C |
| B-30 | Mefruside | H/I/EDA=70/30/0.1 | 1.0 | 230 | 25 | 15.142 | - | 4.05 | - | 1.00 | - | D |
| B-31 | Polythiazide | H/I/D=70/30/0.1 | 1.0 | 230 | 25 | 16.275 | - | 4.43 | - | 1.00 | - | D |
| B-32 | Chlorprenaline | H/I/D=98/2/0.1 | 1.0 | 254 | 25 | 7.500 | - | 1.50 | - | 1.00 | - | D |
| B-33 | Tulobutenol | H/E/D=400/1/0.4 | 1.0 | 254 | 25 | 12.250 | - | 3.08 | - | 1.00 | - | D |
| B-34 | Labetalol | H/E/D=90/10/0.1 | 1.0 | 230 | 25 | 16.167 | - | 4.39 | - | 1.00 | - | D |
| B-35 | Ritodrine | H/I/D=80/20/0.1 | 1.0 | 275 | 25 | 12.158 | - | 3.05 | - | 1.00 | - | D |
| N-12 | *trans*-Stilbene oxide | H/I=90/10 | 1.0 | 254 | 25 | 4.528 | 6.087 | 0.55 | 1.08 | 1.97 | 11.04 | A |
| N-13 | 1-(9-Anthryl)-2,2,2-trifluoroethanol | H/I=90/10 | 1.0 | 254 | 25 | 7.330 | 14.120 | 1.51 | 3.83 | 2.54 | 17.73 | A |
| N-14 | Tröger’s base | H/I=90/10 | 1.0 | 254 | 25 | 5.568 | 5.976 | 0.91 | 1.05 | 1.15 | 1.82 | B |
| N-15 | Benzoin | H/I=90/10 | 1.0 | 254 | 25 | 8.735 | 10.929 | 1.99 | 2.74 | 1.38 | 7.32 | A |
| N-16 | Flavanone | H/I=90/10 | 1.0 | 254 | 25 | 6.232 | 7.173 | 1.13 | 1.46 | 1.28 | 5.33 | A |
| N-17 | 1-Naphthylethanol | H/I=90/10 | 1.0 | 254 | 25 | 7.074 | 9.257 | 1.42 | 2.17 | 1.53 | 8.87 | A |
| N-18 | Cobalt tris(acetylacetonate) | H/I=90/10 | 1.0 | 254 | 25 | 12.744 | - | 3.36 | - | 1.00 | - | D |
| N-19 | Phenyl vinyl sulfoxide | H/I=90/10 | 1.0 | 254 | 25 | 10.542 | 11.355 | 2.61 | 2.88 | 1.11 | 2.09 | A |
| N-20 | 1,1’-Bi-2-naphthol | H/I=90/10 | 1.0 | 254 | 25 | 12.904 | 14.322 | 3.42 | 3.90 | 1.14 | 1.51 | B |
| N-21 | 2-Phenylcyclohexanone | H/I=90/10 | 1.0 | 254 | 25 | 5.834 | 6.186 | 1.00 | 1.12 | 1.12 | 2.26 | A |

*^a^* “H” means *n*-hexane, “E” means ethanol, “I” means 2-propanol, “T” means trifluoroacetic acid, “D” means diethylamine and “MEA” means 2-aminoethanol.

*^b^* Flow rate’s unit is “mL/min”, Detect’s unit is “nm UV” and Temp.’s unit is “℃”.

*^c^* “A” means baseline separation, “B” means nearly baseline separation, “C” means partial separation and “D” means no separation.

**TABLE S1-C** The details of analytical conditions and chromatographic results of 67 compounds analysis with CHIRALPAK IC

| Sample No. | Sample | Eluent*^a^* | Flow rate*^b^* | Detect*^b^* | Temp.*^b^* | t1 | t2 | k1 | k2 | *α* | *Rs* | Separation*^c^* |
| --- | --- | --- | --- | --- | --- | --- | --- | --- | --- | --- | --- | --- |
| A-1 | Ketoprofen | H/E/T=93/7/0.1 | 1.0 | 254 | 25 | 11.930 | 13.650 | 2.84 | 3.28 | 1.19 | 3.61 | A |
| A-2 | Naproxen | H/E/T=98/2/0.1 | 1.0 | 254 | 25 | 16.008 | 18.108 | 4.34 | 5.04 | 1.16 | 3.43 | A |
| A-3 | Tiaprofenic acid | H/E/T=93/7/0.1 | 1.0 | 254 | 25 | 19.308 | 20.358 | 5.44 | 5.79 | 1.06 | 1.41 | C |
| A-4 | Fenoprofen | H/I/T=98/2/0.1 | 1.0 | 254 | 25 | 11.180 | 12.670 | 2.73 | 3.22 | 1.18 | 2.92 | A |
| A-5 | Flurbiprofen | H/E/T=98/2/0.1 | 1.0 | 254 | 25 | 7.367 | 7.492 | 1.46 | 1.50 | 1.03 | - | C |
| A-6 | 2-Phenylpropanoic acid | H/I/T=98/2/0.1 | 1.0 | 230 | 25 | 10.050 | - | 2.35 | - | 1.00 | - | D |
| A-7 | Ibuprofen | H/I/T=98/2/0.1 | 1.0 | 230 | 25 | 8.610 | 8.900 | 1.87 | 1.97 | 1.05 | 0.68 | C |
| A-8 | Pranoprofen | H/E/T=80/20/0.1 | 1.0 | 230 | 25 | 10.234 | 10.363 | 2.41 | 2.45 | 1.02 | 0.04 | C |
| A-9 | 2-Phenoxypropanoic acid | H/I/T=98/2/0.1 | 1.0 | 230 | 25 | 11.050 | 12.930 | 2.68 | 3.31 | 1.23 | 1.97 | B |
| A-10 | 2-(*o*-Chlorophenoxy)propanoic acid | H/I/T=98/2/0.1 | 1.0 | 230 | 25 | 9.210 | 10.470 | 2.07 | 2.49 | 1.20 | 2.82 | A |
| A-11 | 2-(4-Hydroxyphenoxy)propanoic acid | H/I/T=90/10/0.1 | 1.0 | 230 | 25 | 8.970 | 11.360 | 1.99 | 2.79 | 1.40 | 5.08 | A |
| A-12 | N-Cbz-Leucine | H/E/T=90/10/0.1 | 1.0 | 230 | 25 | 7.258 | - | 1.42 | - | 1.00 | - | D |
| A-13 | N-Cbz-Valine | H/E/T=90/10/0.1 | 1.0 | 230 | 25 | 6.675 | 7.542 | 1.23 | 1.51 | 1.24 | 2.19 | A |
| A-14 | N-Cbz-Methionine | H/E/T=90/10/0.1 | 1.0 | 230 | 25 | 9.492 | 10.333 | 2.16 | 2.44 | 1.13 | 1.97 | B |
| A-15 | N-Cbz-Norleuicine | H/E/T=90/10/0.1 | 1.0 | 230 | 25 | 7.275 | 7.883 | 1.43 | 1.63 | 1.14 | 1.85 | C |
| A-16 | N-Cbz-Norvaline | H/E/T=90/10/0.1 | 1.0 | 230 | 25 | 7.642 | 8.225 | 1.55 | 1.74 | 1.13 | 1.38 | C |
| B-1 | Propranolol | H/I/D=80/20/0.1 | 1.0 | 230 | 25 | 5.217 | 6.692 | 0.74 | 1.23 | 1.67 | 3.92 | A |
| B-2 | Propafenone | H/I/D=90/10/0.1 | 1.0 | 230 | 25 | 20.508 | 21.817 | 5.84 | 6.27 | 1.07 | 1.22 | C |
| B-3 | Oxprenolol | H/I/D=80/20/0.1 | 1.0 | 230 | 25 | 6.108 | 9.033 | 1.04 | 2.01 | 1.94 | 7.99 | A |
| B-4 | Alprenolol | H/I/D=98/2/0.1 | 1.0 | 230 | 25 | 10.883 | 12.342 | 2.63 | 3.11 | 1.19 | 2.44 | A |
| B-5 | Pindolol | H/I/D=70/30/0.1 | 1.0 | 230 | 25 | 5.917 | 8.758 | 0.97 | 1.92 | 1.97 | 6.76 | A |
| B-6 | Atenolol | H/I/D=70/30/0.2 | 1.0 | 230 | 25 | 22.230 | 23.712 | 6.41 | 6.90 | 1.08 | 1.26 | C |
| B-7 | Nitrendipine | H/E/D=93/7/0.1 | 1.0 | 254 | 40 | 10.442 | - | 2.48 | - | 1.00 | - | D |
| B-8 | Manidipine | H/E/D=93/7/0.1 | 1.0 | 254 | 40 | 14.717 | 15.000 | 3.91 | 4.00 | 1.02 | - | C |
| B-9 | Benidipine | H/I/D=90/10/0.1 | 1.0 | 254 | 40 | 11.608 | 12.700 | 2.87 | 3.23 | 1.13 | 1.73 | B |
| B-10 | Nilvadipine | H/I/D=90/10/0.1 | 1.0 | 254 | 40 | 15.292 | 18.167 | 4.10 | 5.06 | 1.23 | 3.67 | A |
| B-11 | Nicardipine | H/I/D=90/10/0.1 | 1.0 | 254 | 40 | 13.000 | 14.192 | 3.33 | 3.73 | 1.12 | 1.92 | B |
| B-12 | Amlodipine | H/I/MEA=80/20/0.1 | 1.0 | 254 | 40 | 20.358 | 21.375 | 5.79 | 6.13 | 1.06 | 1.08 | C |
| B-13 | Nisoldipine | H/I/D=90/10/0.1 | 1.0 | 254 | 40 | 16.358 | 18.167 | 4.45 | 5.06 | 1.14 | 1.98 | B |
| B-14 | Nimodipine | H/I/D=90/10/0.1 | 1.0 | 254 | 40 | 12.967 | 21.017 | 3.32 | 6.01 | 1.81 | 9.96 | A |
| B-15 | Cisapride | H/I/EtA=70/30/0.1 | 1.0 | 254 | 25 | 15.833 | 17.250 | 4.28 | 4.75 | 1.11 | 1.08 | C |
| B-16 | Sulpiride | H/E/D=70/30/0.1 | 1.0 | 254 | 25 | 27.583 | 28.917 | 8.19 | 8.64 | 1.05 | 0.82 | C |
| B-17 | Troxipide | H/I/D=50/50/0.1 | 1.0 | 230 | 25 | 18.100 | 68.433 | 5.03 | 21.81 | 4.33 | 13.78 | A |
| B-18 | Indapamide | H/I/D=50/50/0.1 | 1.0 | 230 | 25 | 7.492 | 8.700 | 1.50 | 1.90 | 1.27 | 1.86 | B |
| B-19 | Profenamine | H/I/D=100/0.1/0.1 | 1.0 | 254 | 25 | 9.842 | 13.842 | 2.28 | 3.61 | 1.58 | 4.58 | A |

**TABLE S1-C** (Continued)

| Sample No. | Sample | Eluent*^a^* | Flow rate*^b^* | Detect*^b^* | Temp.*^b^* | t1 | t2 | k1 | k2 | *α* | *Rs* | Separation*^c^* |
| --- | --- | --- | --- | --- | --- | --- | --- | --- | --- | --- | --- | --- |
| B-20 | Thioridazine | H/I/D=98/2/0.1 | 1.0 | 254 | 25 | 9.208 | 9.492 | 2.07 | 2.16 | 1.05 | - | C |
| B-21 | Dimetotiazine | H/I/EtA=90/10/0.1 | 1.0 | 254 | 25 | 51.483 | 54.400 | 16.16 | 17.13 | 1.06 | 1.12 | C |
| B-22 | Alimemazine | H/I/D=100/0.1/0.1 | 1.0 | 254 | 25 | 17.875 | 21.225 | 4.96 | 6.08 | 1.23 | 1.83 | B |
| N-1 | 5-Methyl-5-phenylhydantoin | H/I=80/20 | 1.0 | 220 | 25 | 6.160 | 6.360 | 1.05 | 1.12 | 1.06 | 0.62 | C |
| N-2 | Ethotoin | H/I=80/20 | 1.0 | 220 | 25 | 14.400 | 16.490 | 3.80 | 4.50 | 1.18 | 2.94 | A |
| N-3 | γ-Phenyl-γ-butyrolactone | H/E=90/10 | 1.0 | 220 | 25 | 16.700 | 17.108 | 4.57 | 4.70 | 1.03 | - | C |
| N-4 | Pantoyl lactone | H/I=90/10 | 1.0 | 220 | 25 | 22.833 | 27.308 | 6.61 | 8.10 | 1.23 | 4.40 | A |
| N-5 | 4-Benzoyloxy-2-azetidinone | H/I=70/30 | 1.0 | 220 | 25 | 10.708 | 21.217 | 2.57 | 6.07 | 2.36 | 15.18 | A |
| B-23 | Ranolazine | H/I/D=50/50/0.1 | 1.0 | 230 | 25 | 47.423 | 67.645 | 14.81 | 21.55 | 1.46 | 4.24 | A |
| Z-1 | Cetirizine | H/E/A/D=50/50/0.1/0.1 | 1.0 | 230 | 25 | 22.942 | 23.850 | 6.65 | 6.95 | 1.05 | - | C |
| B-24 | Hydroxyzine | H/I/D=80/20/0.1 | 1.0 | 230 | 25 | 7.225 | 7.508 | 1.41 | 1.50 | 1.07 | 0.79 | C |
| B-25 | Meclizine | H/I/D=95/5/0.1 | 1.0 | 254 | 25 | 3.800 | - | 0.27 | - | 1.00 | - | D |
| B-26 | Methychlothiazide | H/I/D=70/30/0.1 | 1.0 | 230 | 25 | 17.225 | 21.675 | 4.74 | 6.23 | 1.31 | 2.50 | A |
| B-27 | Cyclopenthiazide | H/E/D=80/20/0.1 | 1.0 | 230 | 25 | 10.008 | 12.583 | 2.34 | 3.19 | 1.37 | 3.71 | A |
| B-28 | Ethiazide | H/E/D=80/20/0.1 | 1.0 | 230 | 25 | 11.242 | 15.667 | 2.75 | 4.22 | 1.54 | 5.17 | A |
| B-29 | Benzylhydrochlorothiazide | H/E/D=80/20/0.1 | 1.0 | 230 | 25 | 12.708 | 18.792 | 3.24 | 5.26 | 1.63 | 5.34 | A |
| B-30 | Mefruside | H/I/D=70/30/0.1 | 1.0 | 230 | 25 | 17.608 | - | 4.87 | - | 1.00 | - | D |
| B-31 | Polythiazide | H/I/D=70/30/0.1 | 1.0 | 230 | 25 | 13.100 | 16.233 | 3.37 | 4.41 | 1.31 | 2.19 | A |
| B-32 | Chlorprenaline | H/I/D=98/2/0.1 | 1.0 | 254 | 25 | 7.775 | 10.658 | 1.59 | 2.55 | 1.60 | 8.28 | A |
| B-33 | Tulobutenol | H/I/D=98/2/0.1 | 1.0 | 254 | 25 | 6.783 | 9.233 | 1.26 | 2.08 | 1.65 | 8.37 | A |
| B-34 | Labetalol | H/E/D=90/10/0.1 | 1.0 | 230 | 25 | 14.150 | 15.342 | 3.72 | 4.11 | 1.11 | - | C |
| B-35 | Ritodrine | H/I/D=80/20/0.1 | 1.0 | 275 | 25 | 8.692 | 9.683 | 1.90 | 2.23 | 1.17 | 1.53 | B |
| N-12 | *trans*-Stilbene oxide | H/I=90/10 | 1.0 | 254 | 25 | 4.575 | 5.755 | 0.51 | 0.89 | 1.77 | 8.86 | A |
| N-13 | 1-(9-Anthryl)-2,2,2-trifluoroethanol | H/I=90/10 | 1.0 | 254 | 25 | 3.783 | 4.184 | 0.25 | 0.38 | 1.54 | 2.18 | A |
| N-14 | Tröger’s base | H/I=90/10 | 1.0 | 254 | 25 | 5.897 | 7.271 | 0.94 | 1.40 | 1.48 | 6.23 | A |
| N-15 | Benzoin | H/I=90/10 | 1.0 | 254 | 25 | 13.279 | 14.318 | 3.38 | 3.72 | 1.10 | 2.25 | A |
| N-16 | Flavanone | H/I=90/10 | 1.0 | 254 | 25 | 7.912 | 8.798 | 1.61 | 1.90 | 1.18 | 3.55 | A |
| N-17 | 1-Naphthylethanol | H/I=90/10 | 1.0 | 254 | 25 | 5.972 | 6.733 | 0.97 | 1.22 | 1.26 | 3.89 | A |
| N-18 | Cobalt tris(acetylacetonate) | H/I=90/10 | 1.0 | 254 | 25 | 5.748 | 8.013 | 0.89 | 1.64 | 1.84 | 7.15 | A |
| N-19 | Phenyl vinyl sulfoxide | H/I=90/10 | 1.0 | 254 | 25 | 36.744 | 37.605 | 11.10 | 11.39 | 1.03 | - | C |
| N-20 | 1,1’-Bi-2-naphthol | H/I=90/10 | 1.0 | 254 | 25 | 7.265 | 9.346 | 1.39 | 2.08 | 1.49 | 5.42 | A |
| N-21 | 2-Phenylcyclohexanone | H/I=90/10 | 1.0 | 254 | 25 | 11.269 | 13.230 | 2.71 | 3.36 | 1.24 | 3.51 | A |

*^a^* “H” means *n*-hexane, “E” means ethanol, “I” means 2-propanol, “T” means trifluoroacetic acid, “D” means diethylamine and “MEA” means 2-aminoethanol.

*^b^* Flow rate’s unit is “mL/min”, Detect’s unit is “nm UV” and Temp.’s unit is “℃”.

*^c^* “A” means baseline separation, “B” means nearly baseline separation, “C” means partial separation and “D” means no separation.

**TABLE S1-D** The details of analytical conditions and chromatographic results of 67 compounds analysis with CHIRALPAK ID

| Sample No. | Sample | Eluent*^a^* | Flow rate*^b^* | Detect*^b^* | Temp.*^b^* | t1 | t2 | k1 | k2 | *α* | *Rs* | Separation*^c^* |
| --- | --- | --- | --- | --- | --- | --- | --- | --- | --- | --- | --- | --- |
| A-1 | Ketoprofen | H/E/T=93/7/0.1 | 1.0 | 230 | 25 | 14.625 | 16.917 | 3.88 | 4.64 | 1.20 | 4.08 | A |
| A-2 | Naproxen | H/E/T=93/7/0.1 | 1.0 | 254 | 25 | 7.733 | 8.117 | 1.58 | 1.71 | 1.08 | 1.41 | C |
| A-3 | Tiaprofenic acid | H/E/T=93/7/0.1 | 1.0 | 230 | 25 | 25.283 | 26.975 | 7.43 | 7.99 | 1.08 | 1.75 | B |
| A-4 | Fenoprofen | H/I/T=98/2/0.1 | 1.0 | 254 | 25 | 12.142 | - | 3.05 | - | 1.00 | - | D |
| A-5 | Flurbiprofen | H/E/T=98/2/0.1 | 1.0 | 254 | 25 | 8.792 | 10.708 | 1.93 | 2.57 | 1.33 | 4.86 | A |
| A-6 | 2-Phenylpropanoic acid | H/E/T=98/2/0.1 | 1.0 | 230 | 25 | 7.142 | 7.383 | 1.38 | 1.46 | 1.06 | 1.11 | C |
| A-7 | Ibuprofen | H/I/T=98/2/0.1 | 1.0 | 230 | 25 | 7.558 | 7.800 | 1.52 | 1.60 | 1.05 | - | C |
| A-8 | Pranoprofen | H/I/T=70/30/0.1 | 1.0 | 230 | 25 | 9.056 | 9.931 | 2.02 | 2.31 | 1.14 | 2.58 | A |
| A-9 | 2-Phenoxypropanoic acid | H/I/T=90/10/0.1 | 1.0 | 230 | 25 | 4.333 | 5.025 | 0.44 | 0.68 | 1.52 | 3.49 | A |
| A-10 | 2-(*o*-Chlorophenoxy)propanoic acid | H/I/T=98/2/0.1 | 1.0 | 230 | 25 | 8.433 | 8.783 | 1.81 | 1.93 | 1.06 | 1.03 | C |
| A-11 | 2-(4-Hydroxyphenoxy)propanoic acid | H/I/T=90/10/0.1 | 1.0 | 230 | 25 | 6.592 | 7.442 | 1.20 | 1.48 | 1.24 | 3.90 | A |
| A-12 | N-Cbz-Leucine | H/E/T=90/10/0.1 | 1.0 | 230 | 25 | 7.258 | - | 1.42 | - | 1.00 | - | D |
| A-13 | N-Cbz-Valine | H/I/T=80/20/0.1 | 1.0 | 230 | 25 | 6.533 | 6.708 | 1.18 | 1.24 | 1.05 | - | C |
| A-14 | N-Cbz-Methionine | H/E/T=90/10/0.1 | 1.0 | 230 | 25 | 12.917 | 22.200 | 3.31 | 6.40 | 1.94 | 3.49 | A |
| A-15 | N-Cbz-Norleuicine | H/I/T=90/10/0.1 | 1.0 | 230 | 25 | 12.258 | 14.167 | 3.09 | 3.72 | 1.21 | 2.32 | A |
| A-16 | N-Cbz-Norvaline | H/E/T=90/10/0.1 | 1.0 | 230 | 25 | 8.242 | 8.967 | 1.75 | 1.99 | 1.14 | 1.31 | C |
| B-1 | Propranolol | H/I/D=90/10/0.1 | 1.0 | 230 | 25 | 6.508 | 7.142 | 1.17 | 1.38 | 1.18 | 2.18 | A |
| B-2 | Propafenone | H/E/D=70/30/0.1 | 1.0 | 254 | 25 | 6.733 | 9.275 | 1.24 | 2.09 | 1.68 | 6.16 | A |
| B-3 | Oxprenolol | H/I/D=80/20/0.1 | 1.0 | 254 | 25 | 5.350 | 7.258 | 0.78 | 1.42 | 1.81 | 6.68 | A |
| B-4 | Alprenolol | H/E/D=95/5/0.1 | 1.0 | 230 | 25 | 5.750 | 6.375 | 0.81 | 1.03 | 1.27 | 2.47 | A |
| B-5 | Pindolol | H/E/D=90/10/0.1 | 1.0 | 230 | 25 | 12.467 | 13.117 | 3.16 | 3.37 | 1.07 | 1.05 | C |
| B-6 | Atenolol | H/E/D=70/30/0.1 | 1.0 | 254 | 25 | 8.333 | 8.683 | 1.78 | 1.89 | 1.07 | 0.81 | C |
| B-7 | Nitrendipine | H/E/D=93/7/0.1 | 1.0 | 254 | 40 | 5.792 | 7.908 | 0.93 | 1.64 | 1.76 | 3.19 | A |
| B-8 | Manidipine | H/I/D=90/10/0.1 | 1.0 | 254 | 40 | 18.858 | 29.133 | 5.29 | 8.71 | 1.65 | 1.57 | B |
| B-9 | Benidipine | H/I/D=90/10/0.1 | 1.0 | 254 | 40 | 11.567 | 20.658 | 2.86 | 5.89 | 2.06 | 1.66 | B |
| B-10 | Nilvadipine | H/I/D=90/10/0.1 | 1.0 | 254 | 40 | 8.125 | - | 1.71 | - | 1.00 | - | D |
| B-11 | Nicardipine | H/E/D=93/7/0.1 | 1.0 | 254 | 40 | 9.050 | 9.383 | 2.02 | 2.13 | 1.06 | 0.98 | C |
| B-12 | Amlodipine | H/I/MEA=80/20/0.1 | 1.0 | 254 | 40 | 8.258 | 9.450 | 1.75 | 2.15 | 1.23 | 3.22 | A |
| B-13 | Nisoldipine | H/I/D=90/10/0.1 | 1.0 | 254 | 40 | 9.025 | 10.425 | 2.01 | 2.48 | 1.23 | 1.79 | B |
| B-14 | Nimodipine | H/I/D=70/30/0.1 | 1.0 | 254 | 40 | 4.367 | 8.983 | 0.46 | 1.99 | 4.38 | 2.10 | A |
| B-15 | Cisapride | H/I/D=70/30/0.1 | 1.0 | 220 | 25 | 17.483 | 30.658 | 4.83 | 9.22 | 1.91 | 7.05 | A |
| B-16 | Sulpiride | H/E/D=70/30/0.1 | 1.0 | 254 | 25 | 20.867 | 22.758 | 5.96 | 6.59 | 1.11 | 1.71 | B |
| B-17 | Troxipide | H/I/D=50/50/0.1 | 1.0 | 230 | 25 | 8.283 | 9.983 | 1.76 | 2.33 | 1.32 | 2.99 | A |
| B-18 | Indapamide | H/E/D=70/30/0.1 | 1.0 | 254 | 25 | 9.242 | 9.950 | 2.08 | 2.32 | 1.11 | 1.30 | C |
| B-19 | Profenamine | H/E/D=100/0.1/0.1 | 1.0 | 254 | 25 | 5.683 | 6.133 | 0.89 | 1.04 | 1.17 | 1.81 | B |

**TABLE S1-D** (Continued)

| Sample No. | Sample | Eluent*^a^* | Flow rate*^b^* | Detect*^b^* | Temp.*^b^* | t1 | t2 | k1 | k2 | *α* | *Rs* | Separation*^c^* |
| --- | --- | --- | --- | --- | --- | --- | --- | --- | --- | --- | --- | --- |
| B-20 | Thioridazine | H/E/D=100/1/0.1 | 1.0 | 254 | 25 | 9.875 | 10.758 | 2.29 | 2.59 | 1.13 | 1.96 | B |
| B-21 | Dimetotiazine | H/I/D=80/20/0.1 | 1.0 | 254 | 25 | 16.133 | 18.475 | 4.38 | 5.16 | 1.18 | 3.35 | A |
| B-22 | Alimemazine | H/I/D=100/0.1/0.1 | 1.0 | 254 | 25 | 8.392 | 8.775 | 1.80 | 1.93 | 1.07 | - | C |
| N-1 | 5-Methyl-5-phenylhydantoin | H/E=70/30 | 1.0 | 220 | 25 | 6.292 | 8.767 | 1.10 | 1.92 | 1.75 | 7.58 | A |
| N-2 | Ethotoin | H/E=90/10 | 1.0 | 220 | 25 | 14.775 | 17.408 | 3.93 | 4.80 | 1.22 | 4.28 | A |
| N-3 | γ-Phenyl-γ-butyrolactone | H/I=85/15 | 1.0 | 220 | 25 | 13.801 | 16.252 | 3.60 | 4.42 | 1.23 | 4.37 | A |
| N-4 | Pantoyl lactone | H/I=90/10 | 1.0 | 220 | 25 | 12.933 | 17.000 | 3.31 | 4.67 | 1.41 | 3.26 | A |
| N-5 | 4-Benzoyloxy-2-azetidinone | H/I=90/10 | 1.0 | 220 | 25 | 28.733 | 32.367 | 8.58 | 9.79 | 1.14 | 2.97 | A |
| B-23 | Ranolazine | H/E/D=50/50/0.1 | 1.0 | 230 | 25 | 10.029 | 13.059 | 2.34 | 3.35 | 1.43 | 3.25 | A |
| Z-1 | Cetirizine | H/E/A/D=50/50/0.1/0.1 | 1.0 | 230 | 25 | 7.733 | - | 1.58 | - | 1.00 | - | D |
| B-24 | Hydroxyzine | H/I/D=80/20/0.1 | 1.0 | 230 | 25 | 7.633 | 11.358 | 1.54 | 2.79 | 1.80 | 8.24 | A |
| B-25 | Meclizine | H/I/D=95/5/0.1 | 1.0 | 254 | 25 | 3.892 | 4.025 | 0.30 | 0.34 | 1.15 | 0.77 | C |
| B-26 | Methychlothiazide | H/E/D=80/20/0.1 | 1.0 | 230 | 25 | 18.058 | 23.950 | 5.02 | 6.98 | 1.39 | 3.93 | A |
| B-27 | Cyclopenthiazide | H/E/D=80/20/0.1 | 1.0 | 230 | 25 | 10.567 | 16.608 | 2.52 | 4.54 | 1.80 | 6.75 | A |
| B-28 | Ethiazide | H/E/iPrNH2=80/20/0.1 | 1.0 | 230 | 25 | 12.942 | 21.267 | 3.31 | 6.09 | 1.84 | 4.20 | A |
| B-29 | Benzylhydrochlorothiazide | H/E/D=80/20/0.1 | 1.0 | 230 | 25 | 16.492 | 21.500 | 4.50 | 6.17 | 1.37 | 3.52 | A |
| B-30 | Mefruside | H/I/D=50/50/0.1 | 1.0 | 230 | 25 | 13.458 | 16.417 | 3.49 | 4.47 | 1.28 | 2.45 | A |
| B-31 | Polythiazide | H/E/D=80/20/0.1 | 1.0 | 230 | 25 | 10.758 | 16.417 | 2.59 | 4.47 | 1.73 | 5.81 | A |
| B-32 | Chlorprenaline | H/I/D=98/2/0.1 | 1.0 | 254 | 25 | 8.717 | 9.167 | 1.91 | 2.06 | 1.08 | 1.52 | B |
| B-33 | Tulobutenol | H/I/D=100/1/0.1 | 1.0 | 254 | 25 | 7.942 | 8.592 | 1.65 | 1.86 | 1.13 | 1.54 | B |
| B-34 | Labetalol | H/E/D=90/10/0.1 | 1.0 | 230 | 25 | 11.758 | 13.000 | 2.92 | 3.33 | 1.14 | - | C |
| B-35 | Ritodrine | H/E/D=90/10/0.1 | 1.0 | 275 | 25 | 11.612 | 11.979 | 2.47 | 2.58 | 1.04 | - | C |
| N-12 | *trans*-Stilbene oxide | H/I=90/10 | 1.0 | 254 | 25 | 4.751 | 6.537 | 0.55 | 1.14 | 2.05 | 11.14 | A |
| N-13 | 1-(9-Anthryl)-2,2,2-trifluoroethanol | H/I=90/10 | 1.0 | 254 | 25 | 4.177 | 4.313 | 0.37 | 0.41 | 1.12 | 0.82 | C |
| N-14 | Tröger’s base | H/I=90/10 | 1.0 | 254 | 25 | 5.994 | 7.203 | 0.96 | 1.36 | 1.41 | 5.66 | A |
| N-15 | Benzoin | H/I=90/10 | 1.0 | 254 | 25 | 19.203 | 21.114 | 5.28 | 5.90 | 1.12 | 2.92 | A |
| N-16 | Flavanone | H/I=90/10 | 1.0 | 254 | 25 | 8.960 | 10.743 | 1.93 | 2.51 | 1.30 | 5.34 | A |
| N-17 | 1-Naphthylethanol | H/I=90/10 | 1.0 | 254 | 25 | 6.151 | 6.432 | 1.00 | 1.10 | 1.09 | 1.52 | B |
| N-18 | Cobalt tris(acetylacetonate) | H/I=90/10 | 1.0 | 254 | 25 | 5.869 | 6.519 | 0.92 | 1.13 | 1.23 | 2.42 | A |
| N-19 | Phenyl vinyl sulfoxide | H/I=90/10 | 1.0 | 254 | 25 | 22.746 | 26.029 | 6.44 | 7.51 | 1.17 | 4.53 | A |
| N-20 | 1,1’-Bi-2-naphthol | H/I=90/10 | 1.0 | 254 | 25 | 9.201 | 9.525 | 2.01 | 2.12 | 1.05 | 0.91 | C |
| N-21 | 2-Phenylcyclohexanone | H/I=90/10 | 1.0 | 254 | 25 | 7.359 | 7.866 | 1.41 | 1.57 | 1.12 | 2.41 | A |

*^a^* “H” means *n*-hexane, “E” means ethanol, “I” means 2-propanol, “T” means trifluoroacetic acid, “D” means diethylamine and “MEA” means 2-aminoethanol.

*^b^* Flow rate’s unit is “mL/min”, Detect’s unit is “nm UV” and Temp.’s unit is “℃”.

*^c^* “A” means baseline separation, “B” means nearly baseline separation, “C” means partial separation and “D” means no separation.

**TABLE S1-E** The details of analytical conditions and chromatographic results of 67 compounds analysis with CHIRALPAK IE

| Sample No. | Sample | Eluent*^a^* | Flow rate*^b^* | Detect*^b^* | Temp.*^b^* | t1 | t2 | k1 | k2 | *α* | *Rs* | Separation*^c^* |
| --- | --- | --- | --- | --- | --- | --- | --- | --- | --- | --- | --- | --- |
| A-1 | Ketoprofen | H/E/T=95/5/0.1 | 1.0 | 230 | 25 | 22.817 | 24.542 | 6.61 | 7.18 | 1.09 | 2.14 | A |
| A-2 | Naproxen | H/E/T=95/5/0.1 | 1.0 | 230 | 25 | 9.917 | 10.867 | 2.31 | 2.62 | 1.14 | 2.17 | A |
| A-3 | Tiaprofenic acid | H/I/T=90/10/0.1 | 1.0 | 230 | 25 | 23.908 | 29.450 | 6.97 | 8.82 | 1.27 | 4.59 | A |
| A-4 | Fenoprofen | H/I/T=95/5/0.1 | 1.0 | 230 | 25 | 7.500 | 7.683 | 1.50 | 1.56 | 1.04 | - | C |
| A-5 | Flurbiprofen | H/E/T=98/2/0.1 | 1.0 | 254 | 25 | 11.600 | 15.733 | 2.87 | 4.24 | 1.48 | 8.78 | A |
| A-6 | 2-Phenylpropanoic acid | H/E/T=98/2/0.1 | 1.0 | 230 | 25 | 8.475 | 8.825 | 1.83 | 1.94 | 1.06 | 1.36 | C |
| A-7 | Ibuprofen | H/E/T=98/2/0.1 | 1.0 | 230 | 25 | 6.833 | 7.008 | 1.28 | - | 1.00 | - | C |
| A-8 | Pranoprofen | H/E/T=80/20/0.1 | 1.0 | 230 | 25 | 15.675 | 18.708 | 4.23 | 5.24 | 1.24 | 4.76 | A |
| A-9 | 2-Phenoxypropanoic acid | H/I/T=98/2/0.1 | 1.0 | 230 | 25 | 10.908 | 13.600 | 2.64 | 3.53 | 1.34 | 6.41 | A |
| A-10 | 2-(*o*-Chlorophenoxy)propanoic acid | H/I/T=95/5/0.1 | 1.0 | 230 | 25 | 5.867 | - | 0.96 | - | 1.00 | - | D |
| A-11 | 2-(4-Hydroxyphenoxy)propanoic acid | H/I/T=95/5/0.1 | 1.0 | 230 | 25 | 31.825 | 34.867 | 9.61 | 10.62 | 1.11 | 2.06 | A |
| A-12 | N-Cbz-Leucine | H/I/T=90/10/0.1 | 1.0 | 230 | 25 | 11.625 | 12.042 | 2.88 | 3.01 | 1.05 | 0.78 | C |
| A-13 | N-Cbz-Valine | H/E/T=90/10/0.1 | 1.0 | 230 | 25 | 11.942 | 12.958 | 2.98 | 3.32 | 1.11 | 1.94 | B |
| A-14 | N-Cbz-Methionine | H/E/T=80/20/0.1 | 1.0 | 230 | 25 | 8.317 | 8.917 | 1.77 | 1.97 | 1.11 | 1.90 | B |
| A-15 | N-Cbz-Norleuicine | H/I/T=90/10/0.1 | 1.0 | 230 | 25 | 11.825 | 12.117 | 2.94 | 3.04 | 1.03 | - | C |
| A-16 | N-Cbz-Norvaline | H/I/T=90/10/0.1 | 1.0 | 230 | 25 | 12.633 | 12.958 | 3.21 | 3.32 | 1.03 | - | C |
| B-1 | Propranolol | H/E/D=93/7/0.1 | 1.0 | 230 | 25 | 8.133 | 8.258 | 1.71 | 1.75 | 1.02 | - | C |
| B-2 | Propafenone | H/I/D=80/20/0.1 | 1.0 | 230 | 25 | 12.042 | 13.767 | 3.01 | 3.59 | 1.19 | 3.19 | A |
| B-3 | Oxprenolol | H/E/D=93/7/0.1 | 1.0 | 230 | 25 | 11.142 | 11.992 | 2.42 | 2.68 | 1.11 | 2.00 | A |
| B-4 | Alprenolol | H/E/D=95/5/0.1 | 1.0 | 230 | 25 | 7.033 | 7.492 | 1.34 | 1.50 | 1.11 | 1.71 | B |
| B-5 | Pindolol | H/E/D=90/10/0.1 | 1.0 | 230 | 25 | 17.400 | 18.225 | 4.80 | 5.08 | 1.06 | 1.14 | C |
| B-6 | Atenolol | H/E/D=80/20/0.1 | 1.0 | 230 | 25 | 25.742 | - | 7.58 | - | 1.00 | - | D |
| B-7 | Nitrendipine | H/I/D=80/20/0.1 | 1.0 | 254 | 40 | 5.882 | 5.999 | 0.91 | 0.95 | 1.04 | - | C |
| B-8 | Manidipine | H/I/D=80/20/0.1 | 1.0 | - | 40 | 7.776 | 7.975 | 1.16 | 1.60 | 1.04 |  | C |
| B-9 | Benidipine | H/I/D=95/5/0.1 | 1.0 | 254 | 40 | 32.419 | 32.419 | 8.89 | 9.15 | 1.03 | - | C |
| B-10 | Nilvadipine | H/I/D=95/5/0.1 | 1.0 | 254 | 40 | 19.552 | 20.586 | 5.29 | 5.62 | 1.06 | 1.210 | C |
| B-11 | Nicardipine | H/I/D=90/10/0.1 | 1.0 | 254 | 40 | 14.418 | - | 3.58 | - | 1.00 | - | D |
| B-12 | Amlodipine | H/I/MEA=90/10/0.1 | 1.0 | 254 | 40 | 10.475 | 11.247 | 2.36 | 2.61 | 1.11 | 2.071 | A |
| B-13 | Nisoldipine | H/E/D=95/5/0.1 | 1.0 | 254 | 40 | 9.38 | 9.786 | 2.15 | 2.29 | 1.06 | 1.259 | C |
| B-14 | Nimodipine | H/I/D=80/20/0.1 | 1.0 | 254 | 40 | 6.08 | - | 0.98 | - | 1.00 | - | D |
| B-15 | Cisapride | H/E/D=80/20/0.1 | 1.0 | 254 | 25 | 26.050 | 28.117 | 7.00 | 7.63 | 1.09 | 1.67 | B |
| B-16 | Sulpiride | H/E/D=50/50/0.1 | 1.0 | 254 | 25 | 26.575 | 28.308 | 7.86 | 8.44 | 1.07 | 1.28 | C |
| B-17 | Troxipide | H/I/D=50/50/0.1 | 1.0 | 230 | 25 | 13.542 | 15.483 | 3.51 | 4.16 | 1.18 | 2.22 | A |
| B-18 | Indapamide | H/E/D=80/20/0.1 | 1.0 | 230 | 25 | 29.375 | 31.617 | 8.79 | 9.54 | 1.09 | 1.57 | B |
| B-19 | Profenamine | H/E/D=100/0.1/0.1 | 1.0 | 254 | 25 | 6.892 | 7.708 | 1.30 | 1.57 | 1.21 | 2.01 | A |

**TABLE S1-E** (Continued)

| Sample No. | Sample | Eluent*^a^* | Flow rate*^b^* | Detect*^b^* | Temp.*^b^* | t1 | t2 | k1 | k2 | *α* | *Rs* | Separation*^c^* |
| --- | --- | --- | --- | --- | --- | --- | --- | --- | --- | --- | --- | --- |
| B-20 | Thioridazine | H/E/D=100/1/0.1 | 1.0 | 254 | 25 | 23.417 | 24.900 | 6.81 | 7.30 | 1.07 | 0.87 | C |
| B-21 | Dimetotiazine | H/I/D=70/30/0.1 | 1.0 | 254 | 25 | 15.250 | 17.067 | 4.08 | 4.69 | 1.15 | 2.75 | A |
| B-22 | Alimemazine | H/I/D=100/0.1/0.1 | 1.0 | 254 | 25 | 13.733 | - | 3.58 | - | 1.00 | - | D |
| N-1 | 5-Methyl-5-phenylhydantoin | H/E=70/30 | 1.0 | 220 | 25 | 6.608 | 10.400 | 1.20 | 2.47 | 2.05 | 10.63 | A |
| N-2 | Ethotoin | H/E=90/10 | 1.0 | 220 | 25 | 15.517 | 15.917 | 4.17 | 4.31 | 1.03 | - | C |
| N-3 | γ-Phenyl-γ-butyrolactone | H/I=85/15 | 1.0 | 220 | 25 | 20.013 | 20.474 | 5.67 | 5.82 | 1.03 | - | C |
| N-4 | Pantoyl lactone | H/I=90/10 | 1.0 | 220 | 25 | 16.942 | 18.183 | 4.65 | 5.06 | 1.09 | 1.41 | C |
| N-5 | 4-Benzoyloxy-2-azetidinone | H/E=80/20 | 1.0 | 220 | 25 | 13.808 | 14.592 | 3.60 | 3.86 | 1.07 | 1.30 | C |
| B-23 | Ranolazine | H/I/D=50/50/0.1 | 1.0 | 230 | 25 | 22.858 | 37.390 | 6.62 | 11.46 | 1.73 | 7.58 | A |
| Z-1 | Cetirizine | H/E/A/D=60/40/0.1/0.1 | 1.0 | 230 | 25 | 30.950 | 38.742 | 8.50 | 10.89 | 1.28 | 4.00 | A |
| B-24 | Hydroxyzine | H/I/D=80/20/0.1 | 1.0 | 230 | 25 | 7.755 | 8.349 | 1.59 | 1.78 | 1.12 | 1.96 | B |
| B-25 | Meclizine | H/I/D=95/5/0.1 | 1.0 | 254 | 25 | 3.842 | 3.933 | 0.28 | 0.31 | 1.11 | - | C |
| B-26 | Methychlothiazide | H/I/D=70/30/0.1 | 1.0 | 230 | 25 | 23.000 | 24.083 | 6.67 | 7.03 | 1.05 | - | C |
| B-27 | Cyclopenthiazide | H/E/D=60/40/0.1 | 1.0 | 230 | 25 | 8.867 | 13.058 | 1.96 | 3.35 | 1.71 | 7.06 | A |
| B-28 | Ethiazide | H/I/D=80/20/0.1 | 1.0 | 230 | 25 | 11.950 | 28.642 | 2.98 | 8.55 | 2.87 | 3.41 | A |
| B-29 | Benzylhydrochlorothiazide | H/E/D=60/40/0.1 | 1.0 | 230 | 25 | 12.008 | 15.583 | 3.00 | 4.19 | 1.40 | 4.60 | A |
| B-30 | Mefruside | H/I/D=50/50/0.1 | 1.0 | 230 | 25 | 49.017 | 86.208 | 15.34 | 27.74 | 1.81 | 8.05 | A |
| B-31 | Polythiazide | H/E/D=60/40/0.1 | 1.0 | 230 | 25 | 9.950 | 11.292 | 2.32 | 2.76 | 1.19 | 2.27 | B |
| B-32 | Chlorprenaline | H/E/D=98/2/0.1 | 1.0 | 254 | 25 | 9.483 | 9.783 | 2.16 | 2.26 | 1.05 | 0.80 | C |
| B-33 | Tulobutenol | H/E/D=400/1/0.4 | 1.0 | 254 | 25 | 25.817 | 27.600 | 7.61 | 8.20 | 1.08 | 1.41 | C |
| B-34 | Labetalol | H/I/D=80/20/0.1 | 1.0 | 230 | 25 | 10.150 | 10.608 | 2.38 | 2.54 | 1.06 | - | C |
| B-35 | Ritodrine | H/E/D=90/10/0.1 | 1.0 | 275 | 25 | 19.127 | - | 4.40 | - | 1.00 | - | D |
| N-12 | *trans*-Stilbene oxide | H/I=90/10 | 1.0 | 254 | 25 | 4.554 | 4.751 | 0.49 | 0.55 | 1.13 | 1.59 | B |
| N-13 | 1-(9-Anthryl)-2,2,2-trifluoroethanol | H/I=90/10 | 1.0 | 254 | 25 | 4.143 | - | 0.35 | - | 1.00 | - | D |
| N-14 | Tröger’s base | H/I=90/10 | 1.0 | 254 | 25 | 6.622 | 7.100 | 1.17 | 1.32 | 1.13 | 2.03 | A |
| N-15 | Benzoin | H/I=90/10 | 1.0 | 254 | 25 | 17.237 | - | 4.64 | - | 1.00 | - | D |
| N-16 | Flavanone | H/I=90/10 | 1.0 | 254 | 25 | 8.660 | 10.421 | 1.84 | 2.41 | 1.31 | 5.74 | A |
| N-17 | 1-Naphthylethanol | H/I=90/10 | 1.0 | 254 | 25 | 5.877 | - | 0.92 | - | 1.00 | - | D |
| N-18 | Cobalt tris(acetylacetonate) | H/I=90/10 | 1.0 | 254 | 25 | 7.801 | 8.261 | 1.55 | 1.70 | 1.10 | 1.48 | C |
| N-19 | Phenyl vinyl sulfoxide | H/I=90/10 | 1.0 | 254 | 25 | 23.100 | - | 6.56 | - | 1.00 | - | D |
| N-20 | 1,1’-Bi-2-naphthol | H/I=90/10 | 1.0 | 254 | 25 | 7.020 | 8.120 | 1.30 | 1.66 | 1.28 | 4.09 | A |
| N-21 | 2-Phenylcyclohexanone | H/I=90/10 | 1.0 | 254 | 25 | 7.923 | 8.087 | 1.59 | 1.65 | 1.03 | - | C |

*^a^* “H” means *n*-hexane, “E” means ethanol, “I” means 2-propanol, “T” means trifluoroacetic acid, “D” means diethylamine and “MEA” means 2-aminoethanol.

*^b^* Flow rate’s unit is “mL/min”, Detect’s unit is “nm UV” and Temp.’s unit is “℃”.

*^c^* “A” means baseline separation, “B” means nearly baseline separation, “C” means partial separation and “D” means no separation.

**TABLE S1-F** The details of analytical conditions and chromatographic results of 67 compounds analysis with CHIRALPAK IF

| Sample No. | Sample | Eluent*^a^* | Flow rate*^b^* | Detect*^b^* | Temp.*^b^* | t1 | t2 | k1 | k2 | *α* | *Rs* | Separation*^c^* |
| --- | --- | --- | --- | --- | --- | --- | --- | --- | --- | --- | --- | --- |
| A-1 | Ketoprofen | H/E/T=93/7/0.1 | 1.0 | 254 | 25 | 14.075 | 16.992 | 3.69 | 4.66 | 1.26 | 7.86 | A |
| A-2 | Naproxen | H/E/T=93/7/0.1 | 1.0 | 254 | 25 | 8.658 | 9.317 | 1.89 | 2.11 | 1.12 | 1.92 | B |
| A-3 | Tiaprofenic acid | H/E/T=93/7/0.1 | 1.0 | 254 | 25 | 29.608 | 33.658 | 8.87 | 10.22 | 1.15 | 3.52 | A |
| A-4 | Fenoprofen | H/E/T=98/2/0.1 | 1.0 | 254 | 25 | 10.588 | 11.291 | 2.53 | 2.76 | 1.09 | 1.92 | B |
| A-5 | Flurbiprofen | H/E/T=98/2/0.1 | 1.0 | 254 | 25 | 14.458 | 19.917 | 3.82 | 5.64 | 1.48 | 9.11 | A |
| A-6 | 2-Phenylpropanoic acid | H/E/T=98/2/0.1 | 1.0 | 254 | 25 | 9.908 | 10.325 | 2.30 | 2.44 | 1.06 | 1.26 | C |
| A-7 | Ibuprofen | H/I/T=98/2/0.1 | 1.0 | 230 | 25 | 8.467 | - | 1.82 | - | 1.00 | - | D |
| A-8 | Pranoprofen | H/E/T=80/20/0.1 | 1.0 | 230 | 25 | 16.075 | 18.917 | 4.36 | 5.31 | 1.22 | 3.73 | A |
| A-9 | 2-Phenoxypropanoic acid | H/I/T=98/2/0.1 | 1.0 | 230 | 25 | 10.800 | 13.150 | 2.60 | 3.38 | 1.30 | 5.11 | A |
| A-10 | 2-(*o*-Chlorophenoxy)propanoic acid | H/E/T=98/2/0.1 | 1.0 | 254 | 25 | 9.800 | 10.225 | 2.27 | 2.41 | 1.06 | 1.28 | C |
| A-11 | 2-(4-Hydroxyphenoxy)propanoic acid | H/E/T=93/7/0.1 | 1.0 | 230 | 25 | 11.367 | 12.675 | 2.79 | 3.23 | 1.16 | 2.34 | B |
| A-12 | N-Cbz-Leucine | H/E/T=90/10/0.1 | 1.0 | 230 | 25 | 8.983 | 9.617 | 1.99 | 2.21 | 1.11 | 1.44 | C |
| A-13 | N-Cbz-Valine | H/E/T=90/10/0.1 | 1.0 | 230 | 25 | 11.542 | 12.692 | 2.85 | 3.23 | 1.13 | 1.97 | A |
| A-14 | N-Cbz-Methionine | H/E/T=90/10/0.1 | 1.0 | 230 | 25 | 15.900 | 18.642 | 4.30 | 5.21 | 1.21 | 3.65 | A |
| A-15 | N-Cbz-Norleuicine | H/I/T=90/10/0.1 | 1.0 | 230 | 25 | 10.308 | 11.258 | 2.44 | 2.75 | 1.13 | 1.78 | B |
| A-16 | N-Cbz-Norvaline | H/I/T=90/10/0.1 | 1.0 | 230 | 25 | 10.633 | 12.258 | 2.54 | 3.09 | 1.21 | 2.98 | A |
| B-1 | Propranolol | H/E/D=93/7/0.1 | 1.0 | 230 | 25 | 8.175 | 8.600 | 1.73 | 1.87 | 1.08 | 1.21 | C |
| B-2 | Propafenone | H/I/D=80/20/0.1 | 1.0 | 230 | 25 | 11.183 | 20.000 | 2.73 | 5.67 | 2.08 | 6.88 | A |
| B-3 | Oxprenolol | H/I/D=90/10/0.1 | 1.0 | 230 | 25 | 7.625 | 12.000 | 1.54 | 3.00 | 1.95 | 6.20 | A |
| B-4 | Alprenolol | H/I/D=98/2/0.1 | 1.0 | 230 | 25 | 13.642 | 16.158 | 3.55 | 4.39 | 1.24 | 2.89 | A |
| B-5 | Pindolol | H/E/D=90/10/0.1 | 1.0 | 230 | 25 | 19.458 | 21.742 | 5.49 | 6.25 | 1.14 | 2.49 | A |
| B-6 | Atenolol | H/E/D=80/20/0.1 | 1.0 | 230 | 25 | 17.792 | - | 4.93 | - | 1.00 | - | D |
| B-7 | Nitrendipine | H/E/D=93/7/0.1 | 1.0 | 254 | 40 | 8.117 | 8.221 | 1.64 | 1.68 | 1.02 | - | C |
| B-8 | Manidipine | H/I/D=80/20/0.1 | 1.0 | - | 40 | 5.651 | - | 0.91 | - | 1.00 | - | D |
| B-9 | Benidipine | H/I/D=90/10/0.1 | 1.0 | 254 | 40 | 9.168 | - | 17.11 | - | 1.00 | - | D |
| B-10 | Nilvadipine | H/I/D=90/10/0.1 | 1.0 | 254 | 40 | 7.589 | 7.739 | 1.56 | 1.61 | 1.03 | - | C |
| B-11 | Nicardipine | H/E/D=93/7/0.1 | 1.0 | 254 | 40 | 9.589 | - | 2.24 | - | 1.00 | - | D |
| B-12 | Amlodipine | H/I/MEA=80/20/0.1 | 1.0 | 254 | 40 | 8.03 | 8.301 | 1.69 | 1.78 | 1.05 | - | C |
| B-13 | Nisoldipine | H/I/D=90/10/0.1 | 1.0 | 254 | 40 | 8.084 | - | 1.73 | - | 1.00 | - | D |
| B-14 | Nimodipine | H/I/D=90/10/0.1 | 1.0 | 254 | 40 | 8.558 | 8.846 | 1.89 | 1.98 | 1.05 | - | C |
| B-15 | Cisapride | H/E/D=80/20/0.1 | 1.0 | 230 | 25 | 13.750 | 15.842 | 3.58 | 4.28 | 1.19 | 2.90 | A |
| B-16 | Sulpiride | H/E/D=60/40/0.1 | 1.0 | 230 | 25 | 14.508 | 16.242 | 3.84 | 4.41 | 1.15 | 1.83 | B |
| B-17 | Troxipide | H/E/D=60/40/0.1 | 1.0 | 230 | 25 | 8.325 | 10.000 | 1.78 | 2.33 | 1.31 | 2.55 | A |
| B-18 | Indapamide | H/E/D=60/40/0.1 | 1.0 | 230 | 25 | 7.467 | 9.342 | 1.49 | 2.11 | 1.42 | 1.93 | B |
| B-19 | Profenamine | H/E/D=100/0.1/0.1 | 1.0 | 254 | 25 | 6.433 | 6.858 | 1.14 | 1.29 | 1.12 | 1.54 | B |

**TABLE S1-F** (Continued)

| Sample No. | Sample | Eluent*^a^* | Flow rate*^b^* | Detect*^b^* | Temp.*^b^* | t1 | t2 | k1 | k2 | *α* | *Rs* | Separation*^c^* |
| --- | --- | --- | --- | --- | --- | --- | --- | --- | --- | --- | --- | --- |
| B-20 | Thioridazine | H/E/D=100/1/0.1 | 1.0 | 254 | 25 | 15.550 | 16.433 | 4.18 | 4.48 | 1.07 | 1.06 | C |
| B-21 | Dimetotiazine | H/E/D=80/20/0.1 | 1.0 | 254 | 25 | 12.525 | 13.367 | 3.18 | 3.46 | 1.09 | 1.60 | B |
| B-22 | Alimemazine | H/I/D=100/0.1/0.1 | 1.0 | 254 | 25 | 10.883 | - | 2.63 | - | 1.00 | - | D |
| N-1 | 5-Methyl-5-phenylhydantoin | H/E=70/30 | 1.0 | 220 | 25 | 7.850 | 18.125 | 1.62 | 5.04 | 3.12 | 18.46 | A |
| N-2 | Ethotoin | H/I=80/20 | 1.0 | 220 | 25 | 8.217 | 8.975 | 1.74 | 1.99 | 1.15 | 2.16 | A |
| N-3 | γ-Phenyl-γ-butyrolactone | H/I=85/15 | 1.0 | 220 | 25 | 18.181 | 18.939 | 5.06 | 5.31 | 1.05 | 1.27 | C |
| N-4 | Pantoyl lactone | H/I=90/10 | 1.0 | 220 | 25 | 13.817 | 17.058 | 3.61 | 4.69 | 1.30 | 4.61 | A |
| N-5 | 4-Benzoyloxy-2-azetidinone | H/E=80/20 | 1.0 | 220 | 25 | 22.958 | 34.100 | 6.65 | 10.37 | 1.56 | 7.88 | A |
| B-23 | Ranolazine | H/I/D=50/50/0.1 | 1.0 | 230 | 25 | 14.323 | 23.646 | 3.77 | 6.88 | 1.82 | 7.58 | A |
| Z-1 | Cetirizine | H/E/A/D=50/50/0.1/0.1 | 1.0 | 230 | 25 | 16.458 | - | 4.49 | - | 1.00 | - | D |
| B-24 | Hydroxyzine | H/I/D=80/20/0.1 | 1.0 | 230 | 25 | 7.062 | 8.466 | 1.35 | 1.82 | 1.35 | 3.46 | A |
| B-25 | Meclizine | H/I/D=95/5/0.1 | 1.0 | 254 | 25 | 4.083 | - | 0.36 | - | 1.00 | - | D |
| B-26 | Methychlothiazide | H/E/D=60/40/0.1 | 1.0 | 230 | 25 | 11.450 | 22.492 | 2.82 | 6.50 | 2.31 | 6.76 | A |
| B-27 | Cyclopenthiazide | H/E/D=60/40/0.1 | 1.0 | 230 | 25 | 6.025 | 9.942 | 1.01 | 2.31 | 2.29 | 4.86 | A |
| B-28 | Ethiazide | H/E/D=60/40/0.1 | 1.0 | 230 | 25 | 6.133 | 15.983 | 1.04 | 4.33 | 4.14 | 3.35 | A |
| B-29 | Benzylhydrochlorothiazide | H/E/D=60/40/0.1 | 1.0 | 230 | 25 | 7.858 | 14.908 | 1.62 | 3.97 | 2.45 | 4.57 | A |
| B-30 | Mefruside | H/I/D=50/50/0.1 | 1.0 | 230 | 25 | 30.867 | 49.342 | 9.29 | 15.45 | 1.66 | 5.45 | A |
| B-31 | Polythiazide | H/E/D=60/40/0.1 | 1.0 | 230 | 25 | 6.708 | 11.658 | 1.24 | 2.89 | 2.33 | 5.12 | A |
| B-32 | Chlorprenaline | H/E/D=100/1/0.1 | 1.0 | 254 | 25 | 14.847 | 15.143 | 3.95 | 4.05 | 1.02 | 0.29 | C |
| B-33 | Tulobutenol | H/E/D=100/1/0.1 | 1.0 | 254 | 25 | 9.583 | 11.242 | 2.19 | 2.75 | 1.25 | 3.11 | A |
| B-34 | Labetalol | H/E/D=90/10/0.1 | 1.0 | 230 | 25 | 14.808 | 18.308 | 3.94 | 5.10 | 1.30 | - | C |
| B-35 | Ritodrine | H/E/D=90/10/0.1 | 1.0 | 275 | 25 | 15.959 | - | 3.91 | - | 1.00 | - | D |
| N-12 | *trans*-Stilbene oxide | H/I=90/10 | 1.0 | 254 | 25 | 4.458 | 5.140 | 0.53 | 0.76 | 1.44 | 5.28 | A |
| N-13 | 1-(9-Anthryl)-2,2,2-trifluoroethanol | H/I=90/10 | 1.0 | 254 | 25 | 4.679 | 4.880 | 0.60 | 0.67 | 1.11 | 0.93 | C |
| N-14 | Tröger’s base | H/I=90/10 | 1.0 | 254 | 25 | 5.932 | 7.002 | 1.03 | 1.40 | 1.35 | 4.90 | A |
| N-15 | Benzoin | H/I=90/10 | 1.0 | 254 | 25 | 16.593 | 17.019 | 4.69 | 4.83 | 1.03 | - | C |
| N-16 | Flavanone | H/I=90/10 | 1.0 | 254 | 25 | 8.079 | 10.862 | 1.77 | 2.72 | 1.54 | 7.75 | A |
| N-17 | 1-Naphthylethanol | H/I=90/10 | 1.0 | 254 | 25 | 6.313 | - | 1.16 | - | 1.00 | - | D |
| N-18 | Cobalt tris(acetylacetonate) | H/I=90/10 | 1.0 | 254 | 25 | 12.061 | - | 3.13 | - | 1.00 | - | D |
| N-19 | Phenyl vinyl sulfoxide | H/I=90/10 | 1.0 | 254 | 25 | 16.389 | - | 4.62 | - | 1.00 | - | D |
| N-20 | 1,1’-Bi-2-naphthol | H/I=90/10 | 1.0 | 254 | 25 | 10.343 | 10.945 | 2.54 | 2.75 | 1.08 | 0.77 | C |
| N-21 | 2-Phenylcyclohexanone | H/I=90/10 | 1.0 | 254 | 25 | 6.746 | 6.870 | 1.31 | 1.36 | 1.03 | - | C |

*^a^* “H” means *n*-hexane, “E” means ethanol, “I” means 2-propanol, “T” means trifluoroacetic acid, “D” means diethylamine and “MEA” means 2-aminoethanol.

*^b^* Flow rate’s unit is “mL/min”, Detect’s unit is “nm UV” and Temp.’s unit is “℃”.

*^c^* “A” means baseline separation, “B” means nearly baseline separation, “C” means partial separation and “D” means no separation.

**TABLE S1-G** The details of analytical conditions and chromatographic results of 67 compounds analysis with CHIRALPAK IG

| Sample No. | Sample | Eluent*^a^* | Flow rate*^b^* | Detect*^b^* | Temp.*^b^* | t1 | t2 | k1 | k2 | *α* | *Rs* | Separation*^c^* |
| --- | --- | --- | --- | --- | --- | --- | --- | --- | --- | --- | --- | --- |
| A-1 | Ketoprofen | H/E/T=93/7/0.1 | 1.0 | 254 | 25 | 22.588 | 24.127 | 6.53 | 7.04 | 1.08 | 1.65 | B |
| A-2 | Naproxen | H/E/T=93/7/0.1 | 1.0 | 254 | 25 | 13.675 | 15.041 | 3.56 | 4.01 | 1.13 | 2.77 | A |
| A-3 | Tiaprofenic acid | H/I/T=80/20/0.1 | 1.0 | 254 | 25 | 12.020 | 12.999 | 3.01 | 3.33 | 1.11 | 1.88 | B |
| A-4 | Fenoprofen | H/I/T=90/10/0.1 | 1.0 | 254 | 25 | 6.628 | 7.488 | 1.21 | 1.50 | 1.24 | 3.17 | A |
| A-5 | Flurbiprofen | H/E/T=93/7/0.1 | 1.0 | 254 | 25 | 7.881 | 12.240 | 1.63 | 3.08 | 1.89 | 12.20 | A |
| A-6 | 2-Phenylpropanoic acid | H/E/T=98/2/0.1 | 1.0 | 230 | 25 | 12.666 | 13.646 | 3.22 | 3.55 | 1.10 | 2.20 | A |
| A-7 | Ibuprofen | H/I/T=98/2/0.1 | 1.0 | 230 | 25 | 12.604 | 12.914 | 3.20 | 3.30 | 1.03 | 0.44 | C |
| A-8 | Pranoprofen | H/E/T=80/20/0.1 | 1.0 | 230 | 25 | 20.733 | 28.974 | 5.91 | 8.66 | 1.46 | 8.16 | A |
| A-9 | 2-Phenoxypropanoic acid | H/I/T=90/10/0.1 | 1.0 | 230 | 25 | 5.079 | 7.052 | 0.69 | 1.35 | 1.95 | 8.33 | A |
| A-10 | 2-(*o*-Chlorophenoxy)propanoic acid | H/E/T=93/7/0.1 | 1.0 | 230 | 25 | 5.749 | 6.139 | 0.92 | 1.05 | 1.14 | 1.87 | B |
| A-11 | 2-(4-Hydroxyphenoxy)propanoic acid | H/I/T=90/10/0.1 | 1.0 | 230 | 25 | 10.503 | 12.912 | 2.50 | 3.30 | 1.32 | 4.82 | A |
| A-12 | N-Cbz-Leucine | H/I/T=90/10/0.1 | 1.0 | 200 | 25 | 13.246 | 14.936 | 3.42 | 3.98 | 1.16 | 2.20 | A |
| A-13 | N-Cbz-Valine | H/I/T=80/20/0.1 | 1.0 | 230 | 25 | 9.532 | 12.836 | 2.18 | 3.28 | 1.51 | 5.36 | A |
| A-14 | N-Cbz-Methionine | H/E/T=90/10/0.1 | 1.0 | 230 | 25 | 26.536 | 32.052 | 7.85 | 9.68 | 1.23 | 4.65 | A |
| A-15 | N-Cbz-Norleuicine | H/I/T=90/10/0.1 | 1.0 | 230 | 25 | 16.453 | 17.605 | 4.48 | 4.87 | 1.09 | 1.34 | C |
| A-16 | N-Cbz-Norvaline | H/E/T=90/10/0.1 | 1.0 | 230 | 25 | 15.403 | 16.114 | 4.13 | 4.37 | 1.06 | 1.14 | C |
| B-1 | Propranolol | H/E/D=93/7/0.1 | 1.0 | 230 | 25 | 9.609 | 11.335 | 2.20 | 2.78 | 1.26 | 4.34 | A |
| B-2 | Propafenone | H/E/D=80/20/0.1 | 1.0 | 230 | 25 | 12.779 | 21.447 | 3.26 | 6.15 | 1.89 | 11.91 | A |
| B-3 | Oxprenolol | H/E/D=93/7/0.1 | 1.0 | 230 | 25 | 10.741 | 16.451 | 2.58 | 4.48 | 1.74 | 11.67 | A |
| B-4 | Alprenolol | H/I/D=95/5/0.1 | 1.0 | 230 | 25 | 7.349 | 10.028 | 1.45 | 2.34 | 1.62 | 8.05 | A |
| B-5 | Pindolol | H/E/D=80/20/0.1 | 1.0 | 230 | 25 | 8.434 | 9.458 | 1.81 | 2.15 | 1.19 | 2.59 | A |
| B-6 | Atenolol | H/E/D=70/30/0.1 | 1.0 | 230 | 25 | 11.165 | 11.309 | 2.72 | 2.77 | 1.02 | 0.02 | C |
| B-7 | Nitrendipine | H/E/D=96/4/0.1 | 1.0 | 254 | 40 | 20.872 | 22.545 | 6.08 | 6.65 | 1.09 | 2.083 | A |
| B-8 | Manidipine | H/E/D=93/7/0.1 | 1.0 | 254 | 40 | 20.173 | 22.052 | 5.91 | 6.55 | 1.11 | 1.927 | B |
| B-9 | Benidipine | H/I/D=90/10/0.1 | 1.0 | 254 | 40 | 11.163 | 12.868 | 2.86 | 3.45 | 1.21 | 3.02 | A |
| B-10 | Nilvadipine | H/I/D=90/10/0.1 | 1.0 | 254 | 40 | 9.058 | 9.896 | 2.13 | 2.42 | 1.14 | 2.09 | A |
| B-11 | Nicardipine | H/I/D=90/10/0.1 | 1.0 | 254 | 40 | 14.519 | 14.851 | 4.02 | 4.14 | 1.03 | - | C |
| B-12 | Amlodipine | H/E/MEA=80/20/0.1 | 1.0 | 254 | 40 | 8.927 | 10.325 | 2.04 | 2.52 | 1.23 | 3.44 | A |
| B-13 | Nisoldipine | H/I/D=90/10/0.1 | 1.0 | 254 | 40 | 10.729 | - | 2.712 | - | 1.00 | - | D |
| B-14 | Nimodipine | H/I/D=90/10/0.1 | 1.0 | 254 | 40 | 20.872 | 22.545 | 6.08 | 6.65 | 1.09 | 2.083 | D |
| B-15 | Cisapride | H/E/D=70/30/0.1 | 1.0 | 254 | 25 | 12.612 | 18.181 | 3.20 | 5.06 | 1.58 | 4.98 | A |
| B-16 | Sulpiride | H/E/D=60/40/0.1 | 1.0 | 230 | 25 | 18.861 | 21.015 | 5.29 | 6.01 | 1.14 | 2.18 | A |
| B-17 | Troxipide | H/I/D=50/50/0.1 | 1.0 | 230 | 25 | 7.009 | 8.074 | 1.34 | 1.69 | 1.27 | 2.20 | A |
| B-18 | Indapamide | H/E/D=70/30/0.1 | 1.0 | 230 | 25 | 11.283 | 12.647 | 2.76 | 3.22 | 1.16 | 1.80 | A |
| B-19 | Profenamine | H/E/D=100/0.1/0.1 | 1.0 | 254 | 25 | 6.439 | 6.949 | 1.15 | 1.32 | 1.15 | 0.59 | C |

**TABLE S1-G** (Continued)

| Sample No. | Sample | Eluent*^a^* | Flow rate*^b^* | Detect*^b^* | Temp.*^b^* | t1 | t2 | k1 | k2 | *α* | *Rs* | Separation*^c^* |
| --- | --- | --- | --- | --- | --- | --- | --- | --- | --- | --- | --- | --- |
| B-20 | Thioridazine | H/E/D=100/1/0.1 | 1.0 | 254 | 25 | 18.536 | 20.791 | 5.18 | 5.93 | 1.15 | 2.89 | A |
| B-21 | Dimetotiazine | H/E/D=80/20/0.1 | 1.0 | 254 | 25 | 21.217 | 29.157 | 6.07 | 8.72 | 1.44 | 7.89 | A |
| B-22 | Alimemazine | H/I/D=98/2/0.1 | 1.0 | 254 | 25 | 3.953 | 4.031 | 0.32 | 0.34 | 1.08 | 0.25 | C |
| N-1 | 5-Methyl-5-phenylhydantoin | H/E=80/20 | 1.0 | 220 | 25 | 14.259 | 38.750 | 3.75 | 11.92 | 3.18 | 21.62 | A |
| N-2 | Ethotoin | H/E=80/20 | 1.0 | 200 | 25 | 12.320 | 12.731 | 3.11 | 3.24 | 1.04 | 0.81 | C |
| N-3 | γ-Phenyl-γ-butyrolactone | H/E=80/20 | 1.0 | 230 | 25 | 13.506 | 16.295 | 3.50 | 4.43 | 1.27 | 6.16 | A |
| N-4 | Pantoyl lactone | H/I=80/20 | 1.0 | 220 | 25 | 8.420 | 11.737 | 1.81 | 2.91 | 1.61 | 9.29 | A |
| N-5 | 4-Benzoyloxy-2-azetidinone | H/E=80/20 | 1.0 | 220 | 25 | 21.373 | 22.711 | 6.12 | 6.57 | 1.07 | 1.72 | B |
| B-23 | Ranolazine | H/E/D=50/50/0.1 | 1.0 | 230 | 25 | 16.531 | 27.829 | 4.51 | 8.28 | 1.83 | 8.29 | A |
| Z-1 | Cetirizine | H/E/A/D=50/50/0.1/0.1 | 1.0 | 230 | 25 | 9.788 | 12.336 | 2.26 | 3.11 | 1.38 | 3.87 | A |
| B-24 | Hydroxyzine | H/I/D=80/20/0.1 | 1.0 | 230 | 25 | 8.219 | 11.752 | 1.74 | 2.92 | 1.68 | 7.35 | A |
| B-25 | Meclizine | H/E/D=95/5/0.1 | 1.0 | 230 | 25 | 8.219 | 11.752 | 1.74 | 2.92 | 1.68 | 7.35 | A |
| B-26 | Methychlothiazide | H/E/D=60/40/0.1 | 1.0 | 230 | 25 | 13.473 | 28.568 | 3.49 | 8.52 | 2.44 | 10.93 | A |
| B-27 | Cyclopenthiazide | H/E/D=60/40/0.1 | 1.0 | 230 | 25 | 5.355 | 8.249 | 0.79 | 1.75 | 2.23 | 7.00 | A |
| B-28 | Ethiazide | H/E/D=60/40/0.1 | 1.0 | 230 | 25 | 5.806 | 9.526 | 0.94 | 2.18 | 2.33 | 8.30 | A |
| B-29 | Benzylhydrochlorothiazide | H/E/D=60/40/0.1 | 1.0 | 230 | 25 | 7.070 | 11.139 | 1.36 | 2.71 | 2.00 | 6.72 | A |
| B-30 | Mefruside | H/I/D=50/50/0.1 | 1.0 | 230 | 25 | 40.056 | 53.427 | 12.35 | 16.81 | 1.36 | 3.04 | A |
| B-31 | Polythiazide | H/E/D=60/40/0.1 | 1.0 | 230 | 25 | 7.040 | 10.594 | 1.35 | 2.53 | 1.88 | 6.33 | A |
| B-32 | Chlorprenaline | H/E/D=100/1/0.1 | 1.0 | 254 | 25 | 19.273 | 22.786 | 5.42 | 6.60 | 1.22 | 5.29 | A |
| B-33 | Tulobutenol | H/E/D=100/1/0.1 | 1.0 | 254 | 25 | 19.273 | 22.786 | 5.42 | 6.60 | 1.22 | 5.29 | A |
| B-34 | Labetalol | H/E/D=90/10/0.1 | 1.0 | 230 | 25 | 19.238 | 25.777 | 5.41 | 7.59 | 1.40 | - | C |
| B-35 | Ritodrine | H/E/D=90/10/0.1 | 1.0 | 275 | 25 | 21.644 | 26.063 | 5.93 | 7.35 | 1.24 | 4.30 | A |
| N-12 | *trans*-Stilbene oxide | H/I=90/10 | 1.0 | 254 | 25 | 5.158 | 6.966 | 0.82 | 1.45 | 1.78 | 9.42 | A |
| N-13 | 1-(9-Anthryl)-2,2,2-trifluoroethanol | H/I=90/10 | 1.0 | 254 | 25 | 5.176 | 5.596 | 0.83 | 0.97 | 1.18 | 1.55 | B |
| N-14 | Tröger’s base | H/I=90/10 | 1.0 | 254 | 25 | 6.410 | 8.464 | 1.26 | 1.99 | 1.57 | 7.37 | A |
| N-15 | Benzoin | H/I=90/10 | 1.0 | 254 | 25 | 22.009 | 23.893 | 6.76 | 7.42 | 1.10 | 2.24 | A |
| N-16 | Flavanone | H/I=90/10 | 1.0 | 254 | 25 | 9.501 | 10.235 | 2.35 | 2.61 | 1.11 | 2.09 | A |
| N-17 | 1-Naphthylethanol | H/I=90/10 | 1.0 | 254 | 25 | 7.284 | 7.549 | 1.57 | 1.66 | 1.06 | 1.23 | C |
| N-18 | Cobalt tris(acetylacetonate) | H/I=90/10 | 1.0 | 254 | 25 | 4.537 | - | 0.60 | - | 1.00 | - | D |
| N-19 | Phenyl vinyl sulfoxide | H/I=90/10 | 1.0 | 254 | 25 | 18.378 | 18.960 | 5.48 | 5.68 | 1.04 | 0.83 | C |
| N-20 | 1,1’-Bi-2-naphthol | H/I=90/10 | 1.0 | 254 | 25 | 12.915 | 13.533 | 3.55 | 3.77 | 1.06 | - | C |
| N-21 | 2-Phenylcyclohexanone | H/I=90/10 | 1.0 | 254 | 25 | 7.764 | - | 1.74 | - | 1.00 | - | D |

*^a^* “H” means *n*-hexane, “E” means ethanol, “I” means 2-propanol, “T” means trifluoroacetic acid, “D” means diethylamine and “MEA” means 2-aminoethanol.

*^b^* Flow rate’s unit is “mL/min”, Detect’s unit is “nm UV” and Temp.’s unit is “℃”.

*^c^* “A” means baseline separation, “B” means nearly baseline separation, “C” means partial separation and “D” means no separation.

**TABLE S1-K** The details of analytical conditions and chromatographic results of 67 compounds analysis with CHIRALPAK IK

| Sample No. | Sample | Eluent*^a^* | Flow rate*^b^* | Detect*^b^* | Temp.*^b^* | t1 | t2 | k1 | k2 | *α* | *Rs* | Separation*^c^* |
| --- | --- | --- | --- | --- | --- | --- | --- | --- | --- | --- | --- | --- |
| A-1 | Ketoprofen | H/E/T=93/7/0.1 | 1.0 | 254 | 25 | 21.226 | 23.417 | 6.08 | 6.81 | 1.12 | 2.90 | A |
| A-2 | Naproxen | H/E/T=97/3/0.1 | 1.0 | 254 | 25 | 16.261 | 19.828 | 4.42 | 5.61 | 1.27 | 5.30 | A |
| A-3 | Tiaprofenic acid | H/I/T=80/20/0.1 | 1.0 | 254 | 25 | 8.206 | 8.560 | 1.74 | 1.85 | 1.07 | 1.10 | C |
| A-4 | Fenoprofen | H/E/T=97/3/0.1 | 1.0 | 254 | 25 | 7.288 | 7.409 | 1.43 | 1.47 | 1.03 | - | C |
| A-5 | Flurbiprofen | H/E/T=97/3/0.1 | 1.0 | 254 | 25 | 7.388 | 7.485 | 1.46 | 1.50 | 1.02 | - | C |
| A-6 | 2-Phenylpropanoic acid | H/I/T=95/5/0.1 | 1.0 | 230 | 25 | 5.780 | 5.951 | 0.93 | 0.98 | 1.06 | - | C |
| A-7 | Ibuprofen | H/I/T=98/2/0.1 | 1.0 | 200 | 25 | 6.954 | 7.408 | 1.32 | 1.47 | 1.11 | 1.90 | B |
| A-8 | Pranoprofen | H/I/T=90/10/0.1 | 1.0 | 200 | 25 | 28.751 | 31.407 | 8.58 | 9.47 | 1.10 | 1.90 | B |
| A-9 | 2-Phenoxypropanoic acid | H/E/T=97/3/0.1 | 1.0 | 230 | 25 | 7.542 | 7.542 | 1.51 | 1.51 | 1.00 | - | D |
| A-10 | 2-(*o*-Chlorophenoxy)propanoic acid | H/I/T=95/5/0.1 | 1.0 | 230 | 25 | 5.544 | 5.662 | 0.85 | 0.89 | 1.05 | - | C |
| A-11 | 2-(4-Hydroxyphenoxy)propanoic acid | H/I/T=90/10/0.1 | 1.0 | 230 | 25 | 10.792 | 14.676 | 2.60 | 3.89 | 1.50 | 6.50 | A |
| A-12 | N-Cbz-Leucine | H/I/T=90/10/0.1 | 1.0 | 200 | 25 | 10.493 | 12.433 | 2.50 | 3.14 | 1.26 | 3.00 | A |
| A-13 | N-Cbz-Valine | H/E/T=90/10/0.1 | 1.0 | 230 | 25 | 6.956 | 7.374 | 1.32 | 1.46 | 1.11 | 1.50 | B |
| A-14 | N-Cbz-Methionine | H/E/T=90/10/0.1 | 1.0 | 230 | 25 | 10.795 | 11.010 | 2.60 | 2.67 | 1.03 | - | C |
| A-15 | N-Cbz-Norleuicine | H/I/T=90/10/0.1 | 1.0 | 230 | 25 | 10.656 | 11.149 | 2.55 | 2.72 | 1.06 | 1.00 | C |
| A-16 | N-Cbz-Norvaline | H/I/T=90/10/0.1 | 1.0 | 230 | 25 | 11.022 | 11.561 | 2.67 | 2.85 | 1.07 | 1.10 | C |
| B-1 | Propranolol | H/I/D=90/10/0.1 | 1.0 | 230 | 25 | 7.488 | 9.887 | 1.50 | 2.30 | 1.53 | 8.40 | A |
| B-2 | Propafenone | H/I/D=80/20/0.1 | 1.0 | 230 | 25 | 8.363 | 8.649 | 1.79 | 1.88 | 1.05 | - | C |
| B-3 | Oxprenolol | H/I/D=90/10/0.1 | 1.0 | 230 | 25 | 7.616 | 11.140 | 1.54 | 2.71 | 1.76 | 12.00 | A |
| B-4 | Alprenolol | H/I/D=95/5/0.1 | 1.0 | 230 | 25 | 5.947 | 6.155 | 0.98 | 1.05 | 1.07 | 1.20 | C |
| B-5 | Pindolol | H/I/D=80/20/0.1 | 1.0 | 230 | 25 | 10.388 | 15.523 | 2.46 | 4.17 | 1.70 | 9.80 | A |
| B-6 | Atenolol | H/I/D=70/30/0.1 | 1.0 | 230 | 25 | 25.161 | 26.535 | 7.39 | 7.85 | 1.06 | 1.10 | C |
| B-7 | Nitrendipine | H/I/D=80/20/0.1 | 1.0 | 254 | 40 | 5.672 | 5.732 | 0.88 | 0.90 | 1.02 | - | C |
| B-8 | Manidipine | H/I/D=80/20/0.1 | 1.0 | 254 | 40 | 7.098 | - | 1.35 | - | 1.00 | - | D |
| B-9 | Benidipine | H/I/D=90/10/0.1 | 1.0 | 254 | 40 | 11.278 | - | 2.69 | - | 1.00 | - | D |
| B-10 | Nilvadipine | H/I/D=90/10/0.1 | 1.0 | 254 | 40 | 11.912 | 13.375 | 2.90 | 3.37 | 1.17 | 3.53 | A |
| B-11 | Nicardipine | H/E/D=95/5/0.1 | 1.0 | 254 | 40 | 15.893 | 16.48 | 3.88 | 4.06 | 1.05 | 1.14 | C |
| B-12 | Amlodipine | H/I/MEA=80/20/0.1 | 1.0 | 254 | 40 | 17.222 | 17.789 | 4.65 | 4.83 | 1.04 | 0.92 | C |
| B-13 | Nisoldipine | H/I/D=90/10/0.1 | 1.0 | 254 | 40 | 11.809 | 13.325 | 2.86 | 3.36 | 1.17 | 3.61 | A |
| B-14 | Nimodipine | H/I/D=90/10/0.1 | 1.0 | 254 | 40 | 11.664 | 14.163 | 2.81 | 3.63 | 1.29 | 5.86 | A |
| B-15 | Cisapride | H/I/D=70/30/0.1 | 1.0 | 254 | 25 | 12.674 | 13.916 | 3.22 | 3.64 | 1.13 | 2.10 | A |
| B-16 | Sulpiride | H/I/D=50/50/0.1 | 1.0 | 230 | 25 | 17.146 | 17.146 | 4.72 | 4.72 | 1.00 | - | D |
| B-17 | Troxipide | H/I/D=60/40/0.1 | 1.0 | 230 | 25 | 6.814 | 16.458 | 1.27 | 4.49 | 3.53 | 19.60 | A |
| B-18 | Indapamide | H/I/D=50/50/0.1 | 1.0 | 230 | 25 | 6.980 | 7.920 | 1.33 | 1.64 | 1.24 | 2.40 | A |
| B-19 | Profenamine | H/I/D=98/2/0.1 | 1.0 | 254 | 25 | 4.225 | 4.225 | 0.41 | 0.41 | 1.00 | - | D |

**TABLE S1-K** (Continued)

| Sample No. | Sample | Eluent*^a^* | Flow rate*^b^* | Detect*^b^* | Temp.*^b^* | t1 | t2 | k1 | k2 | *α* | *Rs* | Separation*^c^* |
| --- | --- | --- | --- | --- | --- | --- | --- | --- | --- | --- | --- | --- |
| B-20 | Thioridazine | H/I/D=98/2/0.1 | 1.0 | 254 | 25 | 7.641 | 7.641 | 1.55 | 1.55 | 1.00 | - | D |
| B-21 | Dimetotiazine | H/I/D=70/30/0.1 | 1.0 | 254 | 25 | 11.224 | 11.563 | 2.74 | 2.85 | 1.04 | - | C |
| B-22 | Alimemazine | H/I/D=98/2/0.1 | 1.0 | 254 | 25 | 4.622 | 4.821 | 0.54 | 0.61 | 1.12 | 1.30 | C |
| N-1 | 5-Methyl-5-phenylhydantoin | H/I=90/10 | 1.0 | 220 | 25 | 11.549 | 13.035 | 2.85 | 3.35 | 1.17 | 3.00 | A |
| N-2 | Ethotoin | H/I=90/10 | 1.0 | 200 | 25 | 15.827 | 17.994 | 4.28 | 5.00 | 1.17 | 3.80 | A |
| N-3 | γ-Phenyl-γ-butyrolactone | H/I=90/10 | 1.0 | 230 | 25 | 23.685 | 25.113 | 6.90 | 7.37 | 1.07 | 2.10 | A |
| N-4 | Pantoyl lactone | H/I=80/20 | 1.0 | 220 | 25 | 10.068 | 11.341 | 2.36 | 2.78 | 1.18 | 3.80 | A |
| N-5 | 4-Benzoyloxy-2-azetidinone | H/I=60/40 | 1.0 | 220 | 25 | 7.495 | 10.532 | 1.50 | 2.51 | 1.68 | 9.20 | A |
| B-23 | Ranolazine | H/I/D=50/50/0.1 | 1.0 | 230 | 25 | 23.601 | 35.676 | 6.87 | 10.89 | 1.59 | 5.90 | A |
| Z-1 | Cetirizine | H/I/A/D=50/50/0.1/0.1 | 1.0 | 230 | 25 | 15.010 | 16.269 | 4.00 | 4.42 | 1.10 | 1.60 | B |
| B-24 | Hydroxyzine | H/E/D=90/10/0.1 | 1.0 | 230 | 25 | 7.323 | 7.600 | 1.44 | 1.53 | 1.06 | 1.20 | C |
| B-25 | Meclizine | H/I/D=95/5/0.1 | 1.0 | 230 | 25 | 3.930 | - | 0.31 | - | 1.00 | - | D |
| B-26 | Methychlothiazide | H/I/D=70/30/0.1 | 1.0 | 230 | 25 | 12.920 | 14.527 | 3.31 | 3.84 | 1.16 | 2.30 | A |
| B-27 | Cyclopenthiazide | H/I/D=70/30/0.1 | 1.0 | 230 | 25 | 8.842 | 12.457 | 1.95 | 3.15 | 1.62 | 5.40 | A |
| B-28 | Ethiazide | H/I/D=70/30/0.1 | 1.0 | 230 | 25 | 8.873 | 13.197 | 1.96 | 3.40 | 1.74 | 6.70 | A |
| B-29 | Benzylhydrochlorothiazide | H/I/D=70/30/0.1 | 1.0 | 230 | 25 | 13.155 | 20.549 | 3.39 | 5.85 | 1.73 | 6.50 | A |
| B-30 | Mefruside | H/E/D=50/50/0.1 | 1.0 | 230 | 25 | 4.985 | 5.025 | 0.66 | 0.68 | 1.02 | - | C |
| B-31 | Polythiazide | H/I/D=70/30/0.1 | 1.0 | 230 | 25 | 10.025 | 11.490 | 2.34 | 2.83 | 1.21 | 2.50 | A |
| B-32 | Chlorprenaline | H/E/D=100/1/0.1 | 1.0 | 254 | 25 | 8.568 | 9.701 | 1.86 | 2.23 | 1.20 | 3.72 | A |
| B-33 | Tulobutenol | H/E/D=100/1/0.1 | 1.0 | 264 | 25 | 6.499 | 7.752 | 1.17 | 1.58 | 1.36 | 4.50 | A |
| B-34 | Labetalol | H/I/D=90/10/0.1 | 1.0 | 230 | 25 | 10.546 | 11.108 | 2.52 | 2.70 | 1.07 | - | C |
| B-35 | Ritodrine | H/I/D=90/10/0.1 | 1.0 | 275 | 25 | 41.588 | 45.972 | 12.86 | 14.32 | 1.11 | 1.60 | B |
| N-12 | *trans*-Stilbene oxide | H/I=90/10 | 1.0 | 254 | 25 | 4.664 | 6.226 | 0.56 | 1.08 | 1.94 | 10.62 | A |
| N-13 | 1-(9-Anthryl)-2,2,2-trifluoroethanol | H/I=90/10 | 1.0 | 254 | 25 | 4.911 | 6.037 | 0.64 | 1.02 | 1.59 | 4.71 | A |
| N-14 | Tröger’s base | H/I=90/10 | 1.0 | 254 | 25 | 5.375 | 7.285 | 0.80 | 1.44 | 1.80 | 10.70 | A |
| N-15 | Benzoin | H/I=90/10 | 1.0 | 254 | 25 | 12.588 | 12.761 | 3.21 | 3.27 | 1.02 | - | C |
| N-16 | Flavanone | H/I=90/10 | 1.0 | 254 | 25 | 7.348 | 8.255 | 1.46 | 1.76 | 1.21 | 4.42 | A |
| N-17 | 1-Naphthylethanol | H/I=90/10 | 1.0 | 254 | 25 | 6.959 | 8.026 | 1.33 | 1.69 | 1.27 | 5.14 | A |
| N-18 | Cobalt tris(acetylacetonate) | H/I=90/10 | 1.0 | 254 | 25 | 4.658 | 5.711 | 0.56 | 0.91 | 1.63 | 7.15 | A |
| N-19 | Phenyl vinyl sulfoxide | H/I=90/10 | 1.0 | 254 | 25 | 21.817 | 22.116 | 6.30 | 6.40 | 1.02 | - | C |
| N-20 | 1,1’-Bi-2-naphthol | H/I=90/10 | 1.0 | 254 | 25 | 12.535 | 16.655 | 3.19 | 4.57 | 1.43 | 7.94 | A |
| N-21 | 2-Phenylcyclohexanone | H/I=90/10 | 1.0 | 254 | 25 | 9.034 | 11.049 | 2.02 | 2.70 | 1.33 | 7.14 | A |

*^a^* “H” means *n*-hexane, “E” means ethanol, “I” means 2-propanol, “T” means trifluoroacetic acid, “D” means diethylamine and “MEA” means 2-aminoethanol.

*^b^* Flow rate’s unit is “mL/min”, Detect’s unit is “nm UV” and Temp.’s unit is “℃”.

*^c^* “A” means baseline separation, “B” means nearly baseline separation, “C” means partial separation and “D” means no separation.

**FIGURE S2** Representative separation chromatograms with CHIRALPAK IG and IK. The analytical conditions except eluent are shown in Table S1-G and S1-K.
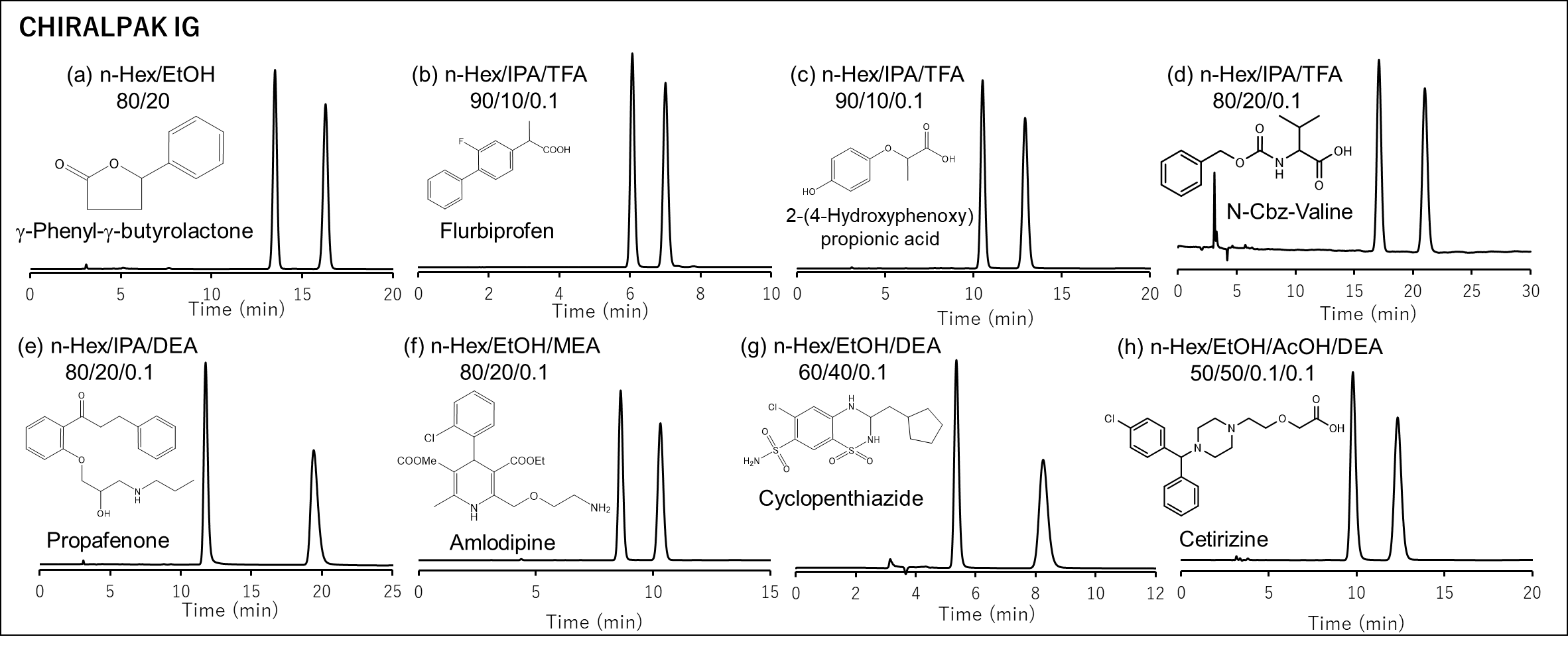


**FIGURE S2** (Continued)


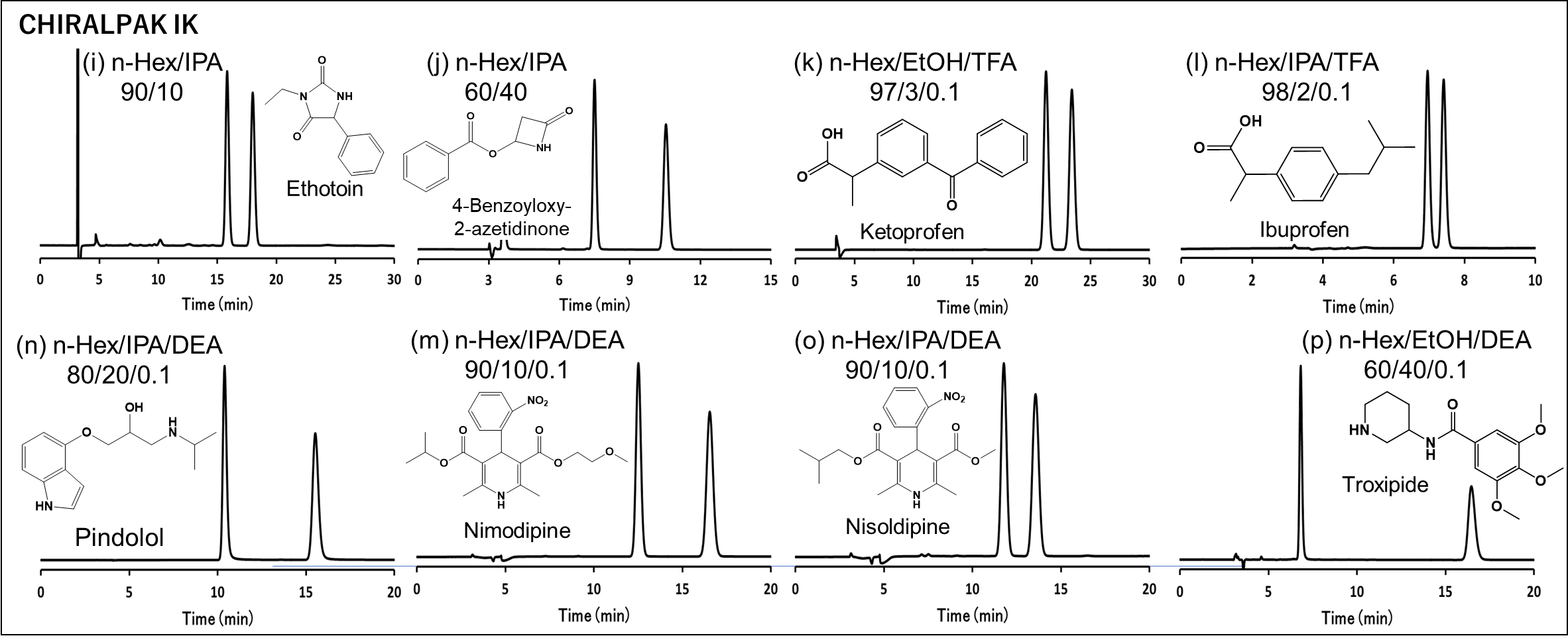


**TABLE S2-A** The details of analytical conditions and chromatographic results of 50 compounds analysis with CHIRALPAK AS-H

| Sample No. | Sample | Eluent*^a^* | Flow rate*^b^* | Detect*^b^* | Temp.*^b^* | t1 | t2 | k1 | k2 | *α* | *Rs* |
| --- | --- | --- | --- | --- | --- | --- | --- | --- | --- | --- | --- |
| N-12 | *trans*-Stilbene oxide | H/I=90/10 | 1.0 | 254 | 25 | 4.478 | 5.009 | 0.55 | 0.73 | 1.34 | 4.10 |
| N-13 | 1-(9-Anthryl)-2,2,2-trifluoroethanol | H/I=90/10 | 1.0 | 254 | 25 | 7.345 | 12.543 | 1.54 | 3.34 | 2.17 | 10.70 |
| N-14 | Tröger's base | H/I=90/10 | 1.0 | 254 | 25 | 5.115 | 8.283 | 0.77 | 1.86 | 2.43 | 9.41 |
| N-15 | Benzoin | H/I=90/10 | 1.0 | 254 | 25 | 13.285 | 25.734 | 3.59 | 7.88 | 2.20 | 16.99 |
| N-16 | Flavanone | H/I=90/10 | 1.0 | 254 | 25 | 11.573 | - | 2.99 | - | 1.00 | - |
| N-17 | 1-Naphthylethanol | H/I=90/10 | 1.0 | 254 | 25 | 6.335 | 6.629 | 1.19 | 1.29 | 1.09 | 1.41 |
| N-18 | Cobalt tris(acetylacetonate) | H/I=90/10 | 1.0 | 254 | 25 | 3.982 | 4.165 | 0.37 | 0.44 | 1.17 | 1.03 |
| N-19 | Phenyn vinyl sulfoxide | H/I=90/10 | 1.0 | 254 | 25 | 27.811 | 40.531 | 8.59 | 12.98 | 1.51 | 10.20 |
| N-20 | 1,1'-Bi-2-naphthol | H/I=90/10 | 1.0 | 254 | 25 | 11.868 | 17.852 | 3.09 | 5.16 | 1.67 | 6.78 |
| N-21 | 2-Phenylcyclohexanone | H/I=90/10 | 1.0 | 254 | 25 | 7.004 | 8.083 | 1.41 | 1.79 | 1.26 | 4.51 |
| N-2 | Ethotoin | H/I=50/50 | 1.0 | 210 | 25 | 7.543 | 28.221 | 1.42 | 8.04 | 5.68 | 14.05 |
| N-5 | 4-Benzoyloxy-2-azetidinone | H/I=70/30 | 1.0 | 230 | 25 | 16.898 | 23.575 | 4.41 | 6.55 | 1.48 | 6.66 |
| N-1 | 5-Methyl-5-phenylhydantoin | H/I=70/30 | 1.0 | 210 | 25 | 7.775 | 15.778 | 1.49 | 4.05 | 2.72 | 8.60 |
| N-3 | γ-Phenyl-γ-butyrolactone | H/I=80/20 | 1.0 | 210 | 25 | 15.639 | 19.048 | 4.01 | 5.10 | 1.27 | 5.51 |
| N-6 | Guaifenesin | H/I=80/20 | 1.0 | 275 | 25 | 7.901 | 8.162 | 1.53 | 1.61 | 1.05 | - |
| N-4 | Pantoyl lactone | H/I=80/20 | 1.0 | 220 | 25 | 11.69 | 16.43 | 2.74 | 4.26 | 1.55 | 7.91 |
| N-7 | 2-Methyl-1-tetralone | H/I=90/10 | 1.0 | 280 | 25 | 4.587 | 4.772 | 0.47 | 0.53 | 1.13 | 1.52 |
| N-8 | Ethylmandelate | H/I=90/10 | 1.0 | 210 | 25 | 8.864 | 11.268 | 1.84 | 2.61 | 1.42 | 6.69 |
| N-9 | 1-Phenoxy-2-propanol | H/I=90/10 | 1.0 | 220 | 25 | 6.030 | 6.598 | 0.93 | 1.11 | 1.20 | 2.72 |
| N-10 | 1-Phenylethyl alcohol | H/I=90/10 | 1.0 | 210 | 25 | 5.198 | 5.328 | 0.66 | 0.71 | 1.06 | 0.83 |
| N-11 | Benzoin ethyl ether | H/I=95/5 | 1.0 | 254 | 25 | 5.296 | 6.799 | 0.70 | 1.18 | 1.69 | 7.37 |
| N-22 | 4-Ethenyl-2-azetidinone | H/I=60/40 | 1.0 | 210 | 25 | 16.311 | 22.877 | 4.22 | 6.33 | 1.50 | 6.77 |
| N-23 | 1-[*tert-*Butyl(dimethyl)silyl]-4-methyl-2-azetidinone | H/I=60/40 | 1.0 | 210 | 25 | 3.806 | 4.774 | 0.22 | 0.53 | 2.42 | 5.34 |
| N-24 | 1-Benzyl-3-ethyl-2-azetidinone | H/I=60/40 | 1.0 | 210 | 25 | 10.414 | 16.454 | 2.34 | 4.27 | 1.83 | 8.99 |
| N-25 | 3-Dimethyl-4-phenyl-2-azetidinone | H/E=90/10 | 1.0 | 210 | 25 | 25.741 | 29.377 | 7.25 | 8.41 | 1.16 | 3.22 |
| N-26 | 3-Dimethyl-4-(2-furyl)-2-azetidinone | H/I=60/40 | 1.0 | 210 | 25 | 16.274 | 46.712 | 4.21 | 13.96 | 3.31 | 14.97 |
| A-17 | Warfarin | H/I/T=50/50/0.1 | 1.0 | 280 | 25 | 4.889 | 9.173 | 0.57 | 1.94 | 3.42 | 3.38 |
| A-14 | N-Cbz-Methionine | H/I/T=50/50/0.1 | 1.0 | 210 | 25 | 5.353 | 12.374 | 0.71 | 2.96 | 4.15 | 8.30 |
| A-18 | N-Cbz-Alanine | H/I/T=80/20/0.1 | 1.0 | 210 | 25 | 7.956 | 14.661 | 1.55 | 3.70 | 2.39 | 10.60 |
| A-13 | N-Cbz-Valine | H/I/T=80/20/0.1 | 1.0 | 210 | 25 | 6.046 | 21.504 | 0.94 | 5.89 | 6.29 | 16.32 |
| A-15 | N-Cbz-Norleuicine | H/I/T=80/20/0.1 | 1.0 | 210 | 25 | 6.771 | 25.807 | 1.17 | 7.27 | 6.22 | 13.98 |
| A-16 | N-Cbz-Norvaline | H/I/T=80/20/0.1 | 1.0 | 210 | 25 | 6.799 | 32.117 | 1.18 | 9.29 | 7.89 | 16.68 |
| A-11 | 2-(4-Hydroxyphenoxy)propionic acid | H/I/T=90/10/0.1 | 1.0 | 290 | 25 | 15.266 | 18.468 | 3.89 | 4.92 | 1.26 | 3.89 |
| A-2 | Naproxen | H/I/T=90/10/0.1 | 1.0 | 230 | 25 | 11.803 | 14.584 | 2.78 | 3.67 | 1.32 | 2.42 |
| A-5 | Flurbiprofen | H/I/T=90/10/0.1 | 1.0 | 254 | 25 | 5.458 | 5.702 | 0.75 | 0.83 | 1.10 | 1.20 |
| A-9 | 2-Phenoxypropionic acid | H/I/T=98/2/0.1 | 1.0 | 254 | 25 | 13.577 | 16.487 | 3.35 | 4.28 | 1.28 | 5.24 |
| A-6 | 2-Phenylpropionic acid | H/I/T=98/2/0.1 | 1.0 | 254 | 25 | 13.288 | - | 3.26 | - | 1.00 | - |
| B-36 | Aminoglutethimide | H/I/D=50/50/0.1 | 1.0 | 290 | 25 | 15.991 | 50.756 | 4.12 | 15.26 | 3.70 | 12.98 |
| B-16 | Sulpiride | H/I/D=70/30/0.1 | 1.0 | 230 | 25 | 22.732 | 27.059 | 6.28 | 7.67 | 1.22 | 1.59 |

**TABLE S2-A** (Continued)

| Sample No. | Sample | Eluent*^a^* | Flow rate*^b^* | Detect*^b^* | Temp.*^b^* | t1 | t2 | k1 | k2 | *α* | *Rs* |
| --- | --- | --- | --- | --- | --- | --- | --- | --- | --- | --- | --- |
| B-37 | Sotalol HCl | H/I/D=70/30/0.1 | 1.0 | 210 | 25 | 7.699 | 8.413 | 1.47 | 1.69 | 1.16 | 1.22 |
| B-38 | Ofloxacine Et ester | H/I/D=70/30/0.1 | 1.0 | 210 | 25 | 22.259 | 34.103 | 6.13 | 9.92 | 1.62 | 3.20 |
| B-39 | Diltiazem | H/I/D=80/20/0.1 | 1.0 | 254 | 25 | 8.724 | - | 1.79 | - | 1.00 | - |
| B-40 | Disopyramide | H/I/D=90/10/0.1 | 1.0 | 254 | 25 | 10.887 | 14.47 | 2.49 | 3.63 | 1.46 | 3.10 |
| B-2 | Propafenone | H/I/D=90/10/0.1 | 1.0 | 230 | 25 | 9.537 | 12.743 | 2.05 | 3.08 | 1.50 | 4.21 |
| B-5 | Pindolol | H/I/D=90/10/0.1 | 1.0 | 260 | 25 | 14.054 | 14.953 | 3.50 | 3.79 | 1.08 | 1.12 |
| B-9 | Benidipine | H/I/D=90/10/0.1 | 1.0 | 254 | 25 | 8.998 | 10.268 | 1.88 | 2.29 | 1.22 | 1.11 |
| B-40 | Alprenolol | H/I/D=90/10/0.1 | 1.0 | 270 | 25 | 3.857 | 3.973 | 0.24 | 0.27 | 1.16 | 0.87 |
| B-24 | Hydroxyzine | H/I/D=90/10/0.1 | 1.0 | 230 | 25 | 7.974 | 8.917 | 1.55 | 1.86 | 1.19 | 1.33 |
| B-41 | Verapamil HCl | H/I/D=90/10/0.1 | 1.0 | 280 | 25 | 7.57 | 8.174 | 1.42 | 1.62 | 1.14 | 1.16 |
| Z-2 | Ofloxacine | H/E/AcOH/EDA=70/30/0.1/0.1 | 1.0 | 290 | 25 | 10.365 | 16.903 | 2.32 | 4.41 | 1.90 | 6.16 |

*^a^* “H” means *n*-hexane, “E” means ethanol, “I” means 2-propanol, “T” means trifluoroacetic acid, “D” means diethylamine, “AcOH” means acetic acid and “EDA” means ethylenediamine.

*^b^* Flow rate’s unit is “mL/min”, Detect’s unit is “nm UV” and Temp.’s unit is “℃”.

**TABLE S2-B** The details of analytical conditions and chromatographic results of 50 compounds analysis with CHIRALPAK IH

| Sample No. | Sample | Eluent*^a^* | Flow rate*^b^* | Detect*^b^* | Temp.*^b^* | t1 | t2 | k1 | k2 | *α* | *Rs* |
| --- | --- | --- | --- | --- | --- | --- | --- | --- | --- | --- | --- |
| N-12 | *trans*-Stilbene oxide | H/I=90/10 | 1.0 | 254 | 25 | 4.689 | 5.156 | 0.59 | 0.75 | 1.27 | 3.56 |
| N-13 | 1-(9-Anthryl)-2,2,2-trifluoroethanol | H/I=90/10 | 1.0 | 254 | 25 | 7.492 | 11.888 | 1.54 | 3.03 | 1.97 | 10.73 |
| N-14 | Tröger's base | H/I=90/10 | 1.0 | 254 | 25 | 5.46 | 8.763 | 0.85 | 1.97 | 2.32 | 8.40 |
| N-15 | Benzoin | H/I=90/10 | 1.0 | 254 | 25 | 12.933 | 21.117 | 3.38 | 6.15 | 1.82 | 14.08 |
| N-16 | Flavanone | H/I=90/10 | 1.0 | 254 | 25 | 10.796 | - | 2.65 | - | 1.00 | - |
| N-17 | 1-Naphthylethanol | H/I=90/10 | 1.0 | 254 | 25 | 6.329 | 6.68 | 1.14 | 1.26 | 1.10 | 1.86 |
| N-18 | Cobalt tris(acetylacetonate) | H/I=90/10 | 1.0 | 254 | 25 | 4.477 | 4.718 | 0.52 | 0.60 | 1.16 | 1.37 |
| N-19 | Phenyn vinyl sulfoxide | H/I=90/10 | 1.0 | 254 | 25 | 25.001 | 35.736 | 7.47 | 11.10 | 1.49 | 9.18 |
| N-20 | 1,1'-Bi-2-naphthol | H/I=90/10 | 1.0 | 254 | 25 | 12.781 | 17.226 | 3.33 | 4.83 | 1.45 | 5.29 |
| N-21 | 2-Phenylcyclohexanone | H/I=90/10 | 1.0 | 254 | 25 | 6.816 | 7.636 | 1.31 | 1.59 | 1.21 | 3.67 |
| N-2 | Ethotoin | H/I=50/50 | 1.0 | 210 | 25 | 6.679 | 18.806 | 1.21 | 5.21 | 4.32 | 11.29 |
| N-5 | 4-Benzoyloxy-2-azetidinone | H/I=70/30 | 1.0 | 230 | 25 | 17.725 | 20.615 | 4.86 | 5.81 | 1.20 | 3.07 |
| N-1 | 5-Methyl-5-phenylhydantoin | H/I=70/30 | 1.0 | 210 | 25 | 7.667 | 13.595 | 1.53 | 3.49 | 2.28 | 7.91 |
| N-3 | γ-Phenyl-γ-butyrolactone | H/I=80/20 | 1.0 | 210 | 25 | 14.759 | 17.421 | 3.88 | 4.76 | 1.23 | 4.19 |
| N-6 | Guaifenesin | H/I=80/20 | 1.0 | 275 | 25 | 7.761 | 8.036 | 1.56 | 1.66 | 1.06 | 0.89 |
| N-4 | Pantoyl lactone | H/I=80/20 | 1.0 | 220 | 25 | 11.677 | 13.932 | 2.86 | 3.60 | 1.26 | 3.79 |
| N-7 | 2-Methyl-1-tetralone | H/I=90/10 | 1.0 | 280 | 25 | 4.698 | 4.844 | 0.55 | 0.60 | 1.09 | 1.21 |
| N-8 | Ethylmandelate | H/I=90/10 | 1.0 | 210 | 25 | 8.276 | 10.25 | 1.73 | 2.39 | 1.38 | 5.59 |
| N-9 | 1-Phenoxy-2-propanol | H/I=90/10 | 1.0 | 220 | 25 | 5.978 | 6.527 | 0.98 | 1.16 | 1.19 | 2.76 |
| N-10 | 1-Phenylethyl alcohol | H/I=90/10 | 1.0 | 210 | 25 | 5.204 | 5.287 | 0.72 | 0.75 | 1.04 | - |
| N-11 | Benzoin ethyl ether | H/I=95/5 | 1.0 | 254 | 25 | 5.385 | 6.496 | 0.78 | 1.15 | 1.47 | 5.99 |
| N-22 | 4-Ethenyl-2-azetidinone | H/I=60/40 | 1.0 | 210 | 25 | 13.445 | 18.031 | 3.44 | 4.96 | 1.44 | 6.11 |
| N-23 | 1-[*tert*-Butyl(dimethyl)silyl]-4-methyl-2-azetidinone | H/I=60/40 | 1.0 | 210 | 25 | 3.727 | 4.425 | 0.23 | 0.46 | 2.00 | 4.97 |
| N-24 | 1-Benzyl-3-ethyl-2-azetidinone | H/I=60/40 | 1.0 | 210 | 25 | 8.966 | 12.909 | 1.96 | 3.27 | 1.66 | 8.30 |
| N-25 | 3-Dimethyl-4-phenyl-2-azetidinone | H/E=90/10 | 1.0 | 210 | 25 | 24.01 | 27.272 | 6.93 | 8.01 | 1.16 | 3.35 |
| N-26 | 3-Dimethyl-4-(2-furyl)-2-azetidinone | H/I=60/40 | 1.0 | 210 | 25 | 12.776 | 35.996 | 3.22 | 10.90 | 3.38 | 17.89 |
| A-17 | Warfarin | H/I/T=50/50/0.1 | 1.0 | 280 | 25 | 4.844 | 9.178 | 0.60 | 2.03 | 3.38 | 3.77 |
| A-14 | N-Cbz-Methionine | H/I/T=50/50/0.1 | 1.0 | 210 | 25 | 5.67 | 8.578 | 0.87 | 1.83 | 2.10 | 4.97 |
| A-18 | N-Cbz-Alanine | H/I/T=80/20/0.1 | 1.0 | 210 | 25 | 7.696 | 10.888 | 1.54 | 2.60 | 1.68 | 6.95 |
| A-13 | N-CBZ-Valine | H/I/T=80/20/0.1 | 1.0 | 210 | 25 | 5.872 | 12.319 | 0.94 | 3.07 | 3.27 | 14.39 |
| A-15 | N-Cbz-Norleuicine | H/I/T=80/20/0.1 | 1.0 | 210 | 25 | 6.808 | 14.527 | 1.25 | 3.80 | 3.04 | 10.10 |
| A-16 | N-Cbz-Norvaline | H/I/T=80/20/0.1 | 1.0 | 210 | 25 | 6.942 | 17.549 | 1.29 | 4.80 | 3.71 | 13.70 |
| A-11 | 2-(4-Hydroxyphenoxy)propionic acid | H/I/T=90/10/0.1 | 1.0 | 290 | 25 | 14.516 | 16.124 | 3.80 | 4.33 | 1.14 | 2.42 |
| A-2 | Naproxen | H/I/T=90/10/0.1 | 1.0 | 230 | 25 | 12.419 | 15.878 | 3.10 | 4.25 | 1.37 | 2.29 |
| A-5 | Flurbiprofen | H/I/T=90/10/0.1 | 1.0 | 254 | 25 | 5.487 | 5.621 | 0.81 | 0.86 | 1.05 | - |
| A-9 | 2-Phenoxypropionic acid | H/I/T=98/2/0.1 | 1.0 | 254 | 25 | 13.091 | 15.441 | 3.33 | 4.10 | 1.23 | 4.64 |
| A-6 | 2-Phenylpropionic acid | H/I/T=98/2/0.1 | 1.0 | 254 | 25 | 12.205 | - | 3.03 | - | 1.00 | - |
| B-36 | Aminoglutethimide | H/I/D=50/50/0.1 | 1.0 | 290 | 25 | 16.147 | 47.482 | 4.34 | 14.69 | 3.39 | 11.46 |
| B-16 | Sulpiride | H/I/D=70/30/0.1 | 1.0 | 230 | 25 | 32.208 | 39.334 | 9.64 | 12.00 | 1.24 | 1.71 |

**TABLE S2-B** (Continued)

| Sample No. | Sample | Eluent*^a^* | Flow rate*^b^* | Detect*^b^* | Temp.*^b^* | t1 | t2 | k1 | k2 | *α* | *Rs* |
| --- | --- | --- | --- | --- | --- | --- | --- | --- | --- | --- | --- |
| B-37 | Sotalol HCl | H/I/D=70/30/0.1 | 1.0 | 210 | 25 | 8.853 | 9.801 | 1.93 | 2.24 | 1.16 | 1.48 |
| B-38 | Ofloxacine Et ester | H/I/D=70/30/0.1 | 1.0 | 210 | 25 | 25.124 | 39.742 | 7.30 | 12.13 | 1.66 | 3.71 |
| B-39 | Diltiazem | H/I/D=80/20/0.1 | 1.0 | 254 | 25 | 8.974 | - | 1.97 | - | 1 | - |
| B-40 | Disopyramide | H/I/D=90/10/0.1 | 1.0 | 254 | 25 | 11.792 | 14.227 | 2.90 | 3.70 | 1.28 | 2.43 |
| B-2 | Propafenone | H/I/D=90/10/0.1 | 1.0 | 230 | 25 | 9.894 | 10.218 | 2.27 | 2.38 | 1.05 | 0.00 |
| B-5 | Pindolol | H/I/D=90/10/0.1 | 1.0 | 260 | 25 | 14.808 | 15.967 | 3.89 | 4.28 | 1.10 | 1.47 |
| B-9 | Benidipine | H/I/D=90/10/0.1 | 1.0 | 254 | 25 | 11.641 | 12.677 | 2.85 | 3.19 | 1.12 | 0.89 |
| B-40 | Alprenolol | H/I/D=90/10/0.1 | 1.0 | 270 | 25 | 4.018 | 4.129 | 0.33 | 0.36 | 1.11 | 0.93 |
| B-24 | Hydroxyzine | H/I/D=90/10/0.1 | 1.0 | 230 | 25 | 7.756 | 9.092 | 1.56 | 2.00 | 1.28 | 1.79 |
| B-41 | Verapamil HCl | H/I/D=90/10/0.1 | 1.0 | 280 | 25 | 8.565 | 9.057 | 1.83 | 1.99 | 1.09 | 0.94 |
| Z-2 | Ofloxacine | H/E/AcOH/EDA=70/30/0.1/0.1 | 1.0 | 290 | 25 | 12.589 | 17.246 | 3.16 | 4.70 | 1.49 | 4.83 |

*^a^* “H” means *n*-hexane, “E” means ethanol, “I” means 2-propanol, “T” means trifluoroacetic acid, “D” means diethylamine, “AcOH” means acetic acid and “EDA” means ethylenediamine.

*^b^* Flow rate’s unit is “mL/min”, Detect’s unit is “nm UV” and Temp.’s unit is “℃”.

**TABLE S3** The results of separation of 8 kinds of β-lactone or β-lactam compounds on 9 kinds of CSPs.

| Column | Sample No. | Sample | Eluent*^a^* | Flow rate*^b^* | Detect*^b^* | Temp.*^b^* | t1 | t2 | k1 | k2 | *α* | *Rs* |
| --- | --- | --- | --- | --- | --- | --- | --- | --- | --- | --- | --- | --- |
| IA | N-22 | 4-Ethenyl-2-azetidinone | H/I=60/40 | 1.0 | 210 | 25 | 3.855 | - | 0.34 | - | 1.00 | - |
| IA | N-23 | 1-[*tert-*Butyl(dimethyl)silyl]-4-methyl-2-azetidinone | H/I=60/40 | 1.0 | 210 | 25 | 3.213 | - | 0.11 | - | 1.00 | - |
| IA | N-24 | 1-Benzyl-3-ethyl-2-azetidinone | H/I=60/40 | 1.0 | 210 | 25 | 3.937 | 4.039 | 0.36 | 0.40 | 1.10 | - |
| IA | N-25 | 3-Dimethyl-4-phenyl-2-azetidinone | H/E=90/10 | 1.0 | 210 | 25 | 10.21 | 10.371 | 2.54 | 2.59 | 1.02 | - |
| IA | N-26 | 3-Dimethyl-4-(2-furyl)-2-azetidinone | H/I=60/40 | 1.0 | 210 | 25 | 3.691 | 4.173 | 0.28 | 0.45 | 1.60 | 4.53 |
| IB N | N-22 | 4-Ethenyl-2-azetidinone | H/I=60/40 | 1.0 | 210 | 25 | 4.178 | 4.416 | 0.40 | 0.48 | 1.20 | 1.91 |
| IB N | N-23 | 1-[*tert-*Butyl(dimethyl)silyl]-4-methyl-2-azetidinone | H/I=60/40 | 1.0 | 210 | 25 | 3.422 | 3.490 | 0.15 | 0.17 | 1.16 | - |
| IB N | N-24 | 1-Benzyl-3-ethyl-2-azetidinone | H/I=60/40 | 1.0 | 210 | 25 | 4.423 | 4.531 | 0.48 | 0.52 | 1.08 | - |
| IB N | N-25 | 3-Dimethyl-4-phenyl-2-azetidinone | H/E=90/10 | 1.0 | 210 | 25 | 10.352 | 15.451 | 2.47 | 4.18 | 1.69 | 12.34 |
| IB N | N-26 | 3-Dimethyl-4-(2-furyl)-2-azetidinone | H/I=60/40 | 1.0 | 210 | 25 | 4.381 | 4.634 | 0.47 | 0.55 | 1.18 | 1.95 |
| IC | N-22 | 4-Ethenyl-2-azetidinone | H/I=60/40 | 1.0 | 210 | 25 | 8.000 | 9.134 | 1.58 | 1.95 | 1.23 | 3.67 |
| IC | N-23 | 1-[*tert-*Butyl(dimethyl)silyl]-4-methyl-2-azetidinone | H/I=60/40 | 1.0 | 210 | 25 | 5.007 | 5.101 | 0.62 | 0.65 | 1.05 | - |
| IC | N-24 | 1-Benzyl-3-ethyl-2-azetidinone | H/I=60/40 | 1.0 | 210 | 25 | 7.199 | 7.484 | 1.32 | 1.41 | 1.07 | 0.99 |
| IC | N-25 | 3-Dimethyl-4-phenyl-2-azetidinone | H/E=90/10 | 1.0 | 210 | 25 | 9.383 | 11.601 | 2.03 | 2.74 | 1.35 | 6.85 |
| IC | N-26 | 3-Dimethyl-4-(2-furyl)-2-azetidinone | H/I=60/40 | 1.0 | 210 | 25 | 5.657 | 6.617 | 0.82 | 1.13 | 1.38 | 4.44 |
| ID | N-22 | 4-Ethenyl-2-azetidinone | H/I=60/40 | 1.0 | 210 | 25 | 4.64 | - | 0.47 | - | 1.00 | - |
| ID | N-23 | 1-[*tert-*Butyl(dimethyl)silyl]-4-methyl-2-azetidinone | H/I=60/40 | 1.0 | 210 | 25 | 3.849 | - | 0.22 | - | 1.00 | - |
| ID | N-24 | 1-Benzyl-3-ethyl-2-azetidinone | H/I=60/40 | 1.0 | 210 | 25 | 5.768 | 5.901 | 0.83 | 0.87 | 1.05 | - |
| ID | N-25 | 3-Dimethyl-4-phenyl-2-azetidinone | H/E=90/10 | 1.0 | 210 | 25 | 6.644 | 6.825 | 1.10 | 1.16 | 1.05 | 0.89 |
| ID | N-26 | 3-Dimethyl-4-(2-furyl)-2-azetidinone | H/I=60/40 | 1.0 | 210 | 25 | 4.216 | 4.877 | 0.34 | 0.54 | 1.62 | 5.28 |
| IE | N-22 | 4-Ethenyl-2-azetidinone | H/I=60/40 | 1.0 | 210 | 25 | 4.883 | 4.97 | 0.57 | 0.60 | 1.05 | - |
| IE | N-23 | 1-[*tert-*Butyl(dimethyl)silyl]-4-methyl-2-azetidinone | H/I=60/40 | 1.0 | 210 | 25 | 4.553 | - | 0.46 | - | 1.00 | - |
| IE | N-24 | 1-Benzyl-3-ethyl-2-azetidinone | H/I=60/40 | 1.0 | 210 | 25 | 6.723 | - | 1.16 | - | 1.00 | - |
| IE | N-25 | 3-Dimethyl-4-phenyl-2-azetidinone | H/E=90/10 | 1.0 | 210 | 25 | 10.14 | 10.957 | 2.26 | 2.52 | 1.12 | 2.78 |
| IE | N-26 | 3-Dimethyl-4-(2-furyl)-2-azetidinone | H/I=60/40 | 1.0 | 210 | 25 | 4.706 | 4.927 | 0.51 | 0.58 | 1.14 | 1.59 |
| IF | N-22 | 4-Ethenyl-2-azetidinone | H/I=60/40 | 1.0 | 210 | 25 | 4.374 | 4.465 | 0.49 | 0.52 | 1.06 | - |
| IF | N-23 | 1-[*tert-*Butyl(dimethyl)silyl]-4-methyl-2-azetidinone | H/I=60/40 | 1.0 | 210 | 25 | 3.722 | - | 0.27 | - | 1.00 | - |
| IF | N-24 | 1-Benzyl-3-ethyl-2-azetidinone | H/I=60/40 | 1.0 | 210 | 25 | 5.095 | 5.234 | 0.74 | 0.79 | 1.06 | - |
| IF | N-25 | 3-Dimethyl-4-phenyl-2-azetidinone | H/E=90/10 | 1.0 | 210 | 25 | 9.191 | 10.211 | 2.13 | 2.48 | 1.16 | 3.79 |
| IF | N-26 | 3-Dimethyl-4-(2-furyl)-2-azetidinone | H/I=60/40 | 1.0 | 210 | 25 | 4.063 | 4.381 | 0.39 | 0.49 | 1.28 | 2.80 |
| IG | N-22 | 4-Ethenyl-2-azetidinone | H/I=60/40 | 1.0 | 210 | 25 | 4.415 | 4.475 | 0.53 | 0.55 | 1.04 | - |
| IG | N-23 | 1-[*tert-*Butyl(dimethyl)silyl]-4-methyl-2-azetidinone | H/I=60/40 | 1.0 | 210 | 25 | 3.767 | - | 0.31 | - | 1.00 | - |
| IG | N-24 | 1-Benzyl-3-ethyl-2-azetidinone | H/I=60/40 | 1.0 | 210 | 25 | 5.795 | 6.078 | 1.01 | 1.11 | 1.10 | 1.14 |
| IG | N-25 | 3-Dimethyl-4-phenyl-2-azetidinone | H/E=90/10 | 1.0 | 210 | 25 | 11.703 | 17.814 | 3.07 | 5.19 | 1.69 | 12.37 |
| IG | N-26 | 3-Dimethyl-4-(2-furyl)-2-azetidinone | H/I=60/40 | 1.0 | 210 | 25 | 4.278 | 4.752 | 0.49 | 0.65 | 1.34 | 3.24 |

**TABLE S3** (Continued)

| Column | Sample No. | Sample | Eluent*^a^* | Flow rate*^b^* | Detect*^b^* | Temp.*^b^* | t1 | t2 | k1 | k2 | *α* | *Rs* |
| --- | --- | --- | --- | --- | --- | --- | --- | --- | --- | --- | --- | --- |
| IH | N-22 | 4-Ethenyl-2-azetidinone | H/I=60/40 | 1.0 | 210 | 25 | 13.445 | 18.031 | 3.44 | 4.96 | 1.44 | 6.11 |
| IH | N-23 | 1-[*tert-*Butyl(dimethyl)silyl]-4-methyl-2-azetidinone | H/I=60/40 | 1.0 | 210 | 25 | 3.727 | 4.425 | 0.23 | 0.46 | 2.00 | 4.97 |
| IH | N-24 | 1-Benzyl-3-ethyl-2-azetidinone | H/I=60/40 | 1.0 | 210 | 25 | 8.966 | 12.909 | 1.96 | 3.27 | 1.66 | 8.30 |
| IH | N-25 | 3-Dimethyl-4-phenyl-2-azetidinone | H/E=90/10 | 1.0 | 210 | 25 | 24.01 | 27.272 | 6.93 | 8.01 | 1.16 | 3.35 |
| IH | N-26 | 3-Dimethyl-4-(2-furyl)-2-azetidinone | H/I=60/40 | 1.0 | 210 | 25 | 12.776 | 35.996 | 3.22 | 10.90 | 3.38 | 17.89 |
| AS-H | N-22 | 4-Ethenyl-2-azetidinone | H/I=60/40 | 1.0 | 210 | 25 | 16.311 | 22.877 | 4.22 | 6.33 | 1.50 | 6.77 |
| AS-H | N-23 | 1-[*tert-*Butyl(dimethyl)silyl]-4-methyl-2-azetidinone | H/I=60/40 | 1.0 | 210 | 25 | 3.806 | 4.774 | 0.22 | 0.53 | 2.42 | 5.34 |
| AS-H | N-24 | 1-Benzyl-3-ethyl-2-azetidinone | H/I=60/40 | 1.0 | 210 | 25 | 10.414 | 16.454 | 2.34 | 4.27 | 1.83 | 8.99 |
| AS-H | N-25 | 3-Dimethyl-4-phenyl-2-azetidinone | H/E=90/10 | 1.0 | 210 | 25 | 25.741 | 29.377 | 7.25 | 8.41 | 1.16 | 3.22 |
| AS-H | N-26 | 3-Dimethyl-4-(2-furyl)-2-azetidinone | H/I=60/40 | 1.0 | 210 | 25 | 16.274 | 46.712 | 4.21 | 13.96 | 3.31 | 14.97 |

*^a^* “H” means *n*-hexane, “E” means ethanol and “I” means 2-propanol.

*^b^* Flow rate’s unit is “mL/min”, Detect’s unit is “nm UV” and Temp.’s unit is “℃”.
